# Zeeman Level Anti-Crossing and Slow Magnetic Relaxation in an S‑Functionalized Abnormal NHC-Supported Linear Co^II^_3_ Single-Molecule Magnet

**DOI:** 10.1021/acs.inorgchem.6c01997

**Published:** 2026-08-11

**Authors:** Sangita Mondal, Sunil Kumar, Sujit Das, Liviu Ungur, Björn Schwarz, Kartik Chandra Mondal

**Affiliations:** † Department of Chemistry, 37268 Indian Institute of Technology, Madras, Chennai 600036, India; ‡ Office MD1-05-03A, Department of Chemistry, 37580 National University of Singapore, Singapore 117543; § Institute for Applied Materials (IAM), Karlsruhe Institute of Technology (KIT), Hermann-von-Helmholtz-Platz 1, 76344 Eggenstein-Leopoldshafen, Germany

## Abstract

A novel trinuclear cobalt­(II) complex, [(S-MIC)_2_Co­(II)_3_I_2_(μ_2_-Cl)_4_(THF)_2_]·4THF (**1**), supported by a zwitterionic ylide-type sulfur-functionalized abnormal N-heterocyclic carbene (S-MIC) ligand, has been synthesized and characterized as the first S-functionalized aNHC-supported cobalt­(II) single-molecule magnet (SMM). X-ray diffraction reveals a linear trinuclear Co^II^_3_ core in which the central Co­(II) adopts a distorted octahedral geometry, while each terminal Co­(II) is distorted tetrahedral, coordinated by two μ_2_-chlorides, one terminal iodide, and one sulfur donor from S-MIC. The *χT* value of 10.3 cm^3^ K mol^–1^ at 300 K indicates significant orbital angular momentum contributions. Ab initio SA-CASSCF/NEVPT2 calculations with POLY_ANISO analysis reveal weak antiferromagnetic coupling (*J* = −3.39 cm^–1^; *J*′ = −1.15 cm^–1^) and axial anisotropy (*D* values of −66.81 cm^–1^ for the central and −20.46 cm^–1^ for the terminal Co­(II)). This weak coupling causes level anti-crossing of low-lying Zeeman levels, yielding open hysteresis and a marked magnetization-slope change near 35 kOe. Isothermal DC magnetization at 35 kOe confirms slow relaxation, with a τ of 451(3) s at 1.8 K. AC susceptibility measurements at 20 DC fields yield an effective barrier (*U*_eff_ = 25(20) K) under a QTM+Orbach model, discussed alongside ab initio calculations, underscoring the potential of S-functionalized carbene ligands for tuning coordination geometries and constructing multinuclear Co-based SMMs.

## Graphical abstract

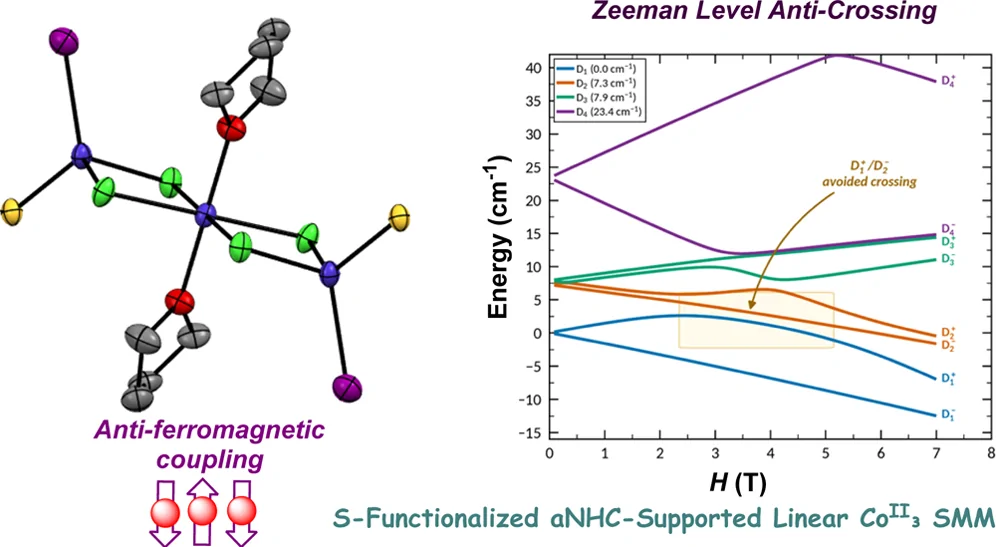

## Introduction

The miniaturization of electronic components in modern technology has driven advances in nanomagnetism,^1^ shifting the focus from bulk magnets to nanoscale systems with enhanced performance and storage density.^2^ Among these, single-molecule magnets (SMMs)^3^ stand out as molecular-scale units that exhibit slow magnetic relaxation with magnetic bistability,^4,5^ arising from a large spin ground state and strong magnetic anisotropy, which together create an energy barrier to magnetization reversal (*U*_eff_).^6^ The first SMM, [Mn_12_O_12_(O_2_CCH_3_)_16_(H_2_O)_4_]·2HOAc·4H_2_O, discovered in 1988 with a blocking temperature (*T*_B_) of 2.5 K,^7^ marked the bridge between classical and quantum magnetism. Since then, numerous SMMs based on transition metals^8^ and lanthanides^9^ have been developed, offering precise structural control for exploring quantum effects like tunneling^10^ and coherence.^11^ Their discrete nature makes them promising for applications in high-density data storage,^12^ quantum computing,^13^ spintronics,^14^ and magnetic refrigeration.^15^ Current efforts aim to increase operating temperatures and optimize magnetic behavior through ligand design, new metal centers, and insights into structure–property relationships.

For a molecule to act as an effective SMM, it must possess a large spin ground state (*S*),^16^ strong magnetic anisotropy (*D*), and a minimal transverse anisotropy (*E*).^17,18^ The barrier (*U*_eff_) depends on the zero-field splitting (ZFS) parameter, *D*, being especially crucial. For easy-axis anisotropy, the barrier is given by the equations *U*_eff_ = |*D*|*S* ^2^ for integer spins and *U*_eff_ = |*D*|(*S* ^2^ – 1/4) for half-integer spins, making a large negative *D* highly desirable.^19^ The slow relaxation of magnetization in SMMs occurs via several mechanisms: at high temperatures through the thermally activated Orbach process, where the spin system is excited to a higher-energy state and then relaxes back to the ground state, following the Arrhenius dependence, τ = τ_0_ exp­(*U*_eff_)/*k*_B_ *T*);^6,20^ and at very low temperatures via quantum tunneling of magnetization (QTM),^10^ where the magnetization tunnels through the energy barrier or the Raman process.^21,22^ Minimizing the effects of QTM is a primary goal of SMM design, often achieved by reducing the transverse anisotropy (*E*), which creates pathways for tunneling.^18^

Since the discovery of SMMs, efforts have largely focused on increasing the total spin (*S*) to enhance the spin reversal barrier (*U*_eff_) and blocking temperature (*T*_B_).^23^ However, theoretical studies have shown that increasing *S* beyond a certain point offers no significant gain as the axial zero-field splitting parameter (*D*) shows an inverse dependence on *S* ^2^.^24^ This has shifted the focus to enhancing the axial anisotropy parameter (*D*) of single-ion magnets (SIMs),^25^ which make up a prominent subclass of SMMs. While lanthanide-based systems have achieved remarkable barriers, they often face challenges related to strong coupling and synthetic robustness.^26,27^ This has driven renewed interest in transition metal complexes, which can provide an attractive alternative because of their more predictable exchange pathways and robust synthetic chemistry.^28^

Among them, the Co­(II) ion has been of particular interest as a new anisotropy source in recent years.^29,30^ A linear trinuclear Co­(II) complex reported by Zabala-Lekuona, Seco, Colacio, and co-workers in 2023 with triple-phenolate bridges and distinct coordination geometries shows slow magnetic relaxation and open hysteresis at zero field due to medium-to-strong antiferromagnetic coupling.^31^ Notably, while this system exhibits antiferromagnetic coupling similar to that of the present complex (**1**), it differs in the orientation of its local magnetic anisotropy axes. Earlier, in 2015, Zhang, Gao, and co-workers described a linear Co­(II) trinuclear complex showing SMM properties with ferromagnetic exchange and a smaller energy barrier under an applied field.^32^ More recently, in 2025, we reported an anionic linear siloxane-bridged Co_3_ complex displaying SMM behavior with strong ferromagnetic coupling and a higher energy barrier (*U*_eff_ = 94(4) K).^33^ Although this complex shares a comparable coordination environment with the present complex (**1**), it displays contrasting magnetic exchange interactions. In contrast to the previously reported O-bridged systems, the current work (Figure 1) presents a linear trinuclear Co_3_ complex exhibiting SMM property with antiferromagnetic interactions, highlighting the important role of magnetic exchange and anisotropy in these cobalt systems, where three Co centers are aligned in a colinear topology bridged by chlorido ligands. These findings highlight that controlling coordination geometry, bridging connectivity, and metal–metal distances is essential to maintain slow magnetic spin–lattice relaxation. A comparison of magnetic and structural parameters for reported trinuclear Co­(II) complexes exhibiting SMM behavior is provided in Table S6.

**Figure 1:**
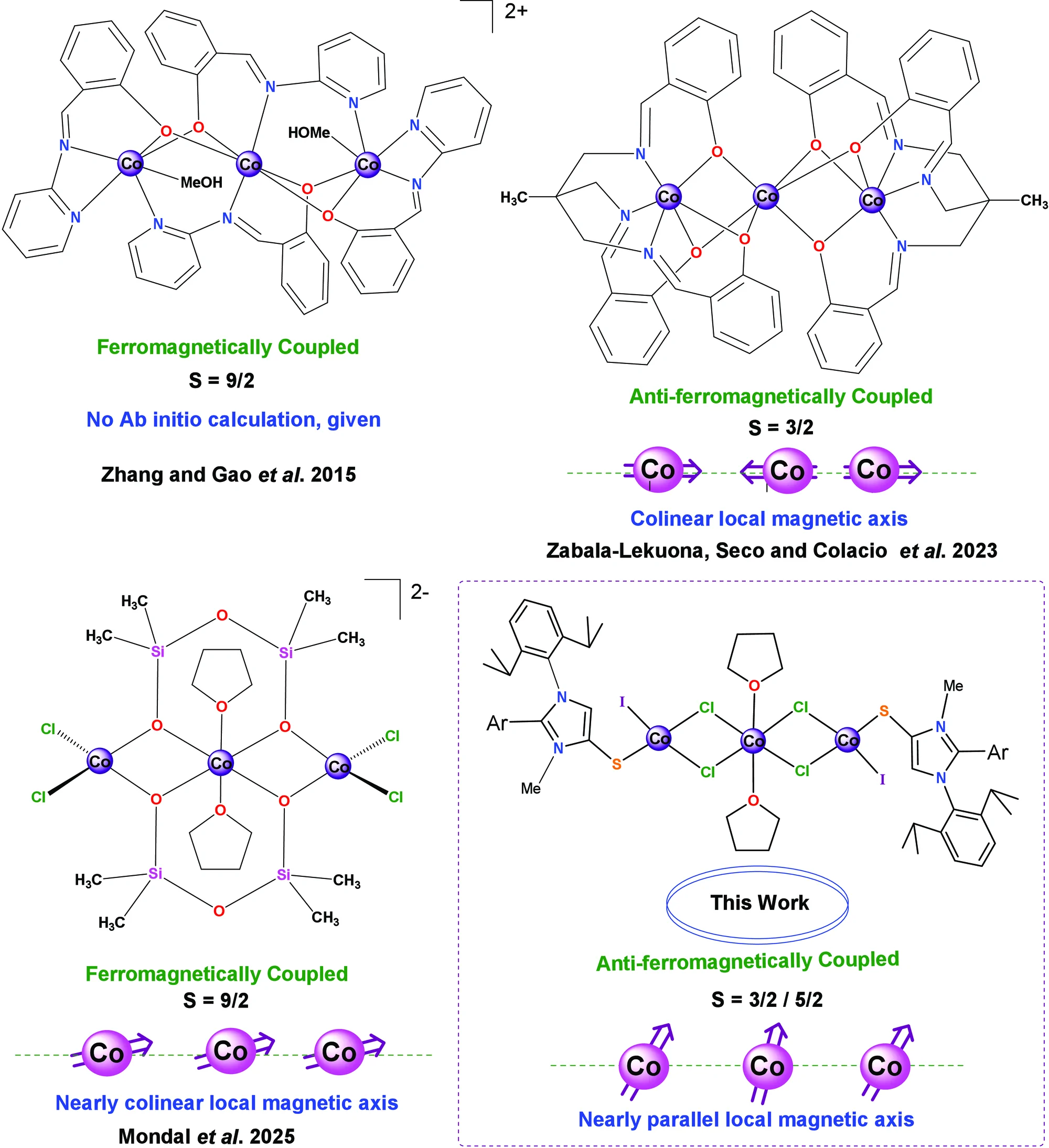
Selected linear trinuclear Co­(II)_3_ complexes exhibiting SMM behavior: prior O/N-bridged examples alongside the current Cl-bridged system. This comparison highlights differences in magnetic coupling, bridging ligand identity, and the orientation of local anisotropy axes relative to the molecular axis.

Ligand design and structural tuning are therefore key strategies to achieve slow magnetic relaxation and bistability in polynuclear cobalt complexes being at the core of advancing SMM chemistry.^34^ In this regard, N-heterocyclic carbenes (NHCs),^35^ known for strong σ-donation and tunable structures, stabilize many transition metal complexes.^36–39^ Abnormal NHCs (aNHCs), with carbene formation at unusual ring positions (e.g., C4 or C5 instead of C2 in imidazole-based carbenes), offer even stronger σ-donation and unique electronic properties and mesoionic character,^40,41^ making them promising for tuning metal complex anisotropy. In 2009, Frenking, Bertrand, and co-workers reported the synthesis of metal-free abnormal N-heterocyclic carbenes (aNHCs)^42^ via C5 deprotonation of imidazolium salts and their stable complexes with gold and CO_2_. Later, in 2020, Ghadwal and co-workers achieved a Ni-catalyzed intramolecular 1,2-aryl migration of C5-protonated aNHCs (iMICs),^43^ enabling access to super iMICs that formed stable complexes with Au, Se, and Pd. More recently, in 2022, they synthesized mesoionic dithiolates (MIDts) from 1,3-imidazole-based anionic dicarbenes (ADCs) and their germanium complexes,^44^ showcasing the versatility of aNHC scaffolds. Despite their potential, cobalt complexes with aNHC ligands showing SMM behavior are rare.^45,46^ In 2015, Rajaraman, Shanmugam, and co-workers reported tetrahedral Co­(II) complexes with exocyclic mesoionic ligands exhibiting SIM behavior. Sulfur functionalization in the ligand framework notably enhanced the system’s magnetic anisotropy.^47^

Therefore, functionalizing aNHCs with sulfur can add flexibility and promote metal exchange interactions, creating a powerful design strategy for multinuclear cobalt SMMs. In this work, we present the synthesis and characterization of a novel trinuclear cobalt­(II) complex supported by a ylide-type (S-functionalized aNHC) ligand (S-MIC) for the first time. To the best of our knowledge, this is the first S-functionalized ylide-type aNHC-supported cobalt­(II) system exhibiting SMM behavior. Our findings highlight how S-functionalized aNHCs can be employed to bridge ligand design and molecular magnetism, opening new directions for exploiting abnormal carbene chemistry in the development of SMMs.^48^ In addition, we examined the magnetic properties through comprehensive ab initio calculations and susceptibility measurements using both direct current (dc) and alternating current (ac) methods.

## Results and Discussion

Cobalt complex **1** was prepared by the reaction of the S-MIC ligand with anhydrous CoCl_2_ in dry THF (Scheme 1) under inert conditions, followed by workup and crystallization from a THF/*n*-hexane mixture to afford green crystalline material suitable for single-crystal (Figure S1) X-ray diffraction. Complex [(S-MIC)_2_Co­(II)_3_I_2_(μ_2_-Cl)_4_(THF)_2_]·4THF (**1**) crystallizes in the monoclinic *P*2_1_/*n* space group (Table S1). The molecular structure of complex **1** features a linear trinuclear Co^II^_3_ core, centered about an inversion center (*i*), located at the central cobalt ion (Co2) (Figure S2). The three Co^II^ centers are aligned linearly and bridged by four μ_2_-chlorido ligands, with each chloride bridging two adjacent Co^II^ metal centers (Co1). The central cobalt ion (Co2) adopts a distorted octahedral geometry, while the two terminal cobalt ions (Co1) exhibit distorted tetrahedral geometries, a coordination arrangement similar to that observed in the siloxane-bridged Co_3_ complex reported by us.^33^

**Scheme 1:**
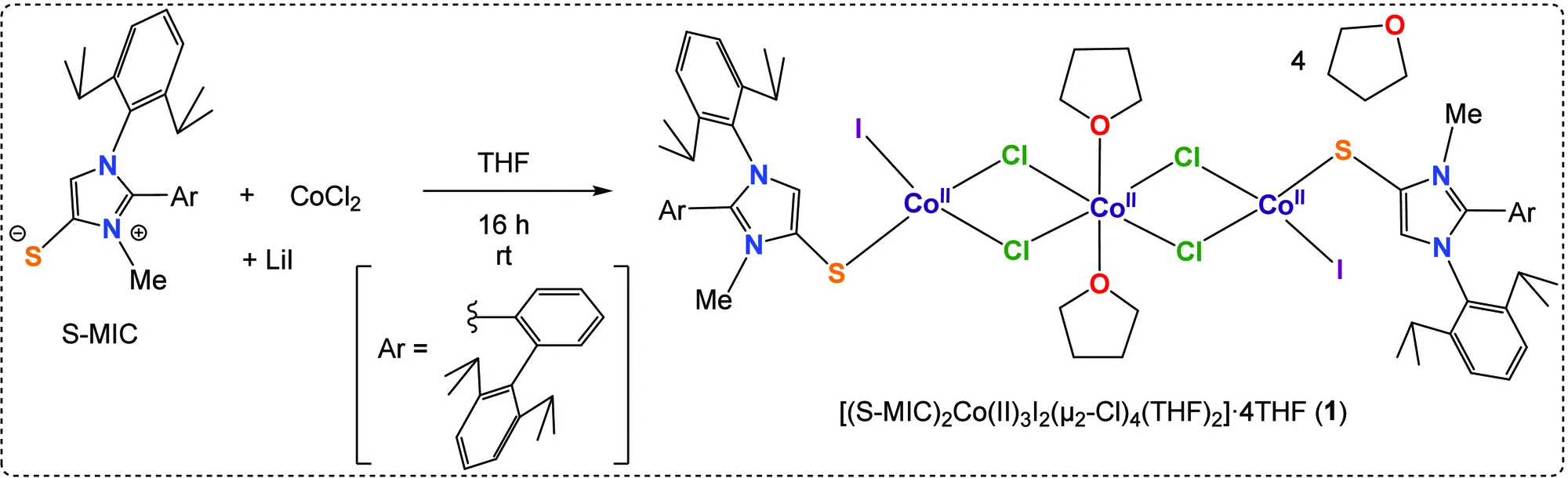
Synthesis of Linear Co­(II)_3_ Complex **1** in the Presence of LiI

At the Co2 center, the equatorial plane comprises four μ_2_-chlorido ligands, while two THF molecules coordinate axially via oxygen donors, resulting in an axially compressed distorted octahedron. The Co2–O bond lengths of 2.049(4) Å are slightly longer than the apical Co–O distance of 2.022(3) Å reported for the linear Co_3_ complex by Zhang, Gao, and co-workers.^32^ The Co–Cl distances in **1** fall within the ranges of 2.2948(14)–2.3120(14) and 2.4838(12)–2.4841(11) Å. The Co1–S1 bond length of 2.2938(14) Å is comparable to that reported for a Co­(II)–thiourea complex (2.309(2) Å), noted for its remarkable bioactivity.^49^ Similarly, the Co1–I1 distance of 2.5724(8) Å in complex **1** lies within the expected range for Co­(II)–I bond lengths in pyridine- and redox-active 1,2-bis­(arylimino)­acenaphthene-based Co­(II) complexes exhibiting SIM behavior.^50^

In agreement with the imposed symmetry, the two THF ligands bound to Co(2) are arranged linearly, with an O1^i^–Co2–O1 angle of 180°. The *trans* Cl1^i^–Co2–Cl1 and Cl2–Co2–Cl2^i^ angles are also 180° and 180.00(3)°, respectively. The intramolecular Co···Co separations are 3.281 Å (Co1···Co2) and 6.562 Å (Co1···Co1) (Figure 2), values that are consistent with those observed in related linear Co_3_ complexes showing SMM properties.^33^ Each terminal Co1 center is tetrahedrally surrounded by two μ_2_-chlorido ligands, one terminal iodide ligand, and one sulfur donor atom from the S-MIC (ylide-type S-functionalized abnormal NHC carbene-based) ligand, leading to a distorted tetrahedral coordination geometry. The S1–Co1–I1 bond angle is measured at 116.85(4)°. The two terminal cobalt centers (Co1) are each coordinated by two μ_2_-chlorido ligands, one terminal iodide ligand, and one sulfur donor atom from the S-MIC ligand, an S-functionalized abnormal NHC carbene-based ligand. This coordination environment results in a distorted tetrahedral geometry around both Co1 centers. All bond lengths and bond angles are summarized in Tables S2 and S3.

**Figure 2:**
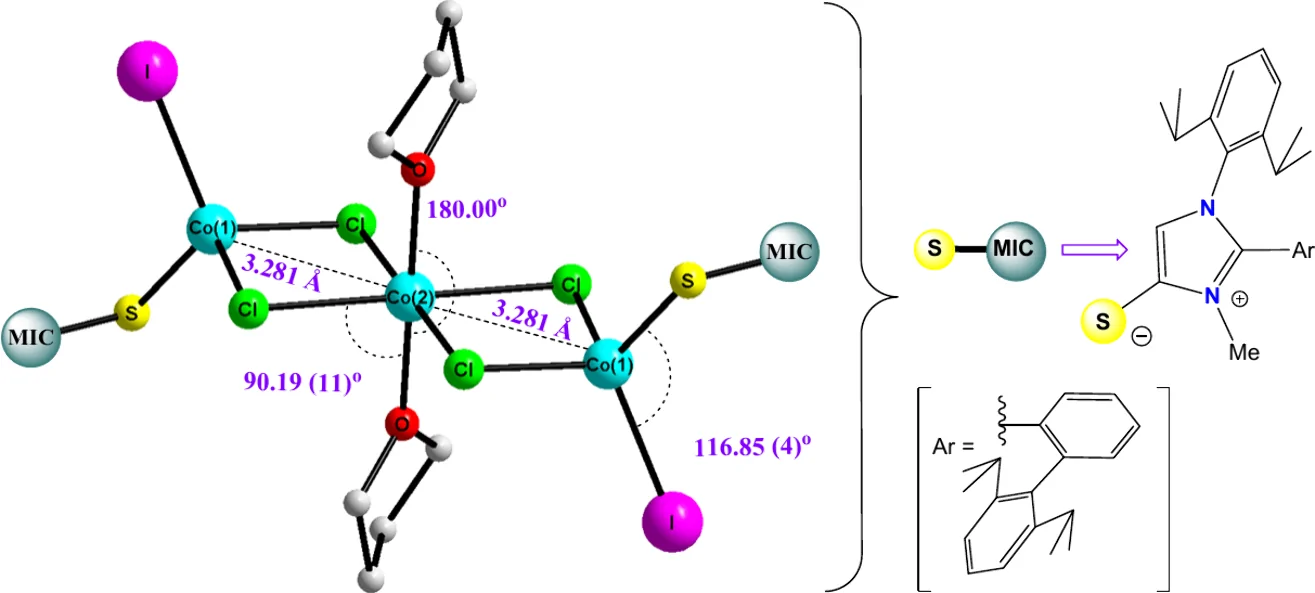
Structural framework for complex **1** showing the intramolecular Co···Co distances and selected bond angles.

In the UV–visible spectrum, the complex exhibits distinct absorption peaks at 326 and 679 nm in THF (Figure S3). The IR spectrum (Figure S4) reveals high-intensity bands at 1626 and 809 cm^–1^, which can be attributed to characteristic C=C/C=N and C–S stretching vibrations, respectively.^51^ Furthermore, the Raman spectrum (Figure S5) displays several characteristic metal–ligand vibrational features. Bands at 164 cm^–1^ are assigned to Co–S–C deformations, while distinct metal–halogen stretching modes appear at 213, 267, and 302 cm^–1^, consistent with reported ranges for μ_2_-Co–Cl and Co–I stretching vibrations. Additional low-frequency modes include Co–S stretching at 415 and 443 cm^–1^ and a Co–O stretching vibration at 507 cm^–1^, confirming the coordination of sulfur from the S-MIC ligand and oxygen from THF. Together, these assignments provide clear evidence for multiple types of metal–ligand bonding (Co–S, Co–Cl, Co–I, and Co–O) within the cluster, while higher-frequency vibrations correspond to C–S, aromatic, imidazole, and overtone modes. All peaks have been carefully assigned within the reported reference ranges, ensuring reliable interpretation of the vibrational data, and are mentioned in Table S4. The HRMS analysis of complex **1** in DCM reveals the presence of only the ligand fragment, which is likely a result of subsequent decomposition of the complex and dissociation into the free ligand in this solvent (Figure S6). Cyclic voltammetry measurement (Figure S7) in a THF solution of 0.1 M [*n*-Bu_4_N]­ClO_4_ electrolyte shows a weak, irreversible cathodic response around −2.35 V, which can be assigned to a ligand-based redox process.^20,21^

Complex **1** is composed of three Co­(II) ions (three *S*_Co_2_^+^_ = 3/2). The EPR measurements at rt showed a weak signal near *g* = 2 without a forbidden half-field signal. However, on cooling down to 5 K, it showed an intense EPR signal near *g* = 2.11 in the solid state with a strong half-field signal for the forbidden transition (*Δm*_S_ = ±2) near *g* ≈ 4 corresponding to the ground state spin *S* = 3/2 of **1** (Figure 3, top). The corresponding Mulliken spin densities at the BP86-D3­(BJ)/Def2-TZVP level of theory are shown in Figure 3 (panels a and b, middle). Consideration of the hyperfine coupling constant (*A* = 700 MHz) due to ^59^Co (*I* = 7/2) improved the fitting of the EPR plot (blue line vs red line in the top panel of Figure 3). The *D* and *E* parameters of **1** could not be extracted from the EPR plot since the data were measured on a powdered sample, suggesting oriented single-crystal EPR measurements will be necessary, which is outside the scope of this work.

**Figure 3:**
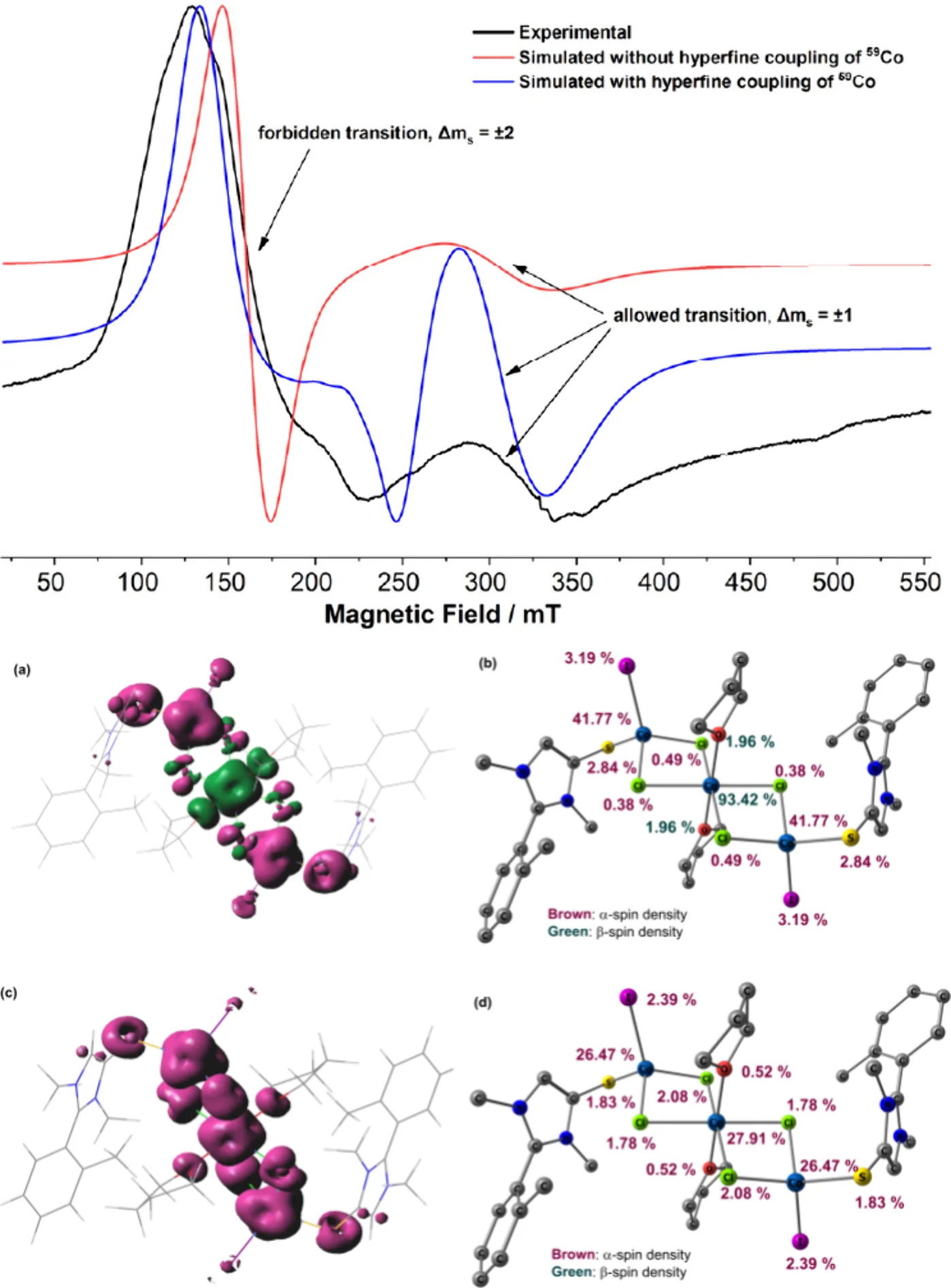
Experimental (black line) and simulated (red and blue lines) X-band EPR spectra of complex **1** at 5 K in the solid state (9.3882 GHz); *g* = 2.11 (top). The simulation is done using EasySpin.^52^ Mulliken α- and β-spin densities of complex **1′** in the (a) *S* = 3/2 (**1a′**) and (c) *S* = 9/2 (**1b′**) states (violet, α-spin density; green, β-spin density) and plots of the percentage of α- and β-spins on atoms of (b and d) **1′** at the BP86-D3­(BJ)/Def2-TZVP level of theory. H atoms have been omitted for the sake of clarity. Dip groups are substituted with methyl groups. Magnetic measurements showed a population of mostly *S* = 3/2 at a lower magnetic field (<3 T) (see Figures 6 and 7).

The experimental powder XRD pattern (Figure S9) of complex **1** matches well with the simulated pattern derived from single-crystal XRD data, confirming the phase purity and crystallinity of the bulk material. The alignment of peak positions indicates that the crystal structure obtained from the single crystal is representative of the powdered sample. Minor differences in intensity may arise from the preferred orientation or instrumental factors but do not affect the overall confirmation of the synthesized phase.

The XPS spectra of complex **1** show characteristic peaks corresponding to all constituent elements, including Co, C, N, O, Cl, I, and S, with detailed plots (Figure S8) and peak assignments provided in Table S5, consistent with literature-reported binding energy ranges. The Co 2p spectrum, described herein (Figure 4), exhibits six distinct peaks at binding energies of 802.3, 795.9, 793, 785.8, 780.2, and 778.1 eV, characteristic of Co^2+^ ions exhibiting spin–orbit splitting. The peaks at 778.1 and 780.2 eV correspond to the Co 2p_3/2_ component, while those at 793 and 795.9 eV belong to the Co 2p_1/2_ component, consistent with a typical spin–orbit energy separation of approximately 15 eV.^53,54^ The features at 785.8 and 802.3 eV are shakeup satellite peaks, indicative of high-spin Co^2+^ species with electron correlation effects.^55^ The multiplet feature of the XPS spectrum confirms the coordination environments of three Co centers (central Co, Oh; two terminal Co atoms, Td).^56^ These peak positions and satellite features align well with reported literature values for Co^2+^ in similar coordination environments, confirming the oxidation state and coordination geometry of the cobalt centers in the complex.

**Figure 4:**
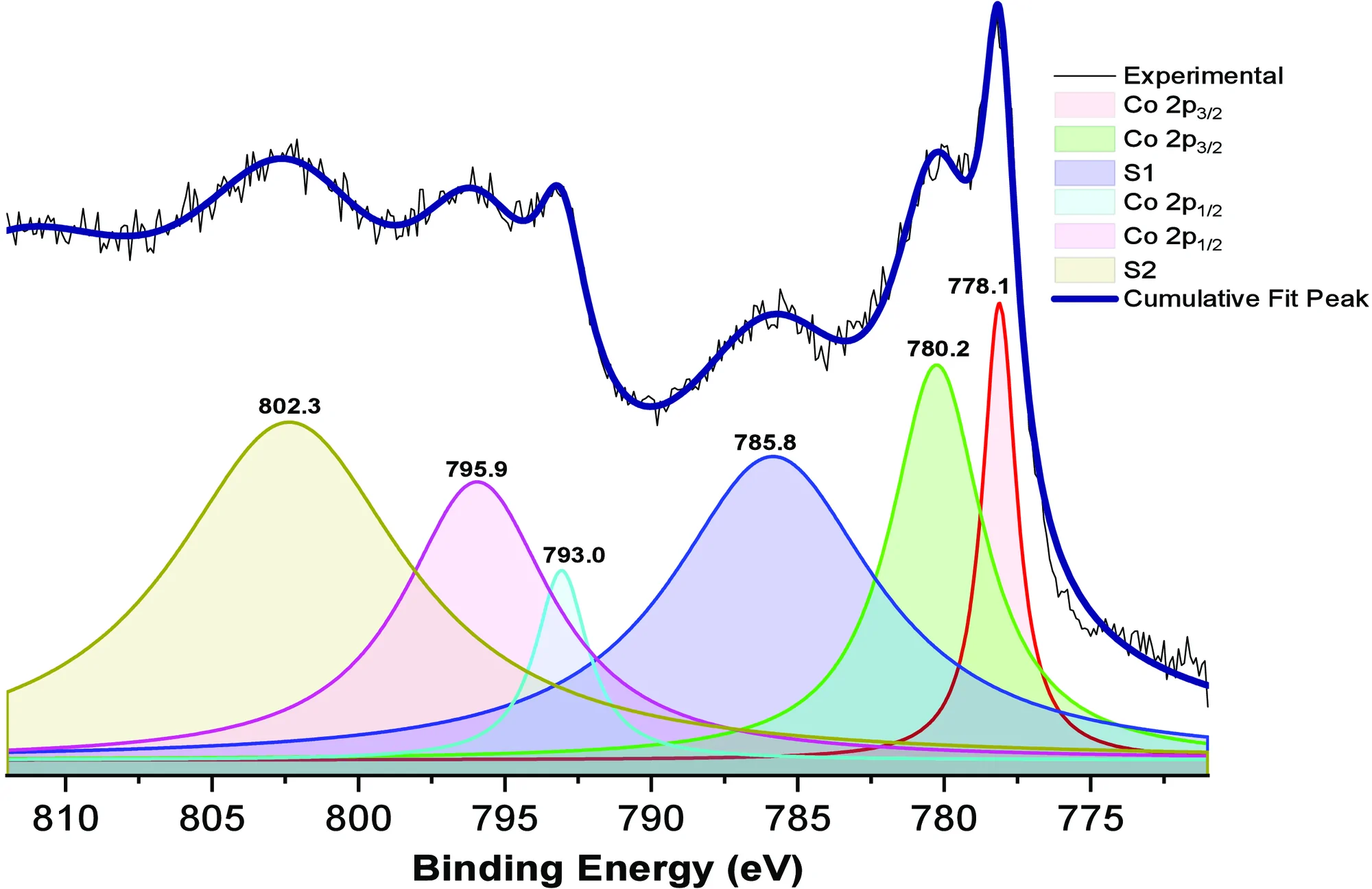
XPS spectrum (black line) of the Co 2p region of complex **1**. Lines with corresponding areas under them shown with different colors (Co 2p_3/2_, Co 2p_1/2_, satellites, and fitting).

TGA analysis of complex **1** (Figure S10) shows that it exhibits thermal stability up to 360 °C, with a minor weight loss at around 150 °C, likely due to solvent evaporation, indicating its suitability for applications requiring moderate thermal endurance.

### Static Magnetic Properties

At 300 K, the measured magnetic susceptibility (*χT*) value of approximately 10.3 cm^3^ K mol^–1^ is significantly higher than the spin-only magnetic moment expected for three free Co­(II) magnetic centers (*S* = 3/2), calculated as 3 × 1.88 cm^3^ K mol^–1^ = 5.64 cm^3^ K mol^–1^ due to significant contributions from angular momentum (Figure 5a–c). Upon cooling below 100 K, the *χT* value decreases markedly due to single-ion anisotropy effects and antiferromagnetic coupling as outlined below, reaching a minimum value of 2.23 cm^3^ K mol^–1^ at 2 K. Correspondingly, the magnetization versus magnetic field (*M*–*H*) plots (Figure 5d) do not reach saturation even at 2 K and 70 kOe, with the magnetization attaining a value of only 4.08 μB, which is even well below the theoretical spin-only saturation value of 9 μB for three hypothetically independent Co­(II) ions with *S* = 3/2.

**Figure 5:**
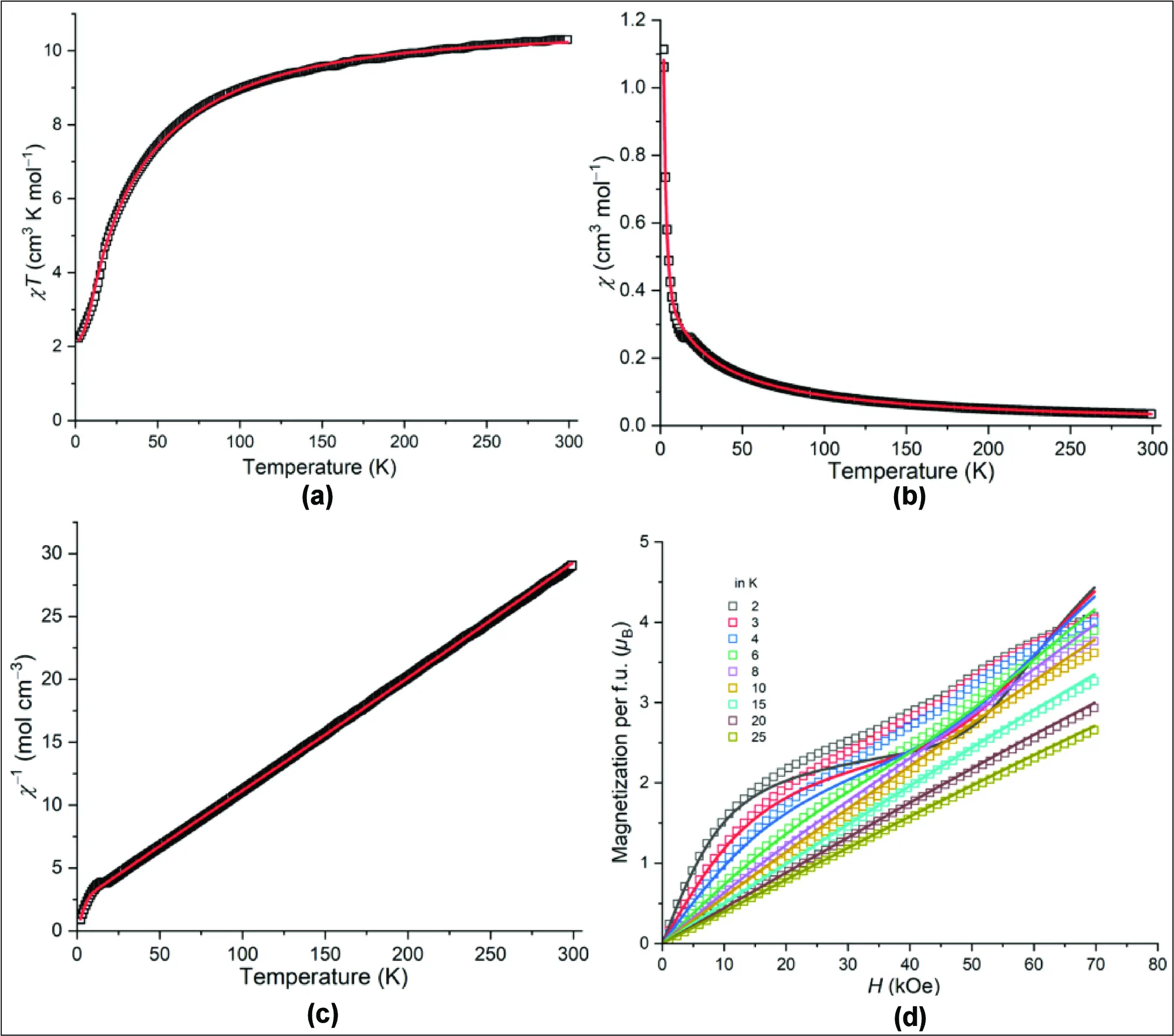
Experimental DC magnetic data for **1** (empty symbols) together with fits according to eq 1 (lines): (a) *χT* vs *T*, (b) χ vs *T*, (c) χ^–1^ vs *T*, and (d) magnetization vs field.

The experimental magnetic susceptibility and field-dependent data were analyzed by fitting with the anisotropic spin Hamiltonian

1 $$Ĥ=-J\left({Ŝ}_{1}{Ŝ}_{2}+{Ŝ}_{2}{Ŝ}_{3}\right)+\overset{3}{\underset{i=1}{\sum }}\left[D\left({{Ŝ}_{\mathrm{zi}}}^{2}-S\left(S+1\right)/3\right)+E\left({{Ŝ}_{\mathrm{xi}}}^{2}-{{Ŝ}_{\mathrm{yi}}}^{2}\right)+\beta \mathrm{H⃗}\mathrm{g̿}{Ŝ}_{i}\right]$$

which includes the isotropic exchange interaction parameter (*J*) between the center Co2 ion and the two outer Co1 ions, axial and transverse zero-field splitting (ZFS) parameters (*D* and *E*, respectively), β = 1/(*k*_B_ *T*), magnetic field **H⃗**, and the Landé **g** tensor (**g̿**). For simplification, the next-nearest-neighbor exchange interactions and transverse ZFS component *E* were set to zero, while the **g** tensor was constrained to *g*_*x*_ = *g*_*y*_ = *g*_⊥_ and *g*_*z*_ = *g*_∥_. In addition, *g* factors *g*_⊥_ and *g*_∥_ and axial ZFS parameter *D* have been constrained to the same value for the three Co­(II) magnetic centers that were represented by an effective spin *S* = 3/2. Figure 5 presents the best fit of eq 1 with strong simplifications to the experimental susceptibility (*χT*) versus temperature (*T*) and magnetization (*M*) versus field scan (*H*) data.

From the optimized parameters, isotropic exchange constant *J* was determined to be −1.105(5) cm^–1^, indicating a dominant but weak antiferromagnetic (AFM) coupling between the center Co2 and the two outer Co1 ions. The axial single-ion anisotropy, represented by *D*, was refined to −30.8(3) cm^–1^, while the *g*_⊥_ and *g*_*z*_ values were determined to be 2.780(5) and 2.680(7), respectively. These increased values compared to the free electron *g* factor (*g*_e_ ≈ 2) indicate the strong contribution from orbital angular momentum. The steep decrease in *χT* below about 100 K and the fact that the magnetization does not saturate even at 2 K and 70 kOe are ascribed predominantly to the strong axial anisotropy and further to AFM coupling.

The corresponding Zeeman energy diagram, calculated using these refined parameters, shows three important excited doublet states that are separated from the ground state by only small energy gaps. In total, (2*S* + 1)^3^ = 64 energy states are present; most of them are not important since they are far from the thermal population. The ground state is a doublet that predominantly comprises basis states |+–+⟩ (99.98%) and |−+–⟩ (99.98%), where the plus and minus signs stand for **m**_**S**_ = |+3/2⟩ and **m**_**S**_ = |−3/2⟩, respectively, associated with the first, second, and third Co­(II) ion. About 10 cm^–1^ above the ground state, there are two quasi-degenerate doublets corresponding to the first and second excited states with nearly equal energy. The first consists mainly of |−++⟩ (99.98%) and |+––⟩ (99.98%), and the second |++–⟩ (99.99%) and |−–+⟩ (99.99%). These excited states differ from the ground state by a single unsatisfied exchange interaction. Notably, the ground and first/second excited doublets share the same total spin *S*_*n*_ = 3/2 and display identical initial slopes in the Zeeman diagram, reflecting identical permanent magnetic moments (Figure 6). The third excited state, located approximately 20 cm^–1^ above the ground state, is also a doublet and is composed purely of |+++⟩ (100%) and |−––⟩ (100%) basis states within the framework of this simple phenomenological spin Hamiltonian approach. The next excited state (not shown any more in the inset Zeeman diagram) lies about 64 cm^–1^ above the ground state and involves states **m**_**S**_ = |±1/2⟩ that exhibit a much larger energy contribution arising from the axial single-ion anisotropy (ZFS parameter *D*) compared to the **m**_**S**_ = |±3/2⟩ states.

**Figure 6:**
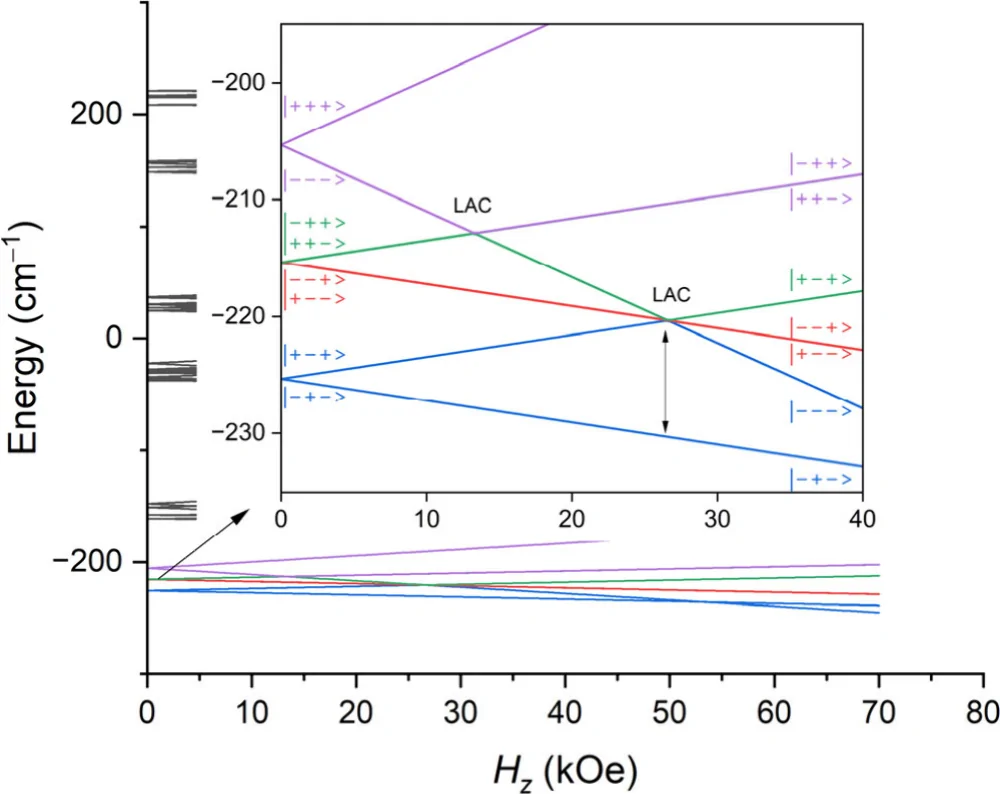
Zeeman diagram calculated for complex **1** according to fitted parameters (eq 1). The inset shows an enlargement of the most important low-lying Zeeman levels exhibiting level anti-crossing (LAC) behavior. See also Figure 14 for the corresponding diagram obtained from more sophisticated ab initio calculations.

The level anti-crossing (LAC) behavior as calculated for the important low-lying Zeeman levels, around a magnetic field of μ_0_ *H*_LAC_ ≈ 3 T, for instance, due to the comparably weak AFM exchange coupling (see Figure 6) has some remarkable consequences: From 2 to ∼8 K, the magnetization versus magnetic field loop scans that were obtained by sweeping the field at a rate of 100 Oe s^–1^ exhibit an open hysteresis around 35 kOe (Figure 7), indicating very slow magnetic relaxation to be present here with relaxation times on a time scale of seconds (this will be investigated in more detail in the section about the complex’s dynamic magnetic properties). In this field range, the value of magnetization at a given field specifically depends on whether it has been measured while the field was being increased or decreased (arrows in Figure 7). In the same magnetic field region, the magnetization’s slope changes significantly. An explanation for these experimental findings is given in Theoretical Calculations based on the results from more sophisticated ab initio calculations.

**Figure 7:**
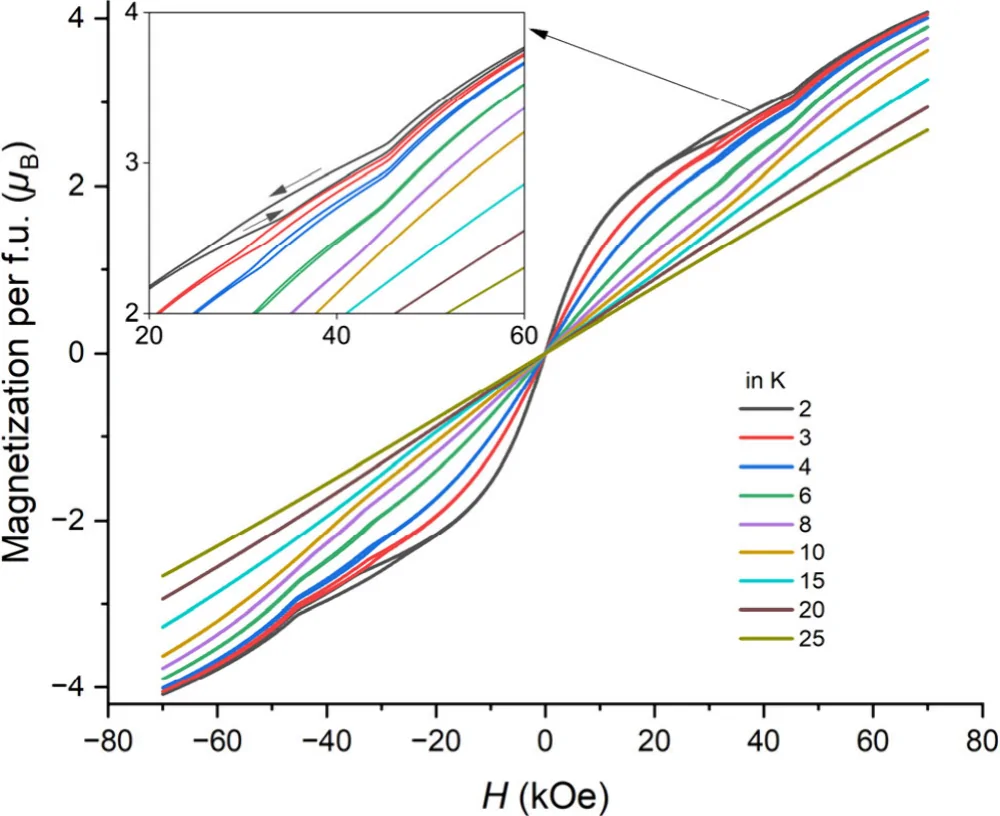
Full loop field sweep scans of complex **1** at a field scan rate of 100 Oe s^–1^. The black arrows indicate whether the data were obtained while the field was being increased or decreased.

### Dynamic Magnetic Properties

The dynamic magnetic properties, i.e., spin–lattice relaxation, of complex **1** have been investigated under two different DC field regimes. First, a comprehensive investigation of slow magnetic relaxation was performed using AC susceptibility at 20 different DC magnetic fields ranging from 0 to ∼8 kOe (with a log-distribution of applied DC fields). In addition, the slow magnetic relaxation that is indicated by the opening of the magnetization versus field curves (hysteresis) around 35 kOe was investigated by time-dependent DC magnetometry.

Figure 8 displays representative out-of-phase AC susceptibility *χ″* versus frequency scans together with the corresponding Cole–Cole plots recorded at *H*_DC_ = 0 and at *H*_DC_ = 2057 Oe (the complete set of *χ′* and *χ″* vs frequency curves for 20 different DC fields is provided in Figure S15). The maxima observed in the *χ″* versus frequency profiles confirm the presence of slow magnetic relaxation, and these peaks shift to higher frequencies as the temperature increases from 1.8 to 2.8 K (in zero field). The accessible AC excitation frequency window, the frequency-dependent signal accuracy, and the experimental temperature range (with a minimum temperature of 1.8 K here) together define the parameter space within which the slow relaxation of this complex can be probed.

**Figure 8:**
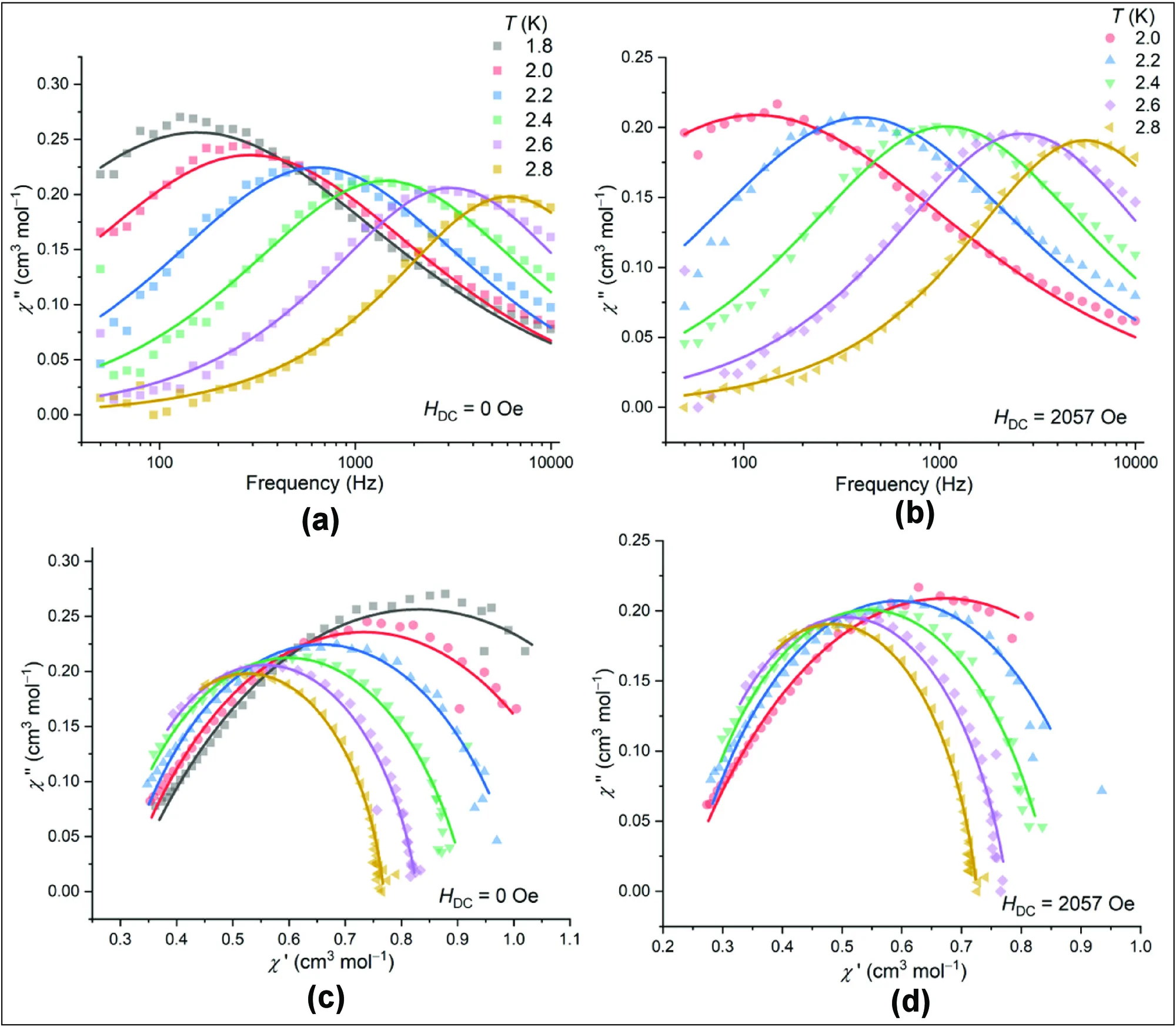
Selected χ″ vs frequency and Cole–Cole plots for *H*_DC_ = 0 Oe (a and b) and *H*_DC_ = 2057 Oe (c and d) measured for complex **1**. Filled symbols represent the experimental values, and the solid lines fits according to the generalized Debye function (see eq S2). A compilation of these fits for all DC fields and temperatures is shown in Figure S14.

As discussed in more detail below, the application of a DC field of 2057 Oe causes the relaxation times τ to decrease significantly compared to the times obtained at zero field. The AC susceptibility data were analyzed using a generalized Debye model (see eq S2) to extract relaxation times τ together with parameter α that describes the distribution of relaxation times. The fitting software CC-FIT2 ^57^ was employed to determine τ with statistically robust uncertainties by properly accounting for the distribution of relaxation times α, thereby yielding reliable error estimates for the derived relaxation parameters.

Obtained relaxation times τ, isothermal and adiabatic susceptibilities *χT* and *χS*, respectively, and relaxation time distribution parameter α are summarized along with their estimated uncertainties in Table S16. At 1.8 K and *H*_DC_ = 0 Oe, α reaches a relatively large value of 0.42(1), indicative of a broad distribution of relaxation times and correspondingly large uncertainties (error bars in Figure 9). At 2.8 K (also at zero DC field), α decreases significantly to 0.13(1), consistent with a narrower relaxation time distribution and reduced uncertainties in the extracted τ values. The dependence of relaxation times τ, obtained over a wide temperature range, and magnetic DC fields were first evaluated as plots versus temperature, τ­(*T*), for each applied field consecutively. Two different models were considered to describe the temperature dependence of τ. In the first approach, the data were fitted using a combination of an Orbach process and a quantum tunneling of magnetization (QTM) term

2 $$\tau^{-1}\left(T\right)={\tau_{0}}^{-1}\;\exp\left({-U}_{\mathrm{eff}}/T\right)+{\tau_{Q\mathrm{TM}}}^{-1}$$

with Orbach relaxation prefactor τ_0_^–1^, effective energy barrier *U*_eff_, and quantum tunneling rate τ_QTM_^–1^. Even though a direct term is recommended to substitute for the QTM term for DC fields higher than 5 kOe,^57^ the QTM term is kept here at 8 kOe for the sake of consistency. Figure 9a exemplarily shows the corresponding fit according to eq 2 to the relaxation times obtained at zero DC field. A compilation of the corresponding fits to all AC measurements can be found in Figure S15, and the extracted parameters from the fit are summarized in Table S17. Figure 9c provides an overview of the τ versus temperature fits for a selection of DC fields (for the sake of clarity, the uncertainties of the relaxation rates are not plotted here). Exemplarily, at zero field the parameters τ_0_ = 10^–8(3)^ s, *U*_eff_ = 25(20) K, and τ_QTM_ = 10^–3(1)^ s have been determined. The uncertainties of the determined relaxation parameters are comparably large here due to the broad inherent distribution of relaxation times (large α parameters especially for the low-temperature regions) that have adequately been considered by the applied fitting routine.^57^ Assuming a single relaxation time by hypothetically considering only the average values without their uncertainty, as done in many publications, would return relaxation parameters with much smaller uncertainties of course, which would however not correctly represent the true inherent nature of the system with a very broad distribution of relaxation times. Nevertheless, the obtained parameters should be used with caution in any comparison, discussion, or interpretation, due to the very large values of uncertainty arising from the broad distributions of relaxation times. Even though there are good agreements of the fits according to eq 2 with the experimental data, an alternative model consisting of a Raman instead of an Orbach relaxation process has also been used for fitting the τ versus temperature curves:

3 $$\tau^{-1}\left(T\right)=CT^{n}+{\tau_{\mathrm{QTM}}}^{-1}$$

with Raman prefactor *C* and Raman exponential temperature factor *n*. A compilation of the fits according to eq 3 to all τ versus temperature curves is presented in Figure S16 with the extracted fit parameters summarized in Table S18. Exemplarily, at zero field, the parameters *C* = 10^0(3)^ s K^*–n*^, *n* = 10(7), and τ_QTM_ = 10^–3(3)^ s were determined.

**Figure 9:**
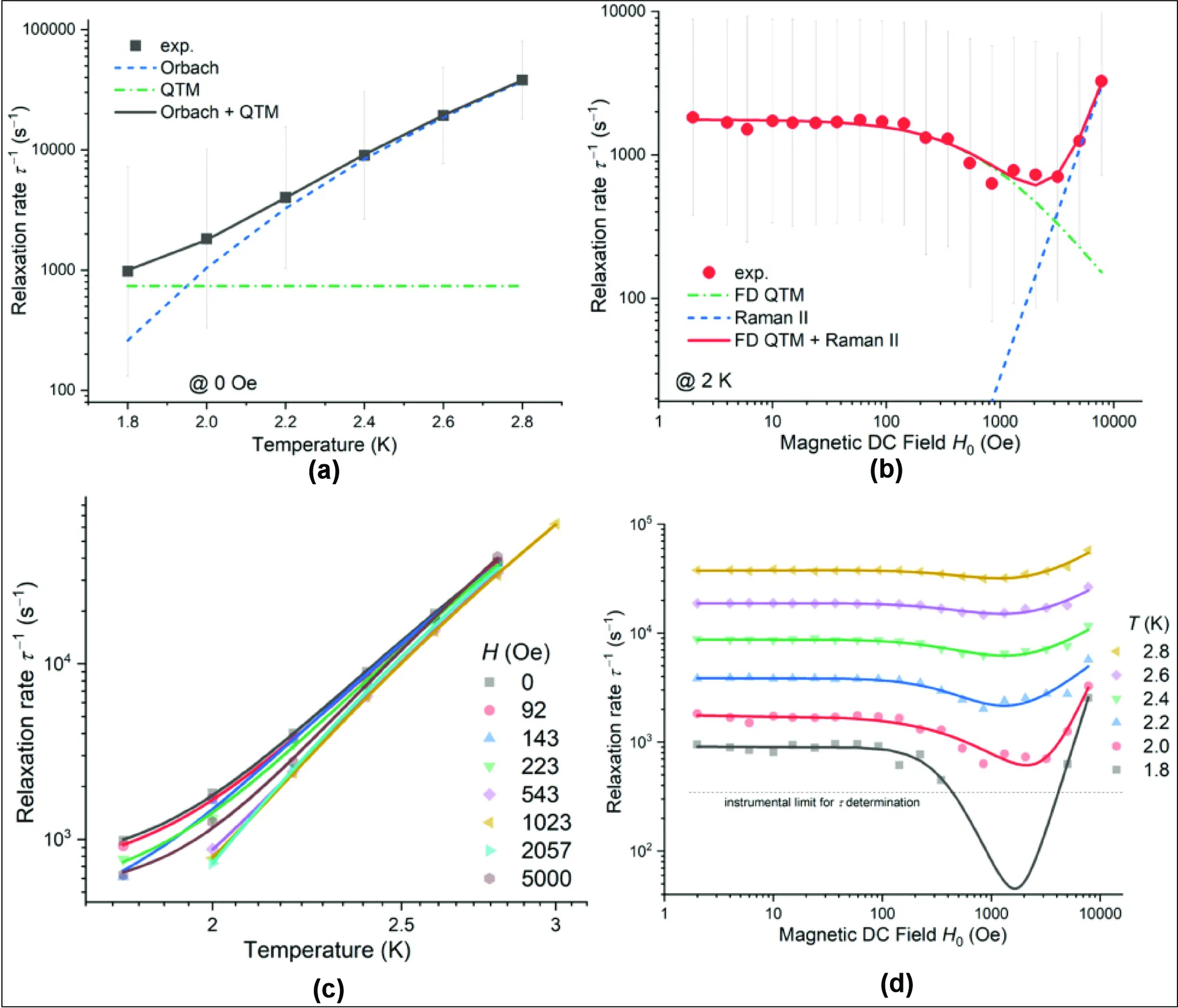
(a) Experimental relaxation rates τ^–1^ vs temperature (filled symbols) exemplarily shown for zero DC field and fit with a combination of Orbach and QTM relaxation processes (lines). (b) Experimental relaxation rates τ^–1^ vs magnetic DC field (filled symbols) exemplarily shown at 2 K and a fit with a combination of field-dependent (FD) QTM and Raman II relaxation processes (the constant term is not shown here). (c) Relaxation rates τ^–1^ vs temperature for selected magnetic DC fields together with a fit consisting of Orbach and QTM. (d) Relaxation rates τ^–1^ vs field (*H*_DC_) for selected temperatures together with a fit consisting of a FD QTM, a Raman II relaxation process, and a constant term. The uncertainties of inverse relaxation rates τ^–1^ (error bars in panels a and b) have been omitted from panels c and d for the sake of clarity.

A Raman exponential factor *n* close to 9 aligns with expectations for a Kramers ion well below the Debye temperature (noting the large parameter uncertainties). Since the relaxation times for 1 have only been determined up to maximum temperatures of about 3.5 K, where only few phonons are available, a Raman relaxation process involving virtual excited states might rather be realized than a pure Orbach relaxation process at these low temperatures. It should be noted that the relaxation times quickly increase with temperature and cross the instrumental limit (maximum AC excitation frequency) at a relatively low temperature of only about 3.5 K (depending on the applied DC field). In the comparably small available temperature region, discrimination between a Raman and an Orbach relaxation process is not possible. Since the experimental data are equally well described by either assuming a Raman or an Orbach relaxation process, a further fit including both a Raman and an Orbach relaxation next to the QTM has been omitted to avoid overparametrization.

In addition to the fits of the τ versus temperature curves discussed above, the τ versus DC field curves were also fitted according to the following expression

4 $$\tau^{-1}\left(H\right)=\frac{{\tau_{\mathrm{QTM}}}^{-1}}{1+{\tau_{\mathrm{QTM}\left(H\right)}}^{-1}H^{p}}+C_{\mathrm{II}}H^{m}+C_{t}$$

incorporating field-dependent QTM parameter τ_QTM(H)_, Raman II prefactor *C*_II_, and constant term *C*_t_. Figure 9b exemplarily shows the corresponding fit according to eq 4 to the field-dependent relaxation times obtained at 2 K. A compilation of the corresponding fits to all measurements is presented in Figure S17 with the extracted fitting parameters summarized in Table S19. Figure 9d provides an overview of the τ versus field fits for all measured temperatures (uncertainties of the relaxation rates have been omitted for the sake of clarity). Most importantly, the τ^–1^ versus field fits reveal a minimum in the relaxation rates close to 2 kOe. This minimum arises from a decreasing trend of the relaxation rates with an increase in magnetic field, due to the field-dependent (FD) QTM on one hand and an increasing trend due to the Raman II relaxation process that dominates at higher fields on the other. At 1.8 K, the relaxation rates decrease below the instrumental limit for τ determination around 2 kOe (below an AC excitation frequency of *f* = 50 Hz or ω = 314 s^–1^, the AC signal is too noisy for τ extraction). Ignoring the large uncertainties of the relaxation parameters determined for the τ^–1^ versus field curve at 1.8 K and just considering the average values, a minimum relaxation rate of about 45 s^–1^ located at about 1650 Oe is predicted. In practice, the smallest actually measured relaxation rate of about 450 s^–1^ occurs at 2 K and a DC field of 348 Oe. The minimum of relaxation rates at around 2 kOe is also evident from the τ^–1^ versus temperature curves at different DC fields. As shown in Figure 9c, relaxation rates τ^–1^ change little for small DC fields up to 223 Oe. However, the τ^–1^ versus temperature curves at 543, 1023, and 2057 Oe then exhibit significantly smaller relaxation rates, while the curve measured at 5000 Oe shows higher relaxation rates (at a certain temperature) akin to those at low fields.

Following the observation of a pronounced open hysteresis at 2 K around 35 kOe (Figure 7), a detailed investigation was carried out using DC magnetometry. The sample was initially cooled to 1.8 K under an applied field of 70 kOe. Subsequently, the field was reduced to 35 kOe at the maximum available sweep rate of 217 Oe s^–1^, and the evolution of magnetic moment *m*_*z*_ was recorded over a period of 1 h, as presented in Figure 10. The resulting DC relaxation curves were analyzed by fitting them with a stretched exponential function

5 $$M_{\left(t\right)}=M_{\mathrm{eq}}+\left(M_{0}-M_{\mathrm{eq}}\right)e^{\left(-t/\tau \right)\beta }$$

with *M*_eq_ as the equilibrium magnetization, *M*_0_ as the starting magnetization, and β as the stretch parameter. At 1.8 K, the obtained relaxation time τ of 451(3) s confirms the slow magnetic relaxation for complex **1** at 35 kOe that has already been indicated by the hysteresis effects of the field scans.

**Figure 10:**
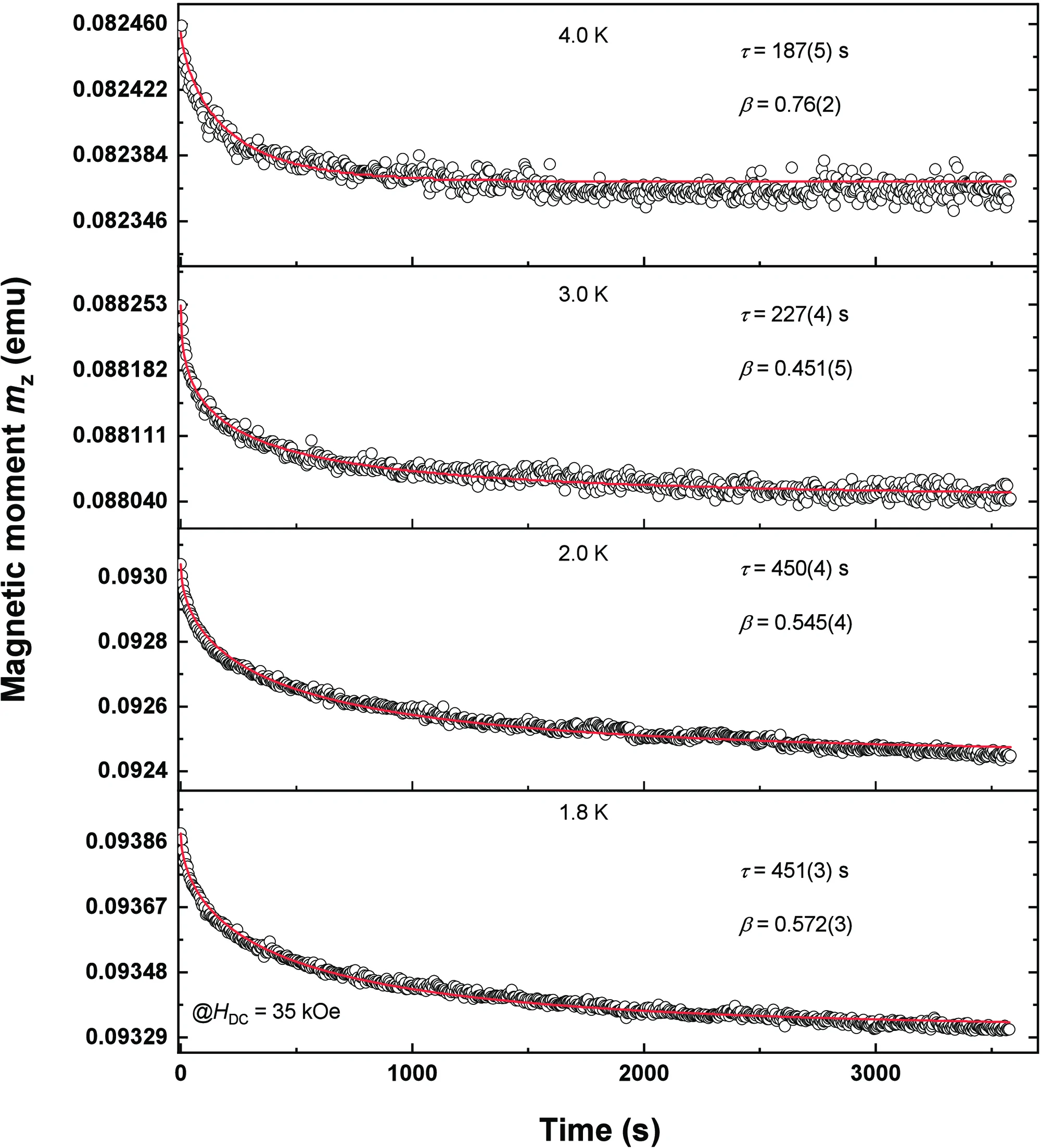
Time-dependent evolution of the measured magnetic moment (with c.g.s. “unit” emu) once the field has been reduced from 70 to 35 kOe with a maximum available rate of 215 Oe s^–1^ (empty symbols). The red lines represent fits with a stretched exponential, and the corresponding relaxation times τ and stretch parameters β are listed.

The large stretch parameter β of 0.572(2) points to a very broad distribution of relaxation times. With an increase in temperature, the relaxation times progressively decrease, reaching τ = 187(5) s at 4 K. At higher temperatures, reliable extraction of relaxation times is no longer feasible, as the relative variation of magnetic moment over time becomes increasingly negligible. Specifically, the relative change in magnetic moment is approximately −0.6% at 1.8 K but decreases to only about −0.1% at 4 K.

### Theoretical Calculations

To rationalize the observed magnetic behavior and elucidate the underlying electronic structure, ab initio electronic structure calculations were performed, which have become an important tool for understanding magnetic anisotropy and relaxation mechanisms in molecular magnetic materials.^58–60^ All quantum mechanical calculations were carried out using the ORCA 5.0.4 program package.^61^ Each Co­(II) center was treated independently through a diamagnetic-substitution approach, where the other two Co­(II) ions were replaced by a closed-shell Zn­(II) ion. The two terminal tetrahedral Co1 sites are related by crystallographic inversion symmetry through the central Co2 ion; a single calculation therefore describes both terminal ions. SA-CASSCF calculations with CAS­(7,10) active space were carried out for both Co­(II) site types, and NEVPT2 with extended CAS­(13,13) active space was used for the terminal Co(1) ions to capture the sulfur covalency.^62^ Spin–orbit coupling was treated via the QDPT approach, and the SINGLE_ANISO and POLY_ANISO modules were used to compute the blocking barrier, Zeeman splitting, and magnetic susceptibility.^63^ BS-DFT calculations with the B3LYP and ωB97X functionals were used for exchange coupling.^64,65^ Full computational details, including basis sets, active space compositions, model truncation (Figure S12), auxiliary fitting, and all roots, are provided in the Supporting Information.

In the linear Co1–Co2–Co1 trimer, adjacent exchange pathways are described by *J* and the terminal–terminal pathway is described by *J*′. To isolate these interactions via diamagnetic substitution, *J* was obtained from calculations in which one terminal Co1 was replaced by Zn­(II), while *J*′ was extracted by replacing the central Co2. The coupling constants were evaluated using Yamaguchi’s spin-projection approach for the exchange Hamiltonian (eq S1). The calculated values (Table S7) indicate weak antiferromagnetic interactions between the adjacent Co sites and very weak antiferromagnetic coupling between the terminal Co­(II) ions. The magnitude of *J* ranges from −3.48 to −1.68 cm^–1^, and that of *J*′ from −1.15 to −0.77 cm^–1^ depending on the functional, consistently indicating weak antiferromagnetic exchange (Table S7). To further verify the exchange coupling constants, NC-SF-TDDFT calculations were performed on the full trinuclear complex using Q-Chem 6.4.0.^66^ In this approach, a single spin-flip excitation from the high-spin decet (*S* = 9/2) reference generates three target *S* = 7/2 states, from which all pairwise exchange constants are extracted via the effective Hamiltonian approach of Mayhall and Head-Gordon,^67^ as implemented in the ezMagnet postprocessing.^68^ Unlike BS-DFT, this approach treats the full trinuclear system without diamagnetic substitution and accesses the multiconfigurational low-spin states within a single-reference formalism. Two functionals were employed with the def2-TZVP basis set. B3LYP yielded *J* = −3.82 cm^–1^ and *J*′ = −0.78 cm^–1^, while LRC-wPBEh gave *J* = −2.63 cm^–1^ and *J*′ = −0.39 cm^–1^. These values, obtained without fitting to experimental data, are consistent with the BS-DFT, confirming the weak antiferromagnetic exchange coupling.

The central Co2 site possesses a ^4^T_1_ ground term with first-order orbital angular momentum, while the terminal Co(1) sites have ^4^A_2_ ground terms with ideally quenched orbital momentum. However, the low symmetry of the terminal coordination environment (two Cl atoms, one S atom, and one I atom) restores a significant orbital contribution, consistent with the increased *χT* value at 300 K. QDPT-SOC produces 120 SOC states as Kramers doublets, providing complete spectra of single-ion magnetic anisotropy. For the octahedral Co2 center, six KDs fall within ∼1666 cm^–1^, arising from the SOC-split ^4^ *T*_1_ ground multiplet; the ground to first-excited KD gap is 146 cm^–1^ (NEVPT2) (Table S9). The tetrahedral sites, in comparison, show only two thermally accessible KDs arose from the ^4^ *A*_2_ ground term, separated by ∼45 cm^–1^ (NEVPT2). The substantial energy gap between KD2 and KD3 (>2400 cm^–1^) confirms the quenching of first-order orbital angular momentum at terminal sites relative to the octahedral middle site (Table S13 and Figure S13). In addition, the soft sulfur and heavy iodide donors introduce noticeable covalency, affecting SOC mixing, which contributes to the magnetic anisotropy for terminal ions. As a result, the magnetic anisotropy of the central Co2 site is substantially larger, reflecting its dominant first-order orbital contribution. The *D* and *g* parameters for each Co­(II) site are listed in Tables 1 and 2.

**Table 1:** Computed ZFS Parameters (effective Hamiltonian approach) (*D*, *E*/*D*, *E*, and excitation energies for the Co­(II) sites in complex **1**)

site	method	*D* (cm^–1^)	*E*/*D*	*E* (cm^–1^)	Δ*E*_1_ ^a^	Δ*E*_1_ ^b^
Co2)	CASSCF	–76.62	0.267	–20.46	–	–
	NEVPT2	–66.81	0.252	–16.84	1091	145.8
Co1	CASSCF	–23.96	0.220	–5.27	–	–
	NEVPT2	–20.46	0.262	–5.36	2425	44.9

a First spin-free (NEVPT2) quartet excitation energy (cm^–1^).

b KD1–KD2 energy gap after spin–orbit coupling (cm^–1^).

**Table 2:** NEVPT2 **g** Tensor Principal Values for the Ground (KD1) and First Excited (KD2) Kramers Doublets of Complex **1**

site	representation	*g_x_*	*g_y_*	*g_z_*	*g*_iso_ ^a^
Co2	KD1 (*S̃* = 1/2)	1.324	1.783	8.144	3.75
	KD2 (*S̃* = 1/2)	2.475	2.985	5.258	3.57
Co1	KD1 (*S̃* = 1/2)	1.371	1.861	7.038	3.42
	KD2 (*S̃* = 1/2)	2.073	2.762	5.771	3.54

a *g*_iso_ = (*g*_*x*_ + *g*_*y*_ + *g*_*z*_)/3.

The NEVPT2 spin-free quartet state energies for the Co1 and Co2 sites are summarized in Table S12. The computed **g** tensors for all three Co­(II) sites exhibit strong axial character (*g*_*z*_ ≫ *g*_⊥_). As shown in Figure 11, the orientations of the principal *g*_*z*_ components are nearly parallel to one another across the trinuclear unit.

**Figure 11:**
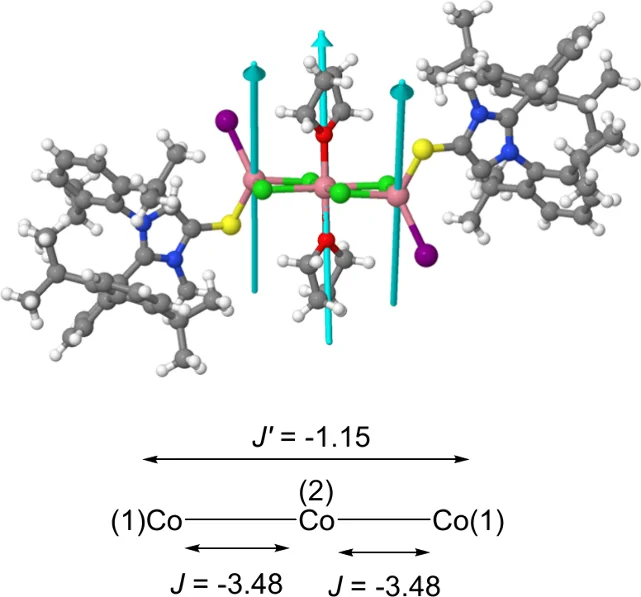
Orientation of *g*_*z*_ components of each Co site in the complete X-ray structure of **1** (top). Spin model and Co­(II)–Co­(II) coupling constants (bottom).

The large negative values of the *D* parameters for Co2 and Co1, e.g., −76.30 and −17.97 cm^–1^, respectively (SA-CASSCF­(7,5)), can be explained based on contributions from second-order SOC contributions (Table S8) and AILFT calculation performed on top of SA-CASSCF­(7,5). The AILFT analysis of the Co2 center yields five ligand field orbitals with >96% single d orbital character. For the distorted tetrahedral Co1 sites, the lower site symmetry produces more strongly mixed AILFT eigenfunctions. The excitation of electrons within the same |*m*_L_| values contributes negatively and within different |*m*_L_| values contributes positively to the *D* value. For Co2 and Co1, the largest negative contribution for *D* comes from the first excited spin-free quartet state where the first electronic transition is from d_*xz*_ to d_*yz*_ for Co1 and from d_*xy*_ to d_*x* ^2^–*y* ^2^_ for Co2 having the same |*m*_L_| value, which contributes negatively to *D* with values of −140.663 cm^–1^ (Table S10) and −56.704 cm^–1^ (Table S11), respectively, and the second electronic transition involves different |*m*_L_| values that contribute positively to *D* with values of 26.40 and 19.482 cm^–1^, respectively (as shown in Figure S11). The significantly larger excitation energies at the tetrahedral sites (1865 cm^–1^ vs 784 cm^–1^) directly account for the smaller |*D*| of Co1 relative to Co2, consistent with the inverse energy dependence of the second-order perturbation expression

To calculate the exchange coupling constant beyond BS-DFT treatment and account for the strong single-ion anisotropy of each Co­(II) site, Lines’ formalism within the POLY_ANISO module implemented in ORCA was used. This semi-ab initio approach takes the full spectrum of SOC states and wave functions computed independently for each type of Co­(II) site at the CASSCF/NEVPT2+SOC level and constructs the total magnetic Hamiltonian for the Co_3_-linear trinuclear complex by combining the on-site SOC contributions with isotropic exchange coupling and magnetic dipole–dipole interactions between the Co­(II) sites. The exchange spectrum is obtained by diagonalizing this Hamiltonian in the product basis of single-ion Kramers doublets, and the resulting exchange-coupled states are used to compute powder magnetization *M*(*H*, *T*) and magnetic susceptibility *χT*(*T*). Isotropic Lines exchange parameters *J* and *J*′ are the only adjustable parameters and were optimized against experimental *χT*(*T*) and *M*(*H*, *T*) data. The best-fit parameters are *J* = −3.39 cm^–1^ and *J*′ = −1.15 cm^–1^ (Figure 12), confirming the weak antiferromagnetic exchange indicated by BS-DFT calculations.

**Figure 12:**
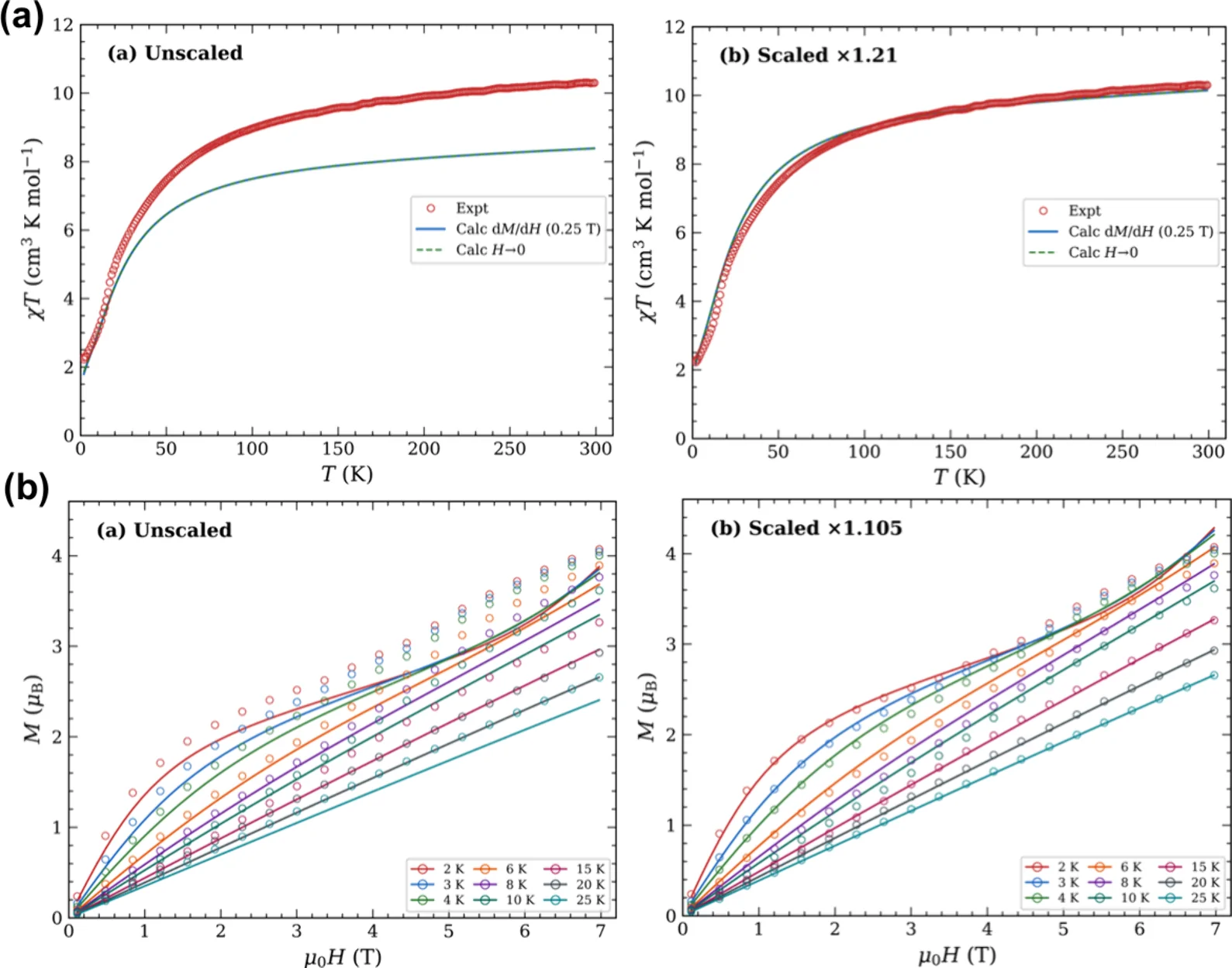
(a) *χT*(*T*) vs *T* for the Co_3_ complex: (empty circles) experimental data, (solid blue line) calculated d*M*/d*H* at 0.25 T, and (dashed green line) zero-field limit (scaled by a factor of 1.21). (b) Magnetization curves *M*(*H*) at 2–25 K: (empty symbols) experimental data and (solid lines) calculated data (scaled by a factor of 1.105). Low-temperature bending at 3.5–5.5 T reflects AFM to FM ground state crossover.

A central feature of the POLY_ANISO approach is that the on-site magnetic anisotropy of each Co­(II) center, namely, the **g** tensors, **D** tensors, and the full set of single-ion magnetic moments, is entirely determined by the CASSCF/NEVPT2+SOC wave functions computed for each individual site. Crucially, these on-site properties are not adjustable parameters in the Lines model; only isotropic exchange constants *J* and *J*′ can be fitted. This is fundamentally different from the phenomenological spin Hamiltonian treatment (eq 1), where *D*, *g*_⊥_, *g*_∥_, and *J* are all treated as free parameters. For polynuclear systems, the fixed on-site anisotropy makes the POLY_ANISO model inherently less flexible. Any systematic error in the computed single-ion properties propagates directly into the calculated magnetic observables without the possibility of correction through parameter adjustment.^33^

In the present case, the main source of such a systematic error is the NEVPT2 treatment of dynamic electron correlation. NEVPT2, as a second-order perturbation method, tends to overestimate d–d excitation energies. Since SOC mixes excited states into the ground state via second-order contributions, overestimated excitation gaps reduce the effective SOC mixing and consequently lead to underestimated *g*_*z*_ values and on-site magnetic moments. This effect is particularly relevant for the octahedral Co2 site, where the first-order orbital angular momentum contribution from the ^4^T_1_ ground multiplet is sensitive to the d–d gap. A further limitation arises from the active space. For the central Co2 site, a rigorous treatment of the bridging ligand contributions would require inclusion of the four chloro-bridged 3p orbitals along with the d orbitals of all three Co­(II) centers, leading to a CAS­(31,22) active space that is computationally prohibitive with current resources. The CAS­(7,10) space used here for Co2, therefore, does not account for the covalent contributions from the bridging chloride ligands to the same extent as the CAS­(13,13) space captures the sulfur covalency at the terminal sites.

Because of these fixed on-site anisotropy limitations, satisfactory reproduction of the experimental data required uniform scaling of the computed magnetic quantities by α_M_ = 1.105 for *M*(*H*, *T*) and α_*χT*_ = 1.21 for *χT*(*T*). These scaling factors effectively compensate for the underestimated on-site magnetic moments without altering the fitted exchange parameters or the relative energy spectrum of the exchange-coupled states. After scaling, excellent agreement with experiment is obtained (Figure 12), validating both the single-ion electronic structure and exchange coupling constants derived for complex **1**. The low-temperature bending observed in the *M*(*H*) curves at 3.5–5.5 T reflects the AFM to FM ground state crossover, as captured by the POLY_ANISO calculation.

The exchange spectrum of complex **1**, corresponding to fitted parameters *J* and *J*′, is shown in Figure 13. The physical picture of the magnetization reversal barrier is that the system starts in the D1^–^ state (lower left; *M*_Z_ = −3.55 μ_B_), must somehow traverse the barrier, and arrives in the D1^+^ state (lower right; *M*_Z_ = 3.55 μ_B_) to complete magnetization reversal. The barrier structure reveals two different reversal pathways. The classical barrier extends from D1 at 0 cm^–1^ to D4 at 23.4 cm^–1^, representing the energy that would be required for purely Orbach relaxation via sequential same-side excitation D1^–^ → D2^–^ → D3^–^ → D4^–^, tunnelling through D4 (QTM transition magnetic moment (TMM) = 0.083), and de-excitation D4^+^ → D3^+^ → D2^+^ → D1^+^. Above D4 at 23.4 cm^–1^, the diagram shows D5 at 45.9 cm^–1^, which arises from the inclusion of excited single-ion Kramers doublets in the exchange calculation. D5 has a notably small *M*_Z_ of only 0.699 μ_B_ and a large QTM TMM of 0.419 μ_B_.

**Figure 13:**
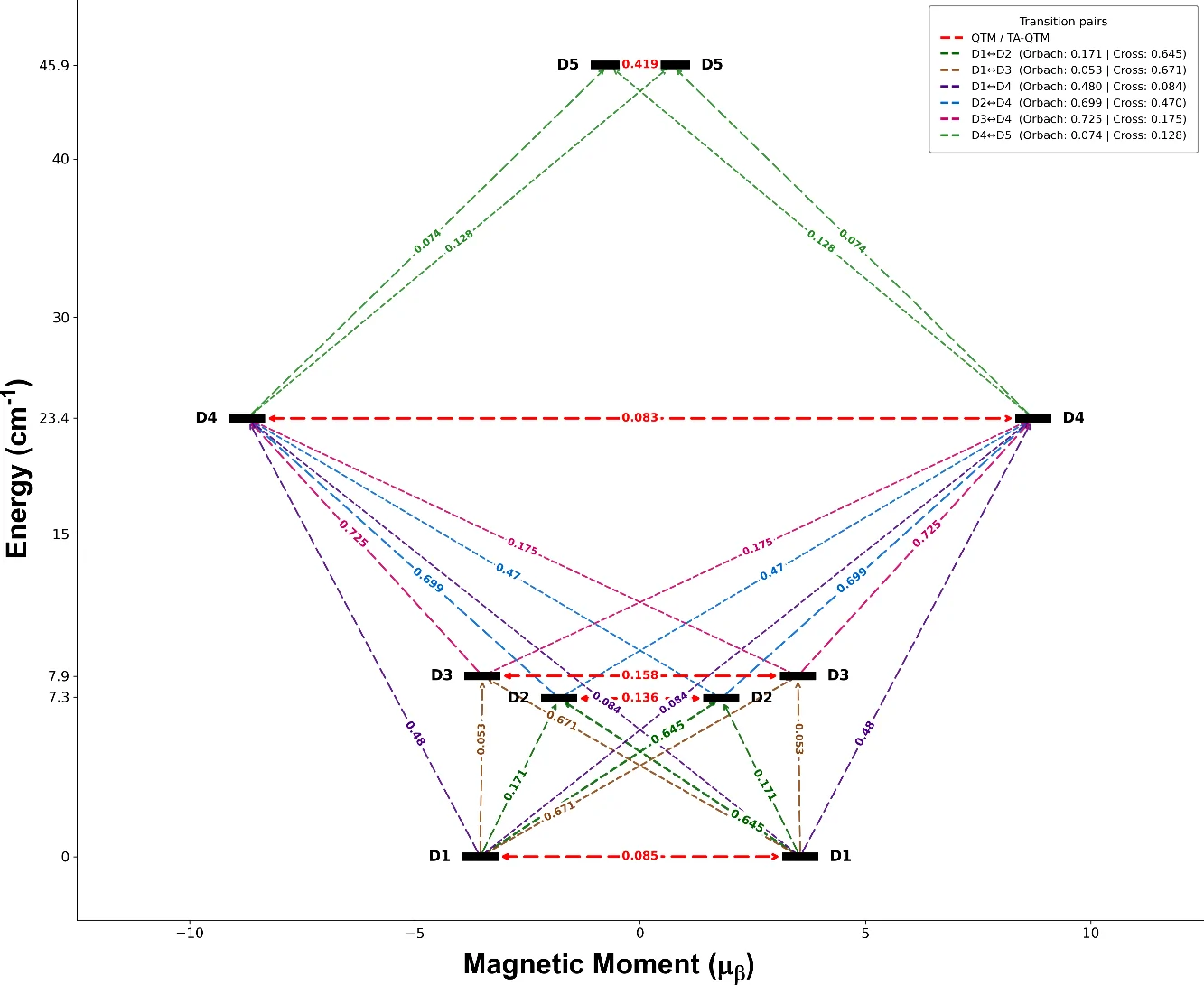
Magnetization reversal barrier diagram for the trinuclear Co­(II) SMM, showing exchange-coupled doublets D1–D5 plotted as energy vs magnetic moment *M*_Z_. Black horizontal bars represent doublet energy levels on both the “–” (left) and “+” (right) branches. Red dashed horizontal arrows indicate QTM/TA-QTM pathways with TMM values. Colored dashed arrows show Orbach (same-side) and cross-relaxation (opposite-side) transition pathways, with TMM (transition magnetic moment) values annotating each arrow. On the left (D^–^) side, arrows point upward, indicating thermal excitation; on the right (D^+^) side, arrows point downward, indicating relaxation. The color coding distinguishes different transition pathways.

The small same-side TMM for the D4 → D5 step (0.074 μ_B_) and moderate cross-side TMM (0.128 μ_B_) indicate that D5 does not participate significantly in the relaxation dynamics of the low-energy D1–D4 manifold. The classical barrier height of ∼33 K (D4 at 23.4 cm^–1^) represents the energy required for a purely Orbach relaxation through D4. However, the actual barrier experienced by the system is much lower, because the large cross-relaxation TMMs between D1 and D2/D3 provide shortcut pathways that bypass D4 entirely. The molecule reaches the D2/D3 level through phonon-assisted excitation with *U*_eff_ ≈ 7–8 cm^–1^ (∼10–12 K) and then waits at these excited levels for the slow interwell tunnelling or cross-relaxation event, terminating the effective relaxation at D2/D3. The large cross-relaxation TMMs between D1 and D2/D3 (0.645 μ_B_ and 0.660 μ_B_) provide efficient shortcut pathways to bypass D4 entirely. The experimental *U*_eff_ of 25(20) K from the AC susceptibility analysis (eq 2) has a large standard deviation, and both the classical barrier of ∼33 K and the effective barrier of ∼10–12 K fall within this uncertainty range. We note that the transition magnetic moment analysis is primarily qualitative, as no universal critical value has been established for distinguishing efficient from inefficient transitions

As the trinuclear Co­(II) complex with three d^7^ centers constitutes a Kramers system, the exchange-coupled electronic states can be grouped into Kramers doublets (KDs). The method described by Yin and Li^69^ provides a route to the quantitative prediction of the QTM relaxation time from the principal *g* factors of the ground and excited Kramers doublets, with the ab initio *g* factors being the only required computational input. The ground exchange-coupled KD obtained from POLY_ANISO has *g*_*x*_ = 0.219, *g*_*y*_ = 0.289, and *g*_*z*_ = 7.101, giving a transversal component *g*_*xy*_ = 0.363. This relatively large transversal *g* component confirms that the ground doublet does not possess high magnetic axiality, consistent with QTM being an active relaxation pathway at zero DC field. The predicted log_10_(τ_QTM_) = −6.41, compared to the experimentally fitted value of −2.87(1.40) from eq 2. As stated by Yin and Li,^69^ the accuracy target of this method is mainly the order of magnitude. Furthermore, using the same approach, *U*_eff_ was estimated within the framework of thermally activated QTM (TA-QTM). Restricting the calculation to physically accessible KDs 1–4 (KD 5 is kinetically inaccessible based on the TMM analysis in Figure 13), the predicted *U*_eff_^Zee^ – 300 = 6.9 cm^–1^ = 10 K. This value is consistent with the effective barrier of 7–8 cm^–1^ (10–12 K) identified from the TMM-based cross-relaxation analysis via D2/D3 (Figure 13) and falls within the experimental uncertainty range of *U*_eff_ = 25(20) K. The full calculation details are provided in Section SI 16b of the Supporting Information.

The phenomenological spin Hamiltonian (eq 1) uses a single exchange constant *J* = −1.105(5) cm^–1^ and a common axial ZFS parameter *D* = −30.8(3) cm^–1^ for all three Co­(II) sites with effective *S* = 3/2 spins. The ab initio POLY_ANISO treatment, by contrast, distinguishes the two inequivalent site types and yields *J* = −3.39 cm^–1^ and *J*′ = −1.15 cm^–1^ with site-specific *D* values of −66.81 cm^–1^ for octahedral Co2 and −20.46 cm^–1^ for tetrahedral Co1. The numerical differences between the two sets of parameters reflect the different levels of approximation in the two models. The phenomenological approach treats all three sites as equivalent with effective *S* = 3/2 spins and two free parameters, while the ab initio approach resolves the site-specific anisotropy from first-principles and fits only the isotropic exchange. Despite these differences, both approaches consistently indicate weak antiferromagnetic exchange and strong easy-axis anisotropy, and both reproduce the experimental *χT*(*T*) and *M*(*H, T*) data satisfactorily. To directly relate the theoretical results to the experimentally observed magnetic behavior, the fitted Lines model was used to compute the Zeeman splitting of the low-lying exchange-coupled states as a function of applied magnetic field (Figure 14).

**Figure 14:**
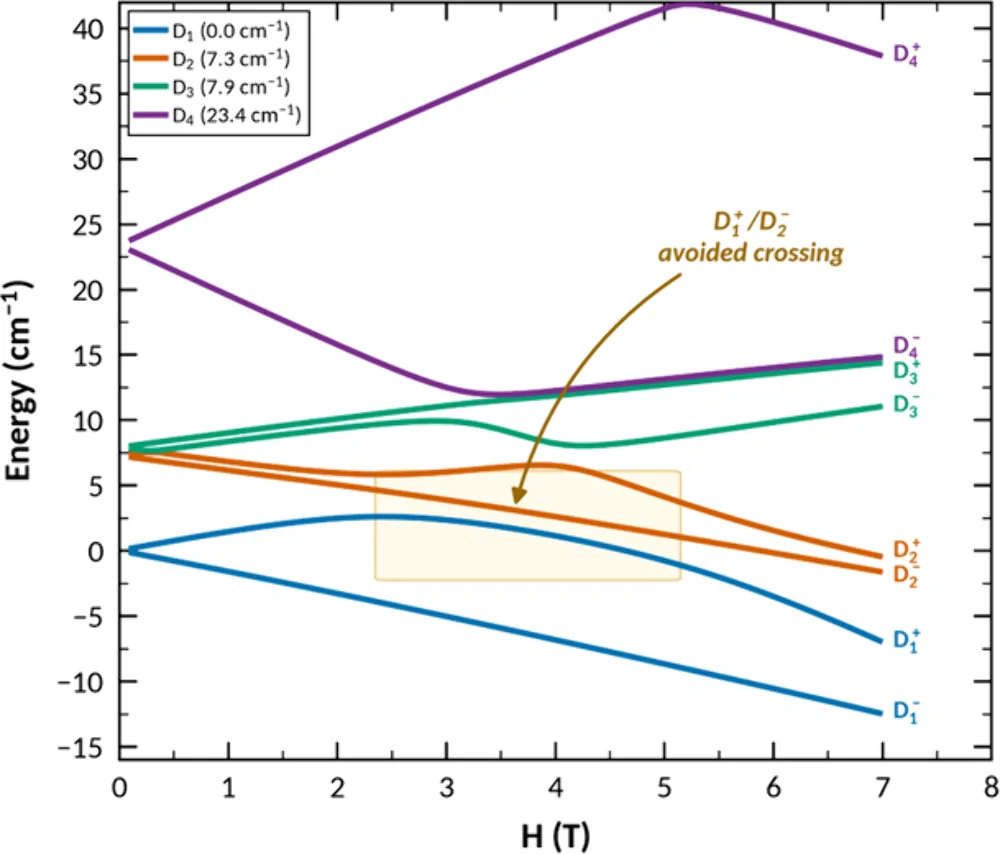
Zeeman splitting of the lowest-lying exchange-coupled states of **1** as a function of magnetic field applied along the (1, 1, 1) direction of the molecular Cartesian frame, computed at the CASSCF/NEVPT2/POLY_ANISO level.

Most importantly, these calculations on a more sophisticated level confirm the level anti-crossing (LAC) character of these states as already discussed for the Zeeman diagram presented in the section on static magnetic properties (Figure 6). The LAC character due to the comparably weak AFM exchange coupling as found for this complex explains some interesting features of the magnetic measurements. Around 35 kOe, where the sweep field scans show an open hysteresis (Figure 7), the D1^+^/D1^–^ gap reaches a maximum of 7.8 cm^–1^, and as a consequence, the QTM is suppressed strongly. Around zero field, the D1^+^/D1^–^ gap is minimal for the Kramers ion and the comparably fast magnetic relaxation mainly via QTM can only be tracked by AC susceptibility that covers higher relaxation rates. As the D1^+^/D1^–^ gap increases and reduces the QTM rate, the D1^+^/D2^−^ gap is reduced simultaneously to only about 1.4 cm^–1^ at 35 kOe. Even though the D1^+^/D2^−^ gap is reduced, the overall Orbach rate with potential D2/D3 cross-relaxation (specifically, Orbach’s relaxation prefactor) represents the limiting factor at 35 kOe that leads to very slow magnetic relaxation and an open hysteresis in the sweep scans. It should be noted that the magnetization reversal barrier diagram together with the TMM for cross-relaxations as shown in Figure 13 could be calculated only at zero magnetic field but was not available for the 35 kOe DC field. As the D1^+^/D2^−^ gap is reduced, the higher states become more mixed into the ground state, and via second-order Van-Vleck susceptibility, the slope of the magnetization versus field curves is changed beyond what would be expected for a purely Brillouin-type behavior (kinks in field sweep curves).

## Conclusion

A linear trinuclear Co­(II) complex (**1**), supported by a sulfur-functionalized zwitterionic ylide-type abnormal N-heterocyclic carbene ligand featuring distinct octahedral and tetrahedral coordination environments, has been synthesized and investigated by dynamic magnetic measurements. Magnetic analyses showed that the increased *χT* value 10.3 cm^3^ K mol^–1^ at 300 K reflects significant orbital contributions from the Co­(II) centers. The experimentally derived coupling constant (*J* = −1.105(5) cm^–1^), supported by *ab initio* values (*J* = −3.39 cm^–1^; *J*′ = −1.15 cm^–1^), together with pronounced axial anisotropy (*D* = −66.81 cm^–1^ for Co2, and *D* ≈ −20.46 cm^–1^ for Co1), places the system in a regime where exchange interactions are comparable to anisotropy effects. Ab initio calculations further reveal that the larger anisotropy at the octahedral Co2 site originates from lower-lying excited states (∼784 cm^–1^), whereas higher excitation energies (∼1865 cm^–1^) at the tetrahedral Co1 sites result in reduced |*D*| values, consistent with second-order spin–orbit coupling contributions. This balance gives rise to level anti-crossing (LAC) of the low-laying Zeeman levels at μ_0_ *H* ≈ 3.5 T, which, in turn, governs the magnetic dynamics. As a consequence, an unusual field-induced slow relaxation process is observed, with open hysteresis between 30 and 45 kOe and long relaxation times (at 1.8 K, τ = 451(3) s and β = 0.572(2)). In contrast, low-field AC susceptibility measurements reveal significantly faster relaxation governed by combined QTM and Orbach processes, leading to *U*_eff_ = 25(20) K, consistent with computed barriers. For comparison, a previously reported ferromagnetically coupled dianionic Co­(II)_3_ “butterfly” complex^33^ exhibits an Orbach effective energy barrier of *U*_eff_ = 94(4) K, most probably because the stronger FM exchange (*J* = 10.2 cm^–1^) even more efficiently suppresses QTM relaxation at low temperatures. A related Co­(II)_3_ complex reported by Zabala-Lekuona, Seco, and Colacio et al.^31^ shows stronger AFM coupling (*J* = −6.38(2) cm^–1^) with a correspondingly smaller effective Orbach energy barrier of *U*_eff_ = 43.8(1) K compared to the complex investigated in this work. Comparison with related Co­(II)_3_ systems highlights the fact that, unlike strong ferromagnetic coupling that suppresses tunneling, weak antiferromagnetic exchange enables LAC-associated relaxation behavior. Importantly, the active space (extended π-orbitals) of the zwitterionic sulfur ylide ligand in **1** plays an important role, controlling the curvature of plots of *M* versus *H* at different temperatures. Overall, this work demonstrates that fine-tuning the balance between exchange coupling and anisotropy is crucial to achieve unusual magnetic relaxation behavior in Co­(II)-based single-molecule magnets.

## Experimental Section

### General Procedures

All of the experiments were performed using either standard Schlenk line techniques under an argon atmosphere or in an inert O_2_-free vacuum employing an inert argon (Ar) gas-filled MBraun Eco Plus glovebox, where O_2_ and H_2_O levels were maintained below 0.1 ppm at all times. All glassware were oven-dried (120 °C) before use. Solvents were distilled under an Ar atmosphere after refluxing with the Na/K alloy for 2 days, followed by vacuum distillation over 4 Å molecular sieves. N-Heterocyclic carbene was synthesized according to the procedures reported in the literature. All of the chemicals and other solvents were purchased from local companies.

### Single-Crystal X-ray Diffraction

X-ray single-crystal diffraction data were collected on a BRUKER VENTURE D8 APEX4 HEED X-ray diffractometer equipped with helios optics monochromated Mo Kα (λ = 0.71073 Å) radiation at 100 K. All data were integrated using SAINT PLUS, and absorption correction was done using the multiscan absorption correction method (SADABS).

### Synthesis of **1**

In a 100 mL Schlenk flask was placed the S-MIC ligand (0.2 mmol, 102 mg). Then, 15 mL of dry THF was added to it, and the mixture was allowed to stir for 5 min, leading to a dark reddish yellow solution. Anhydrous CoCl_2_ (0.4 mmol, 51.9 mg) was added to it, affording a dark green solution. Stirring was continued overnight to ensure the completion of the reaction. The reaction mixture was filtered, and the filtrate was concentrated under vacuum to reach a volume of approximately 3–4 mL followed by the slow addition of 6 mL of *n*-hexane and kept for crystallization. Within a day, green rod-shaped crystals suitable for single-crystal X-ray diffraction were observed to have formed at room temperature. Yield: (60 mg) 20%. IR (KBr, cm^–1^): 3140, 3062, 2964, 2931, 2873, 2659, 2453, 1754, 1626, 1548, 1417, 1260, 1183, 1091, 1067, 1026, 911, 891, 809, 754, 684, 619, 508. UV–visible band (nm): 326 and 679. Mp: 180 °C dec. Elemental analysis (C, H, N) (calcd) (%): C, 54.40 (54.52); H, 6.36 (6.57); N, 2.12 (2.76).

## Supplementary Material



## Notes

Experimental section, spectroscopic data, additional DC and AC magnetic data, ab initio multireference calculations, and additional schemes, figures, tables, and references (PDF)
