# Supplementary material for: Zeeman Level Anti-Crossing and Slow Magnetic Relaxation in an S‑Functionalized Abnormal NHC-Supported Linear CoII 3 Single-Molecule Magnet
Source: Inorg Chem. 2026 Aug 11;65(33):19151–68. doi: 10.1021/acs.inorgchem.6c01997 (PMC13549218; doi:10.1021/acs.inorgchem.6c01997)
Supplement: Supplementary file 1 [file ic6c01997_si_001.pdf]

Zeeman Level Anti-Crossing and Slow Magnetic Relaxation in an S-Functionalized Abnormal NHC-Supported Linear Co^{II}_3 Single-Molecule Magnet

Sangita Mondal,[a] Sunil Kumar,[a] Sujit Das,[a] Liviu Ungur,*[b] Björn Schwarz,*[c] Kartik Chandra Mondal*[a]

[a]Department of Chemistry, Indian Institute of Technology, Madras, Chennai 600036, India.

[b]Office: MD1-05-03A, Department of Chemistry, National University of Singapore, Singapore.

[c]Institute for Applied Materials (IAM), Karlsruhe Institute of Technology (KIT), Hermann-von-Helmholtz-Platz 1, 76344 Eggenstein-Leopoldshafen, Germany.

*E-mail: csdkartik@iitm.ac.in, chmlu@nus.edu.sg, bjoern.schwarz@kit.edu

Supporting Information

Table of Contents

1. General Methods

2. Preparation and Molecular Structure of Complex **1**

3. Crystallographic Details

4. UV-visible Spectrum

5. IR Spectrum

6. Raman Spectrum

7. ESI HRMS Analysis

8. Cyclic Voltammetry

9. XPS Analysis

10. PXRD Analysis

11. TGA Analysis

12. Magnetic and Structural Parameters Comparison of The Reported Co^{II}_3 Complexes Showing SMM Property.

13. Magnetic Measurements

14. Diamagnetic Susceptibility Corrections of Complex **1**

15. Broken-Symmetry Evaluation of The Magnetic Exchange Interactions

16. a. Ab-initio Calculations for Both Types of Co Sites in Complex **1**

b. Prediction of QTM Rate and Effective Barrier from Ground KD *g*-factors

17. AC Magnetic Susceptibility and Relaxation Dynamics Under Variable Field and Temperature

18. DFT Calculation

19. References

1. General Methodes

All the experimentations have been performed using either standard Schlenk line techniques under an argon atmosphere or in an inert O_2-free vacuum employing inert argon (Ar) gas-filled MBraun Eco Plus glove box, where O_2 and H_2O levels were maintained below 0.1 ppm at all times. All glassware were oven-dried (120 °C) before use. Solvents were distilled under Ar atmosphere after refluxing with Na/K alloy for two days, followed by vacuum distillation over 4Å molecular sieves. N-heterocyclic carbene was synthesized according to the procedures reported in the literature. All the chemicals and other solvents were purchased from local companies. UV-Visible absorption spectra were recorded in Jasco V-650 Spectrophotometer using standard quartz cuvettes (1 cm x 1 cm cross-section) in the 200-900 nm range at room temperature in THF solvent. FT-IR spectra were recorded for the solid samples using KBr pellets on a Jasco FT/IR-4100 Spectrometer in the 400-4000 cm^{-1} range. EPR spectra were obtained using an X-band (8.75-9.65 GHz) JEOL Model JES FA200 EPR spectrometer 4.5 K both in solid state. The powder-XRD (PXRD) pattern was examined using Cu Kα (0.154 nm) radiation on a Rigaku Miniflex 600C diffractometer, spanning a range of 2θ values from 5º to 80º. TGA was performed in the temperature range of 30 ^{o}C to 800 ^{o}C on STARe Default DB V18.00: Mettler-Toledo instrument with 1.89 mg of crystalline sample loaded on an Al crucible. Cyclic voltammetry studies were performed on a CH instrument CHI608B (serial no. I0620, 220 V AC 0.4 A). Solid-state Raman spectra was recorded using an alpha300 R Raman microscope.

Single-crystal X-ray diffraction (SC-XRD Analysis)

X-ray single crystal diffraction data were collected on a BRUKER VENTURE D8 APEX4 HEED X-ray diffractometer equipped with helios optics mono-chromated Mo-Kα (λ = 0.71073 Å) radiation at 100 K. All data were integrated using SAINT PLUS, and absorption correction was done using multi-scan absorption correction method (SADABS). Crystallographic data are summarized in Table S1. CCDC **2546380** contains the crystallographic data for this paper.

2. Preparation and Molecular Structure of Complex 1

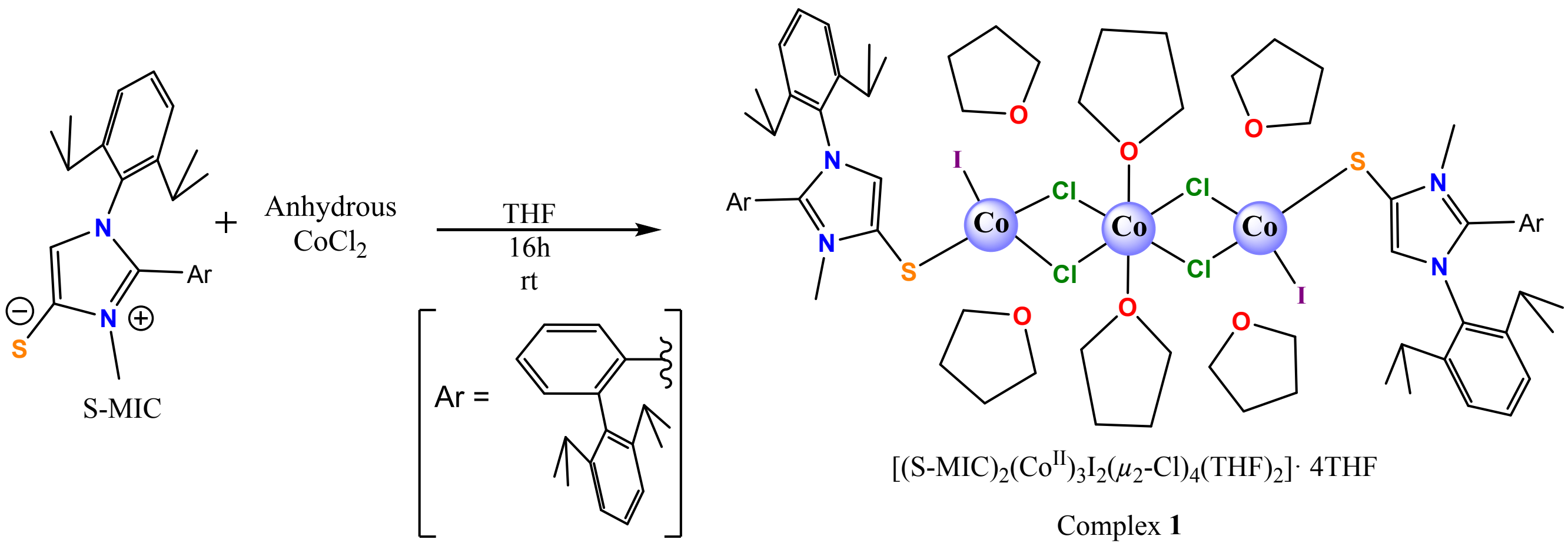

Scheme S1. Synthesis of complex **1**

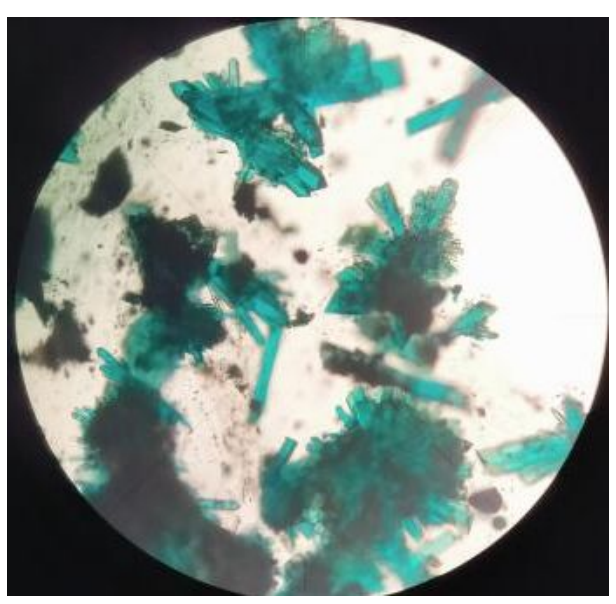

Figure S1. Single crystals picture of complex **1**. All the magnetic measurements are done with single crystals separated manually.

Procedure:

In a 100 mL schlenk flask S-MIC ligand was taken (0.2 mmol, 102 mg). 15 mL of dry THF was added to it and the mixture was allowed to stir for 5 min leading to dark reddish yellow solution. Anhydrous $CoCl_2$ (0.4 mmol, 51.9 mg) was added to it causing a color change to dark green solution. The stirring was continued overnight to ensure the completion of the reaction. The reaction mixture was filtered and the filtrate was concentrated under vacuum to reach a volume of approximately 3-4 mL followed by a slow addition of 6 mL of *n*-hexane and kept for crystallization. Within a day, green rod-shaped crystals were observed to have formed at room temperature suitable for single X-ray single-crystal diffraction. Yield: (60 mg) 20%. IR (KBr, cm^{-1}): 3140, 3062, 2964, 2931, 2873, 2659, 2453, 1754, 1626, 1548, 1417, 1260, 1183, 1091, 1067, 1026, 911, 891, 809, 754, 684, 619, 508. UV-visible band (nm): 326 and 679. M.P. 180 ^{o}C (dec). Elemental analysis (C, H, N) (Calc.) (%): C, 54.40 (54.52); H, 6.36 (6.57); N, 2.12 (2.76).

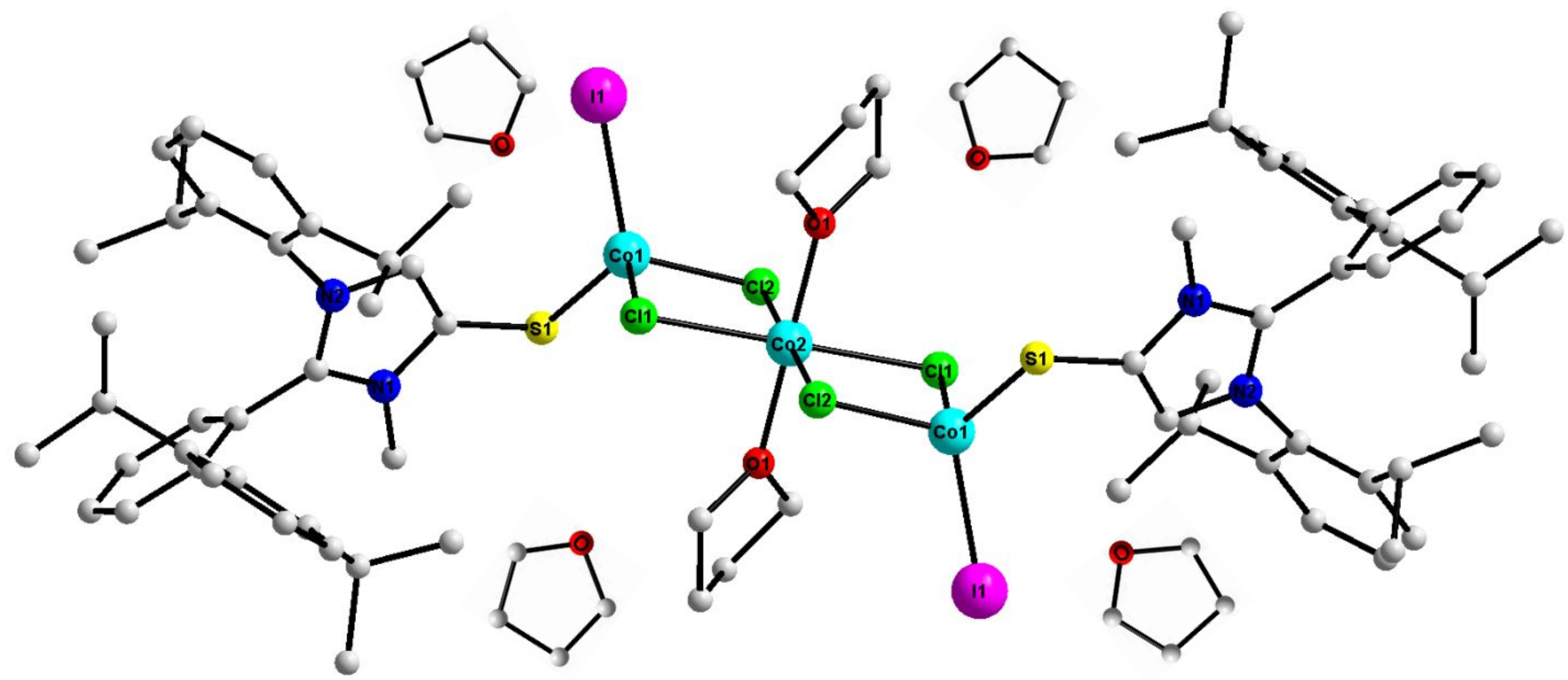

Figure S2. Molecular structure of complex $[(S\text{-}MIC)_2(Co^{II})_3I_2(\mu_2\text{-}Cl)_4(THF)_2]\cdot 4THF$ (**1**). H atoms are omitted for clarity. Colours: Co, sky blue; S, yellow; Cl, green; I, pink; O, red; N, deep blue; C, grey. Selected bond lengths (Å) and bond angles (°): Co2—Cl1 2.4839 (12), Co2—Cl2 2.4841 (11), Co2—O1 2.049 (4), Co1—Cl1 2.2948 (14), Co1—Cl2 2.3120 (14), Co1—S1 2.2938 (14), I1—Co1 2.5724 (8), S1—Co1—I1 116.85 (4), Co1—Cl1—Co2 86.63 (4), Co1—Cl2—Co2 86.26 (4), O1—Co2—Cl1 90.19 (11), O1—Co2—Cl2 91.13 (10).

3. Crystallographic Details

Table S1. Summary of crystallographic parameters and the refinement details of complex **1**

Complex	**1**
Empirical formula	$C_{92}H_{132}Cl_4Co_3I_2N_4O_6S_2$
Formula weight	2026.52
Crystal size [mm]	0.14 × 0.12 × 0.12
Wavelength [Å]	0.71073
Crystal system	Monoclinic
Space group	$P2_1/n$
a [Å]	14.1952 (8)
b [Å]	18.3263 (10)
c [Å]	19.1605 (10)
α [°]	90
β [°]	104.105 (2)
γ [°]	90
V [$Å^3$]	4834.2 (5)
Z	2
Temperature [K]	120
r [Mg m^{-3}]	1.392
m [mm^{-1}]	1.35
F (000)	2094
θ-area [°]	2.0–25.0
Reflections collected	84718
Independent reflections	8539
Reflections with I> 2σ(I)	6655
R_{int}	0.085
Number of restraints	90
Parameters	575
*R*1 [*I*>2*s*(*I*)]	0.052
*wR*2[*I*>2*s*(*I*)]	0.1337
*R*1 [all data]	0.0697
*wR*2 [all data]	0.147
GooF	1.07
Largest diff. peak / hole / e $Å^{-3}$	0.86 / −1.31
CCDC number	**2546380**

Table S2. All bond lengths (Å) of complex **1**.

I1—Co1	2.5724 (8)	C8—C17	1.489 (6)
Co1—S1	2.2938 (14)	C9—C10	1.379 (7)
Co1—Cl1	2.2948 (14)	C10—C11	1.389 (7)
Co1—Cl2	2.3120 (14)	C11—C12	1.383 (7)
Co2—O1^{i}	2.049 (4)	C13—C14	1.521 (8)
Co2—O1	2.049 (4)	C13—C20	1.532 (8)
Co2—Cl1^{i}	2.4838 (12)	C15—C21	1.522 (8)
Co2—Cl1	2.4839 (12)	C15—C16	1.536 (8)
Co2—Cl2	2.4841 (11)	C18—C19	1.350 (7)
Co2—Cl2^{i}	2.4841 (11)	C23—C24	1.402 (7)
S1—C18	1.732 (5)	C23—C28	1.413 (7)
O1—C38	1.460 (7)	C24—C25	1.392 (7)
O1—C35	1.461 (6)	C24—C29	1.511 (7)
O2—C39	1.418 (13)	C25—C26	1.379 (8)
O2—C42	1.433 (17)	C26—C27	1.381 (7)
O3—C45	1.16 (3)	C27—C28	1.388 (7)
O3—C46	1.51 (3)	C28—C31	1.511 (7)
O3'—C46'	1.28 (4)	C29—C33	1.544 (7)
N1—C17	1.342 (6)	C29—C30	1.546 (7)
N1—C18	1.395 (6)	C31—C32	1.519 (8)
N1—C22	1.476 (6)	C31—C34	1.548 (9)
N2—C17	1.350 (6)	C35—C36	1.502 (8)
N2—C19	1.387 (6)	C36—C37	1.530 (8)
N2—C23	1.453 (6)	C37—C38	1.500 (8)
C1—C6	1.411 (7)	C39—C40'	1.39 (3)
C1—C2	1.414 (7)	C39—C40	1.60 (3)
C1—C7	1.501 (6)	C40—C41	1.53 (3)
C2—C3	1.392 (7)	C40'—C41	1.49 (3)
C2—C13	1.526 (7)	C41—C42	1.430 (18)

C3—C4	1.383 (8)	C44—C47	1.37 (2)
C4—C5	1.369 (8)	C44—C45	1.57 (2)
C5—C6	1.396 (7)	C46—C47	1.27 (2)
C6—C15	1.522 (7)	C44'—C47'	1.28 (5)
C7—C12	1.393 (7)	C44'—C45'	1.46 (6)
C7—C8	1.411 (6)	C46'—C47'	1.40 (4)
C8—C9	1.399 (6)		

Symmetry code: (^{i}) -*x*+2, -*y*+1, -*z*+1.

Table S3. All bond angles (°) of complex **1**.

S1—Co1—Cl1	113.98 (6)	C10—C9—C8	121.6 (5)
S1—Co1—Cl2	102.42 (5)	C9—C10—C11	118.7 (4)
Cl1—Co1—Cl2	97.91 (5)	C12—C11—C10	119.8 (5)
S1—Co1—I1	116.85 (4)	C11—C12—C7	123.1 (5)
Cl1—Co1—I1	110.25 (4)	C14—C13—C2	111.9 (5)
Cl2—Co1—I1	113.64 (4)	C14—C13—C20	108.8 (5)
O1^{i}—Co2—O1	180.0	C2—C13—C20	111.3 (4)
O1^{i}—Co2—Cl1^{i}	90.20 (11)	C6—C15—C21	113.2 (5)
O1—Co2—Cl1^{i}	89.81 (11)	C6—C15—C16	110.0 (5)
O1^{i}—Co2—Cl1	89.80 (11)	C21—C15—C16	110.9 (5)
O1—Co2—Cl1	90.19 (11)	N1—C17—N2	106.8 (4)
Cl1^{i}—Co2—Cl1	180.0	N1—C17—C8	122.6 (4)
O1^{i}—Co2—Cl2	88.88 (10)	N2—C17—C8	129.9 (4)
O1—Co2—Cl2	91.13 (10)	C19—C18—N1	105.3 (4)
Cl1^{i}—Co2—Cl2	91.25 (4)	C19—C18—S1	132.2 (4)
Cl1—Co2—Cl2	88.76 (4)	N1—C18—S1	122.4 (4)
O1^{i}—Co2—Cl2^{i}	91.12 (10)	C18—C19—N2	108.6 (4)
O1—Co2—Cl2^{i}	88.88 (10)	C24—C23—C28	122.8 (4)

Cl1^{i}—Co2—Cl2^{i}	88.75 (4)	C24—C23—N2	117.9 (4)
Cl1—Co2—Cl2^{i}	91.24 (4)	C28—C23—N2	118.7 (4)
Cl2—Co2—Cl2^{i}	180.00 (3)	C25—C24—C23	116.9 (5)
Co1—Cl1—Co2	86.63 (4)	C25—C24—C29	119.1 (4)
Co1—Cl2—Co2	86.26 (4)	C23—C24—C29	124.0 (4)
C18—S1—Co1	101.49 (16)	C26—C25—C24	122.1 (5)
C38—O1—C35	109.3 (4)	C25—C26—C27	119.4 (5)
C38—O1—Co2	125.5 (3)	C26—C27—C28	122.2 (5)
C35—O1—Co2	125.1 (3)	C27—C28—C23	116.6 (4)
C39—O2—C42	102.9 (11)	C27—C28—C31	118.8 (4)
C45—O3—C46	106 (2)	C23—C28—C31	124.6 (4)
C17—N1—C18	110.6 (4)	C24—C29—C33	111.6 (4)
C17—N1—C22	127.4 (4)	C24—C29—C30	110.9 (4)
C18—N1—C22	121.9 (4)	C33—C29—C30	109.3 (4)
C17—N2—C19	108.7 (4)	C28—C31—C32	111.3 (5)
C17—N2—C23	131.0 (4)	C28—C31—C34	111.7 (5)
C19—N2—C23	120.1 (4)	C32—C31—C34	111.0 (5)
C6—C1—C2	119.8 (4)	O1—C35—C36	103.9 (4)
C6—C1—C7	119.6 (4)	C35—C36—C37	101.7 (4)
C2—C1—C7	119.9 (4)	C38—C37—C36	103.0 (5)
C3—C2—C1	119.1 (5)	O1—C38—C37	105.7 (5)
C3—C2—C13	118.1 (4)	C40'—C39—O2	115.1 (14)
C1—C2—C13	122.7 (4)	O2—C39—C40	113.7 (14)
C4—C3—C2	120.8 (5)	C41—C40—C39	91.4 (17)
C5—C4—C3	120.3 (5)	C39—C40'—C41	102.0 (18)
C4—C5—C6	121.3 (5)	C42—C41—C40'	107.5 (14)
C5—C6—C1	118.8 (5)	C42—C41—C40	107.2 (16)
C5—C6—C15	117.8 (4)	C41—C42—O2	107.8 (12)
C1—C6—C15	123.2 (4)	C47—C44—C45	101.7 (15)
C12—C7—C8	116.4 (4)	O3—C45—C44	109 (2)

C12—C7—C1	116.0 (4)	C47—C46—O3	107 (2)
C8—C7—C1	127.4 (4)	C46—C47—C44	109 (2)
C9—C8—C7	120.3 (4)	C47'—C44'—C45'	103 (4)
C9—C8—C17	113.6 (4)	O3'—C46'—C47'	120 (3)
C7—C8—C17	125.9 (4)	C44'—C47'—C46'	115 (3)

Symmetry code: (^{i}) -x+2, -y+1, -z+1.

4. UV-visible Spectrum:

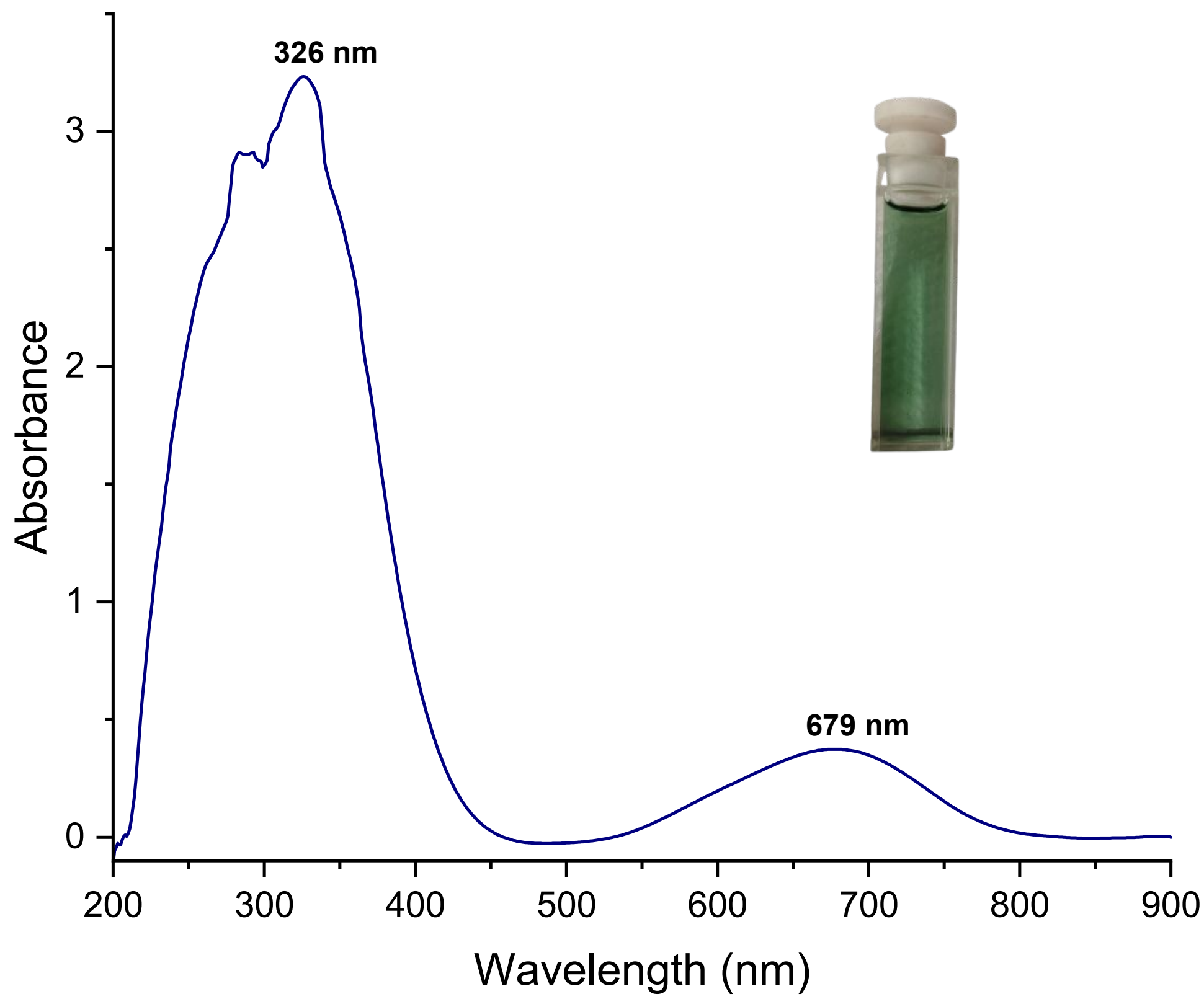

Figure S3. UV-visible spectrum of complex **1** in THF solvent from 200 to 900 nm. G1-Co in THF is **green** coloured [λ_{max} (nm): 326, 679].

5. Infrared Spectrum

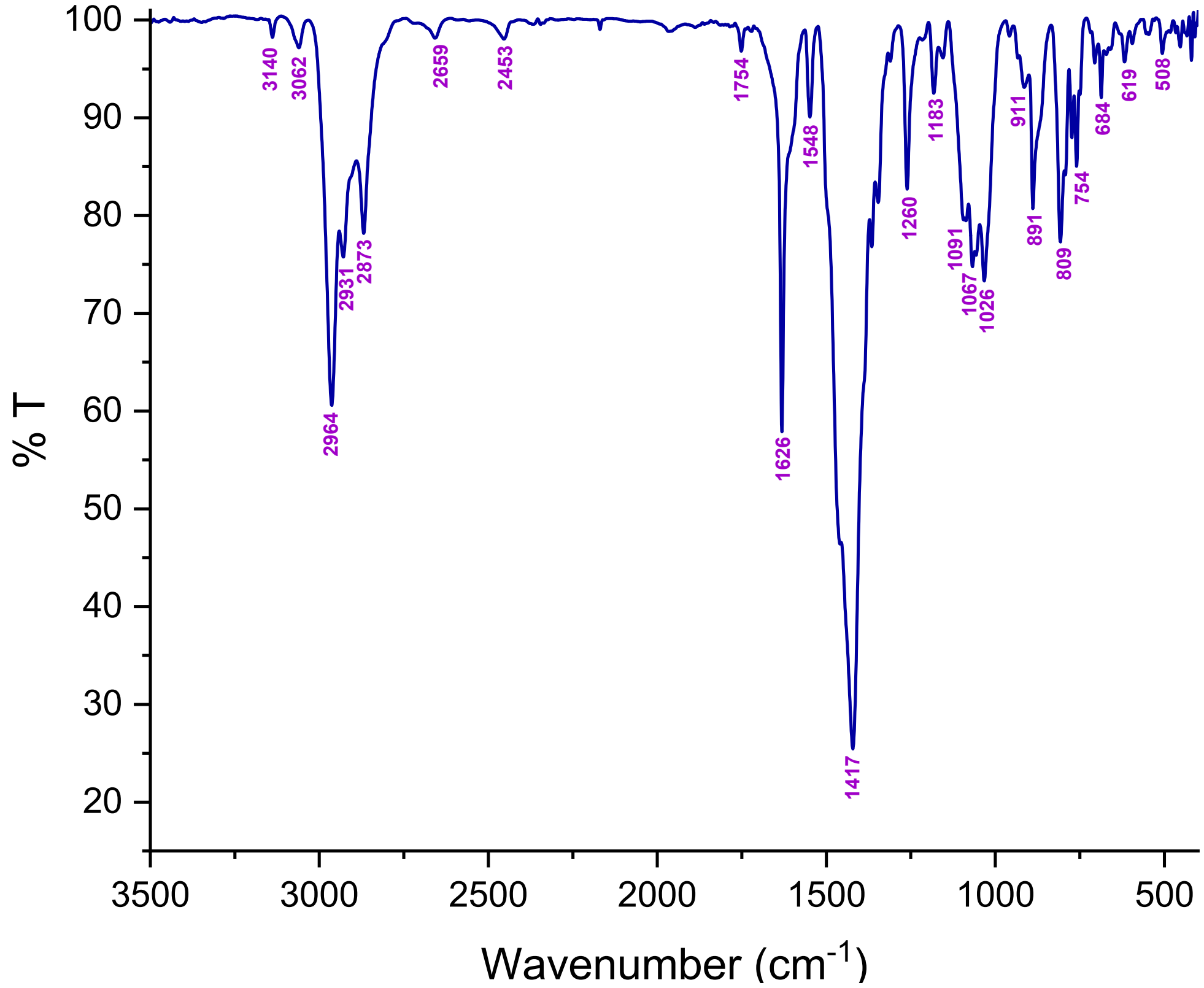

Figure S4. Solid state FT-IR spectrum of complex **1** using KBr pellet. Stretching frequency (υ, cm^{-1}): 3140, 3062, 2964, 2931, 2873, 2659, 2453, 1754, 1626, 1548, 1417, 1260, 1183, 1091, 1067, 1026, 911, 891, 809, 754, 684, 619, 508.

6. Raman Spectrum

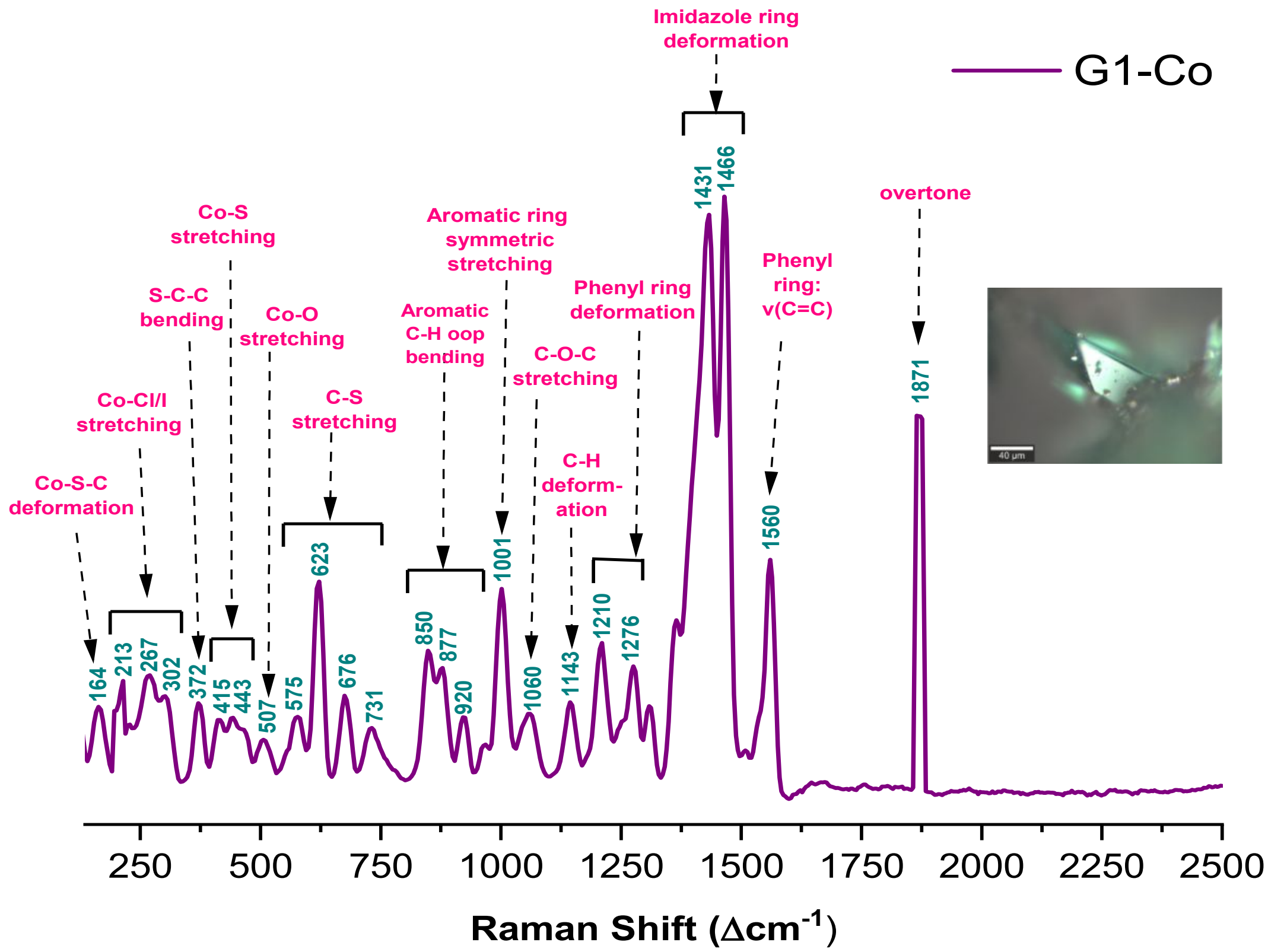

Figure S5. Solid state Raman spectrum of **1** measured in the Alpha300 R Raman Microscope.

Solid-state Raman spectra of complex **1** was recorded using an alpha300 R Raman microscope. During measurement, the most crystalline region was identified and selected for spectral acquisition, as indicated in the corresponding optical micrographs (scale bar: 40 µm).

Table S4. Assignment of Raman bands of **1**

Band (cm^{-1})	Intensity	Assignment of bands	Reference range (cm^{-1})	References
164	Medium	Co-S-C deformation	141	S1
213	Medium	Co-Cl / I stretching	87-255	S1, S2
267	Medium	Co-Cl stretching	87-255	S1, S2
302	Weak (shoulder}	Co-Cl stretching	295-327	S3
372	Medium	S-C-C bending	364	S4
415	Weak	Co-S stretching	412	S5
443	Very weak	Co-S stretching	465	S5
507	Weak	Co-O stretching	500	S5
575	Weak	C-S stretching	570-750	S6
623	Strong	C-S stretching	570-750	S6
676	Medium	C-S stretching	570-750	S6
731	Medium	C-S stretching	570-750	S6
850	Strong	Aromtaic C-H oop bending	790-850	S7
877	weak	Aromtaic C-H oop bending	790-850	S7
920	Medium	Aromtaic C-H oop bending	900	S7
1001	Strong	Aromatic ring symmetric stretching vibration	1001	S8
1060	Medium	C-O-C stretching	1066	S9
1143	Medium	C-H deformation	1151-1163	S10
1210	Strong	Phenyl ring deformation	1200, 1280	S11
1276	Medium	Phenyl ring deformation	1200, 1280	S11
1431	Very strong	Imidazole ring deformation	1431, 1489	S12
1466	Very strong	Imidazole ring deformation	1431, 1489	S12
1560	Strong	Phenyl ring: ν(C=C)	1552–1564	S10
1871	Very strong	Overtone	1870	S13

7. ESI-HRMS Analysis

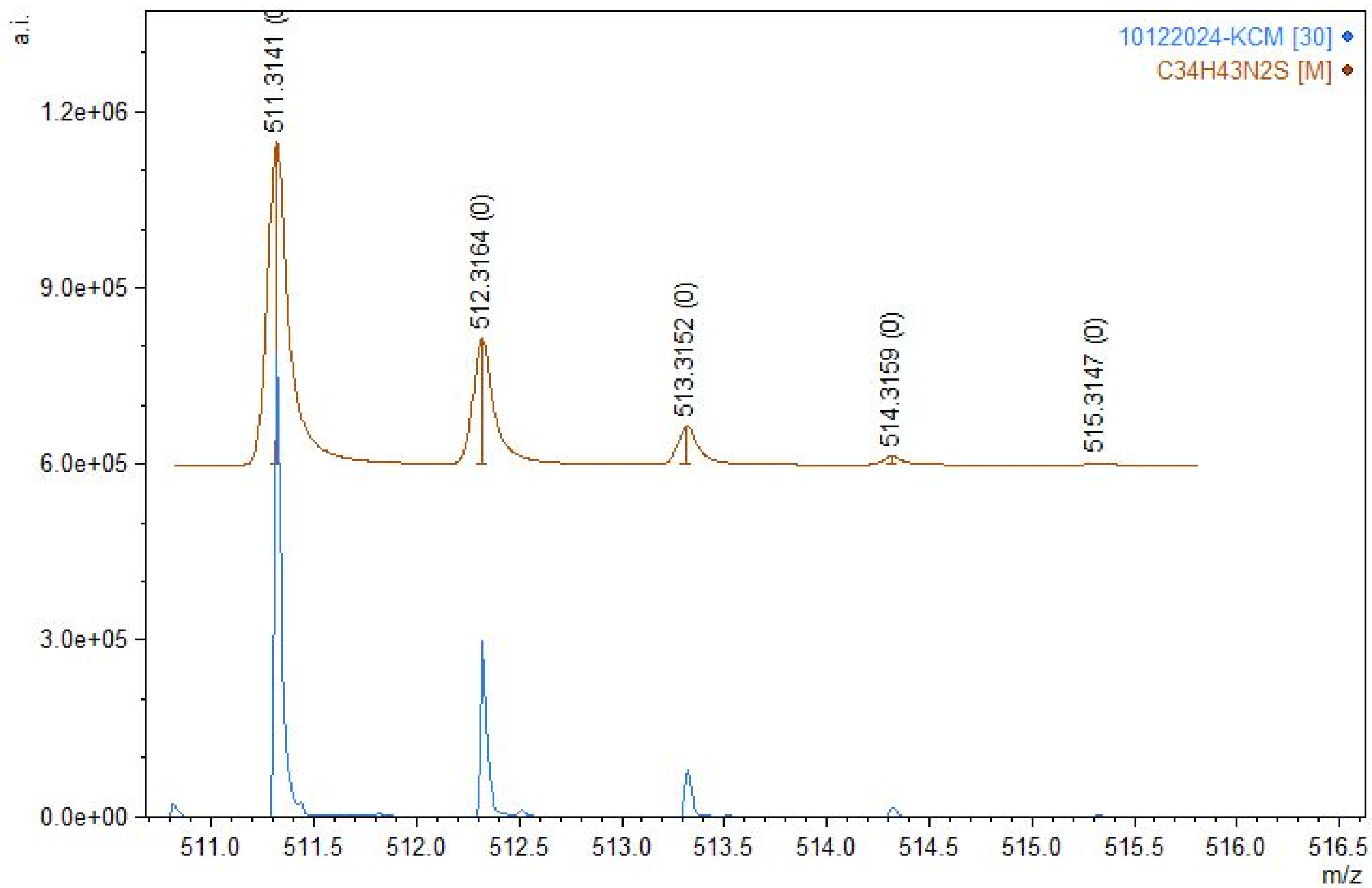

Figure S6. Experimental (blue) and simulated (red) ESI HRMS spectra in positive mode $[M+H]^+$ of **1** in dichloromethane solvent gave fragment peaks for S-MIC ligand (m/z = 510) moiety only.

8. Cyclic Voltammetry

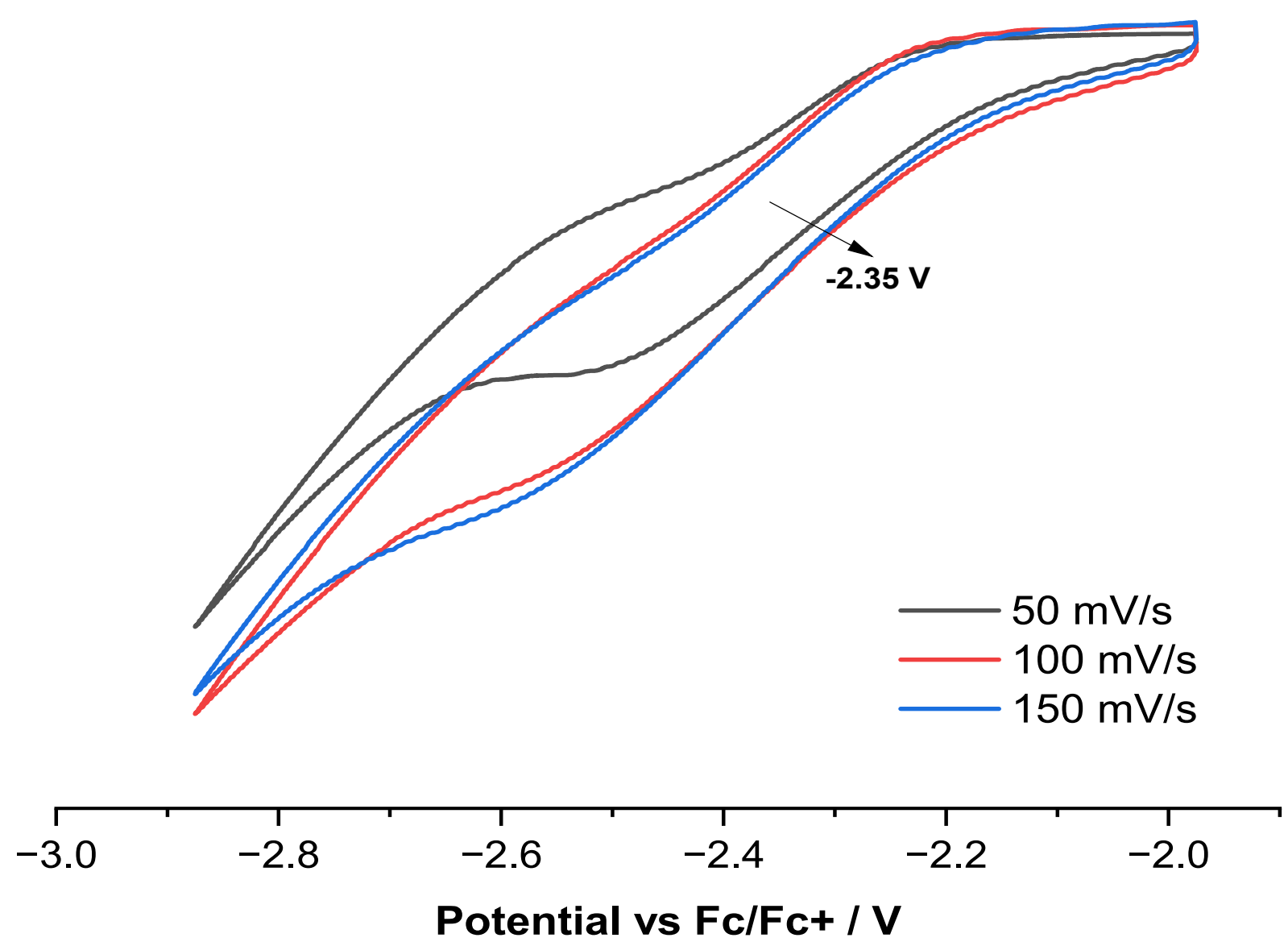

Figure S7. Cyclic voltammogram of complex **1** in THF solution of 0.1 M [*n*-Bu_4N]ClO_4 with RE: Ag, WE: GC, and CE: Pt.

9. X-Ray Photoelectron Spectroscopy (XPS) Analysis

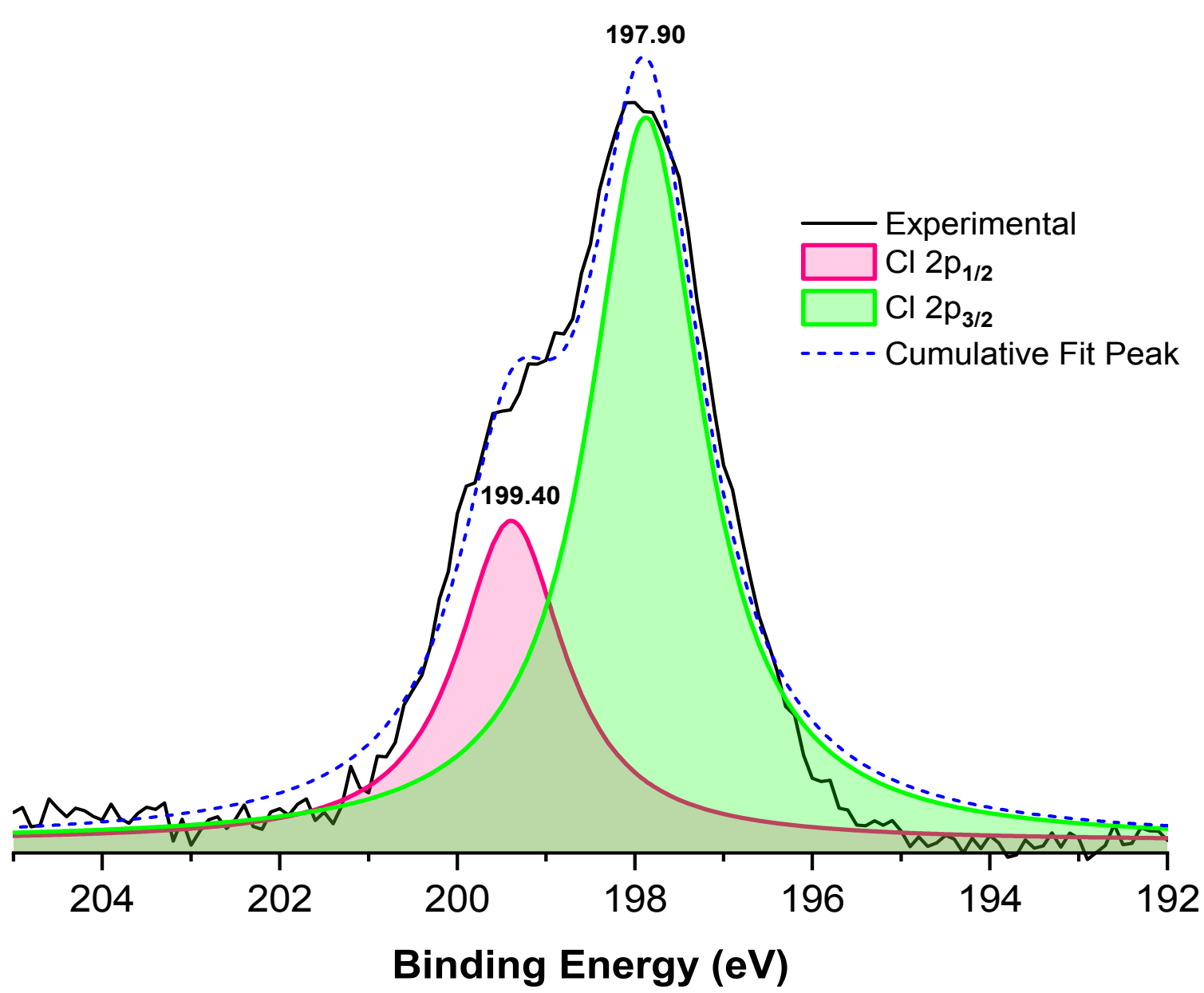

(a)

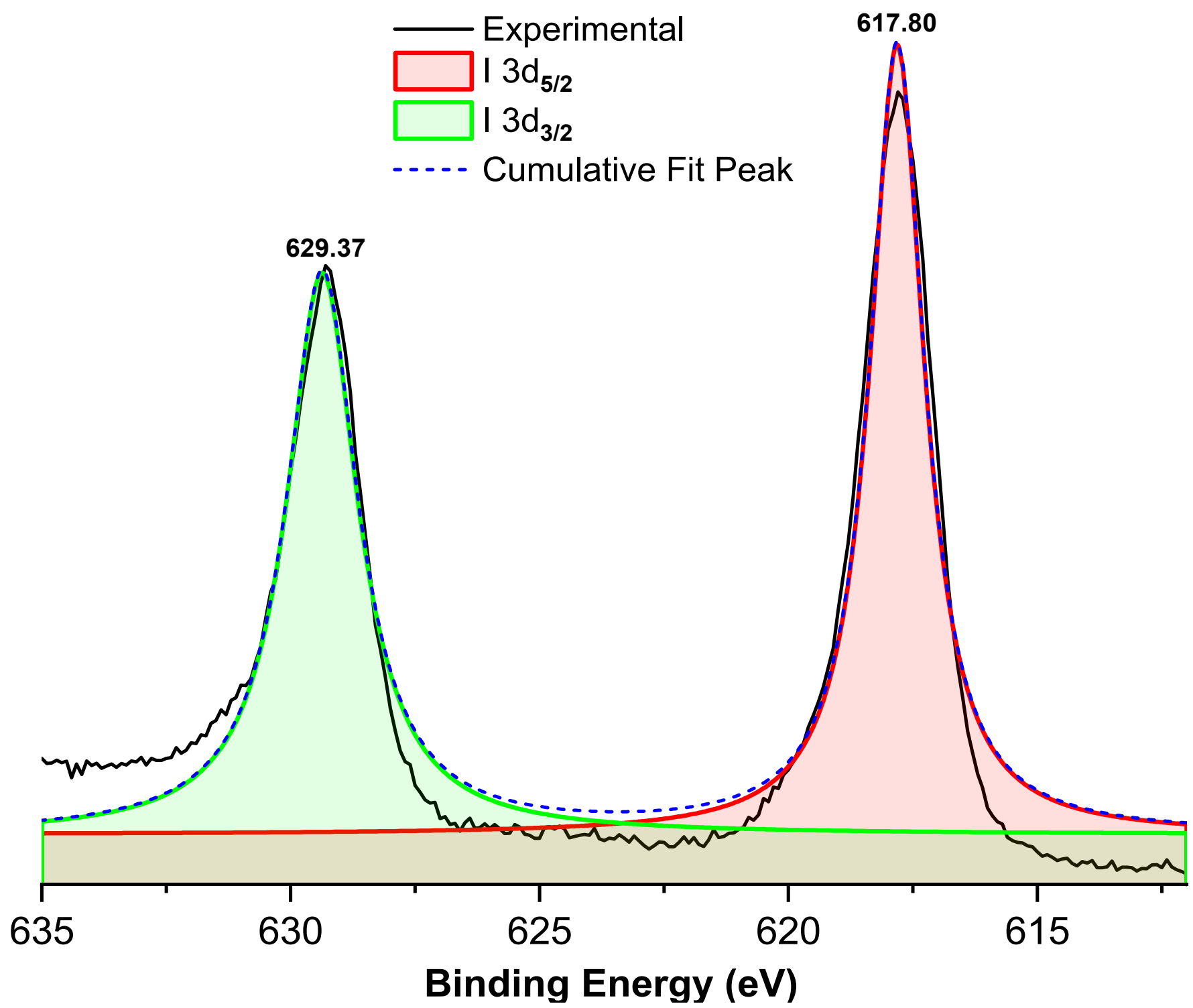

Experimental
I 3d5/2
I 3d3/2
Cumulative Fit Peak
617.80
629.37
635
630
625
620
615
Binding Energy (eV)

(b)

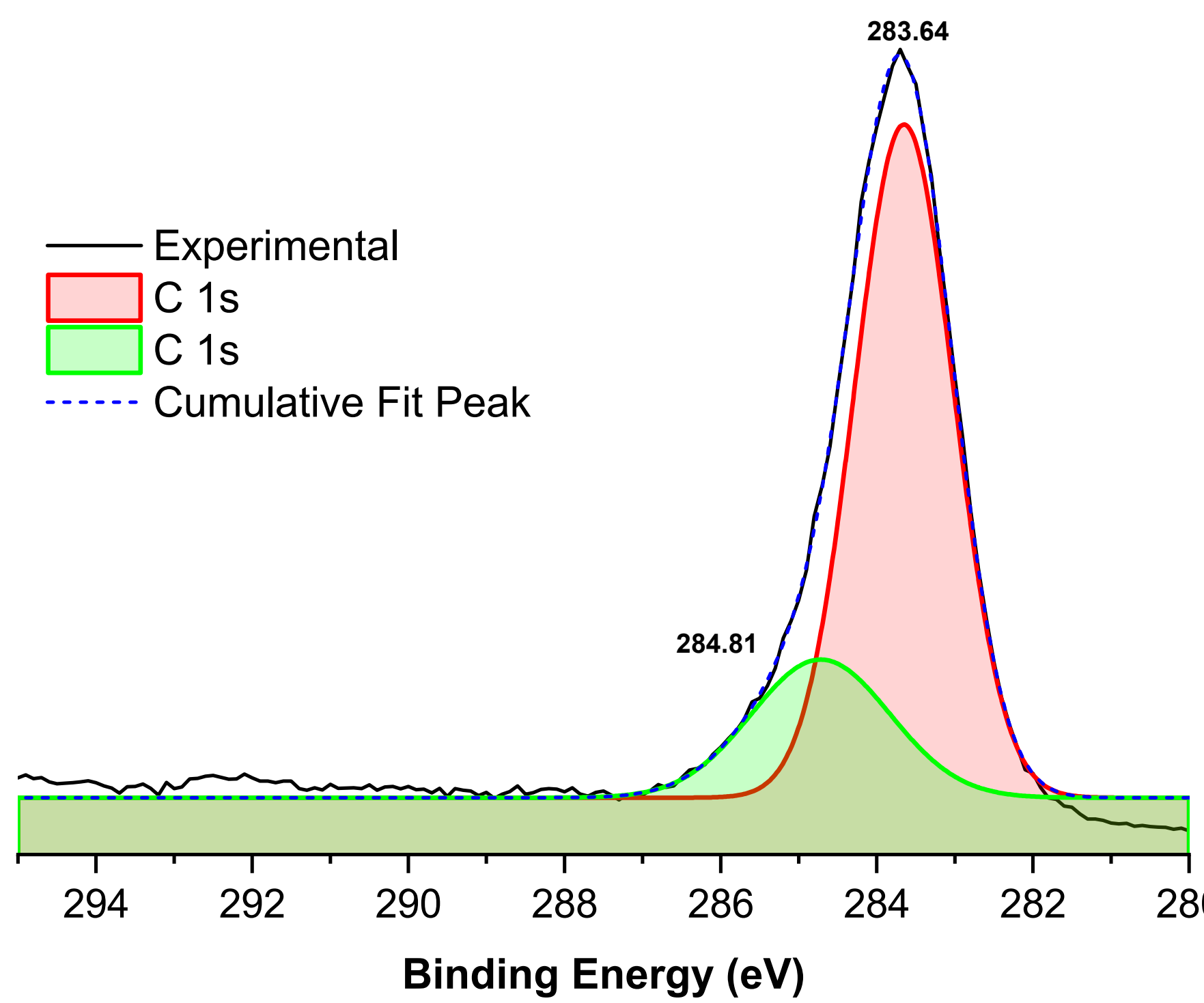

283.64
Experimental
C 1s
C 1s
Cumulative Fit Peak
284.81
294
292
290
288
286
284
282
280
Binding Energy (eV)

(c)

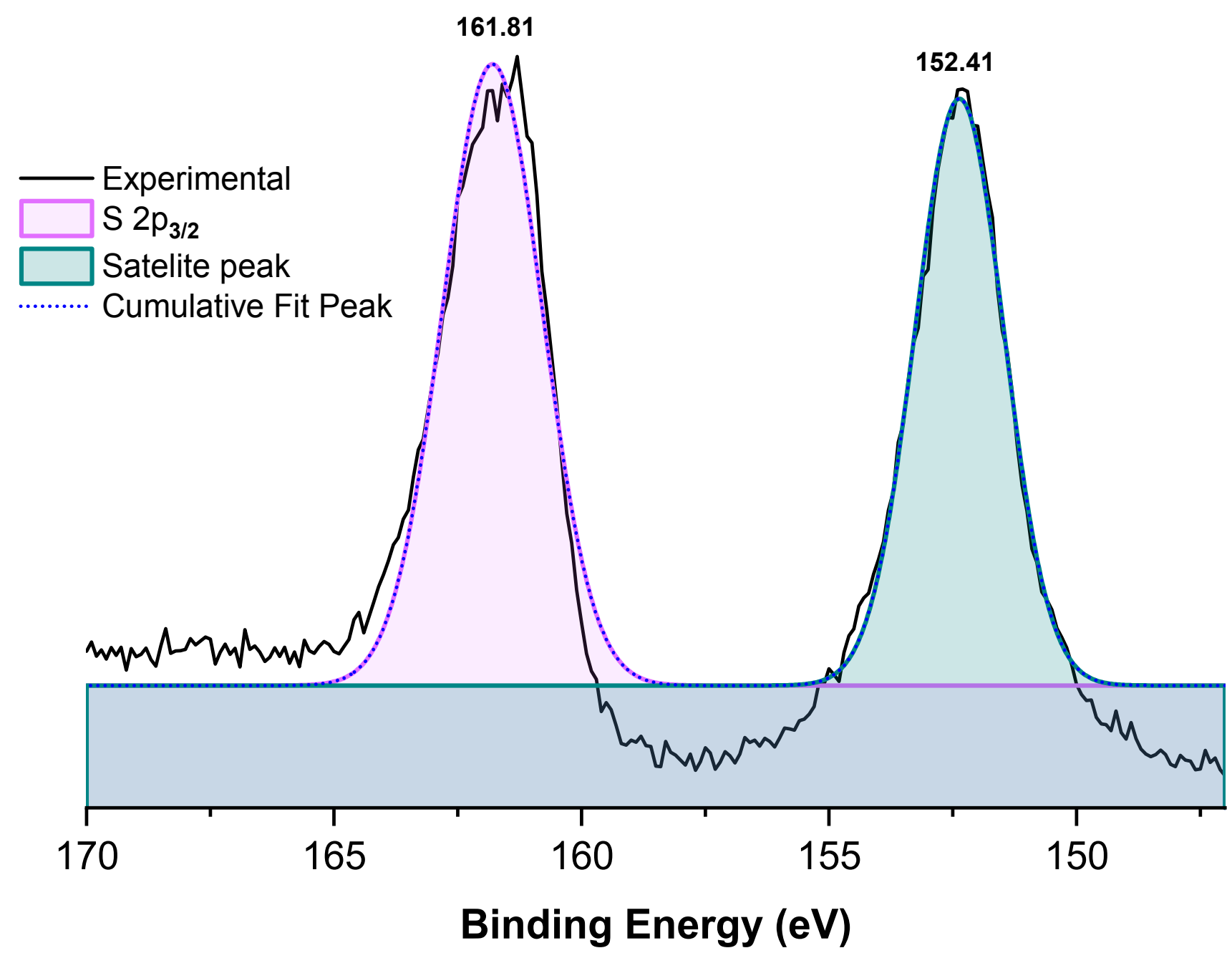
161.81
152.41
Experimental
S 2p3/2
Satelite peak
Cumulative Fit Peak
170
165
160
155
150
Binding Energy (eV)

(d)

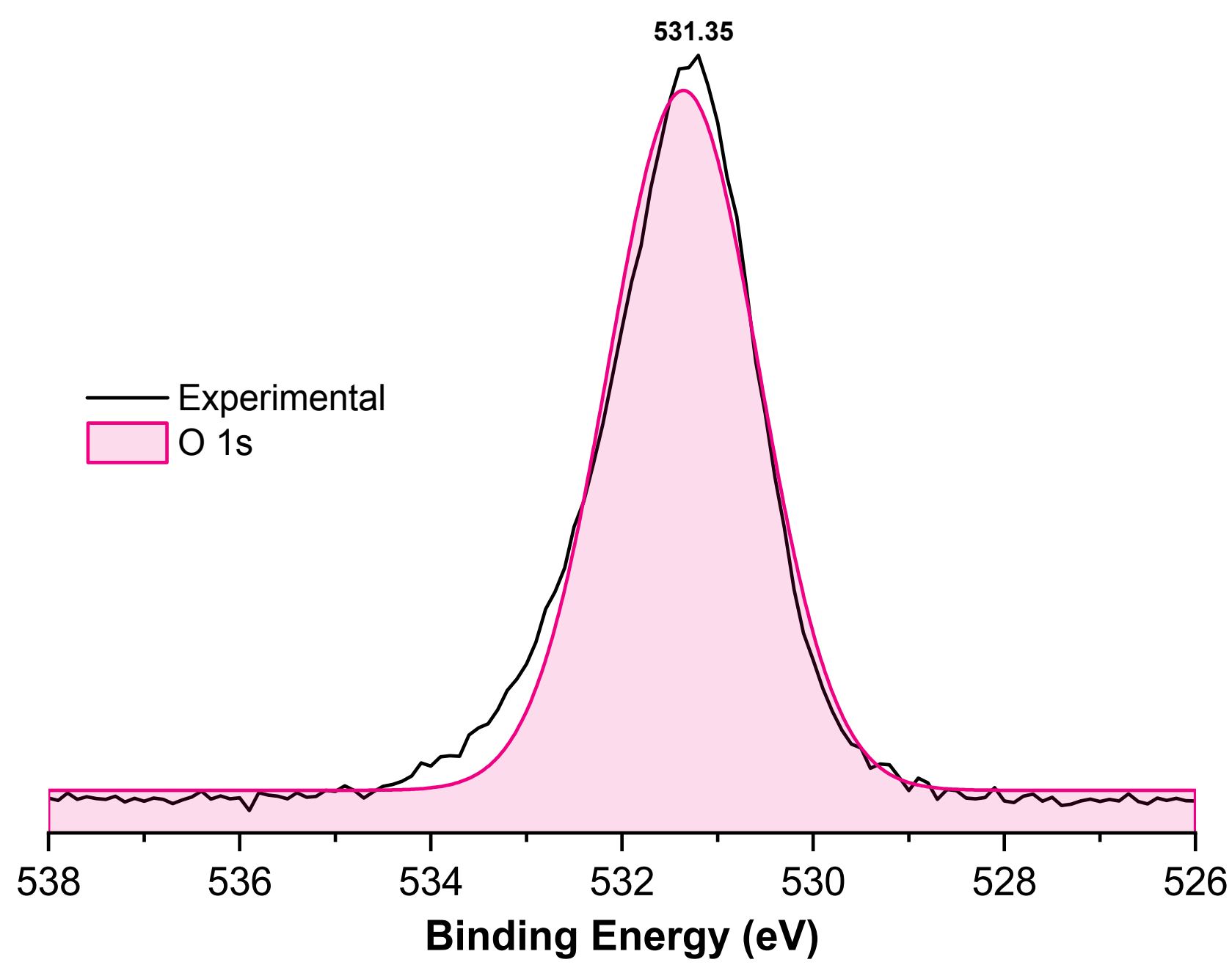
531.35
Experimental
O 1s
538
536
534
532
530
528
526
Binding Energy (eV)

(e)

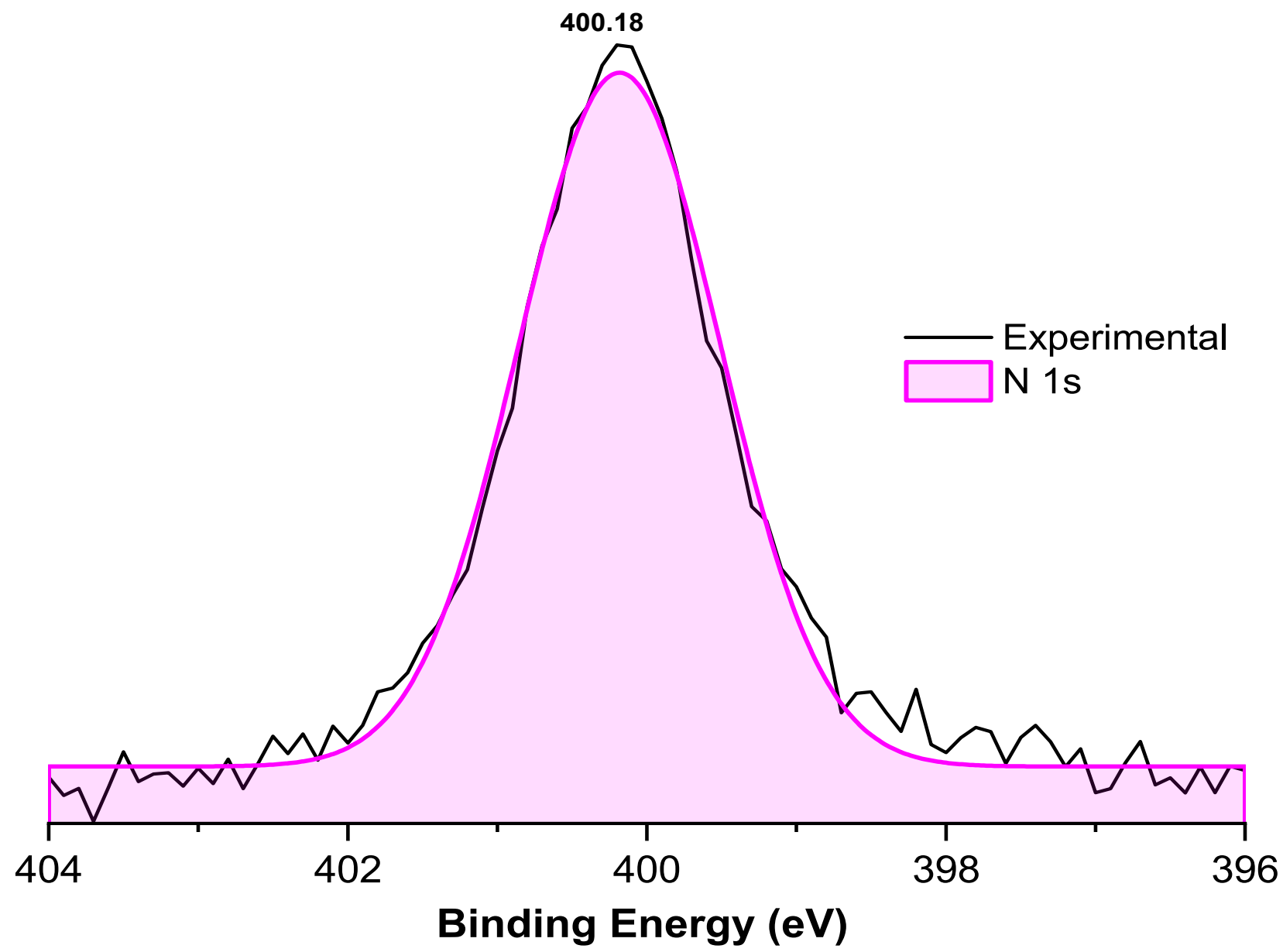

(f)

Figure S8. XPS spectra of (a) Cl 2p, (b) I 3d, (c) C 1s, (d) S 2p, (e) O 1s and (f) N 1s of complex **1**.

Table S5. Assignment of XPS peaks of **1.**

**Elements**	**Peaks (eV)**	**References**
Cl	199.40 ($2p_{1/2}$)<br>197.90 ($2p_{3/2}$)	S14
I	629.37 ($3d_{3/2}$)<br>617.80 ($3d_{5/2}$)	S15
C	284.81 (1s)<br>283.64 (1s)	S16
S	161.81 ($2p_{3/2}$)<br>152.41	S17
O	531.35 (1s)	S18
N	400.18 (1s)	S19

The XPS spectra (Table S5) clearly confirm the presence of key elements and their corresponding chemical states on the sample surface. The Cl 2p peaks observed at 199.40 and 197.90 eV, along with the I 3d peaks at 629.37 and 617.80 eV, exhibit characteristic spin–orbit doublets, indicating the existence of chlorine and iodine in distinct chemical environments. The C 1s signals detected at 284.81 and 283.64 eV suggest the presence of carbon atoms in different bonding environments. In addition, the S $2p_{3/2}$ peak at 161.81 eV is attributed to sulfur species on the surface. The O (531.95 eV) and N (400.18 eV) peaks correspond to oxygen- and nitrogen-containing functional groups, respectively, consistent with commonly observed 1s states. All the peak assignments are consistent with previously reported binding energies, as referenced in Table S5.

10. Powder XRD Analysis:

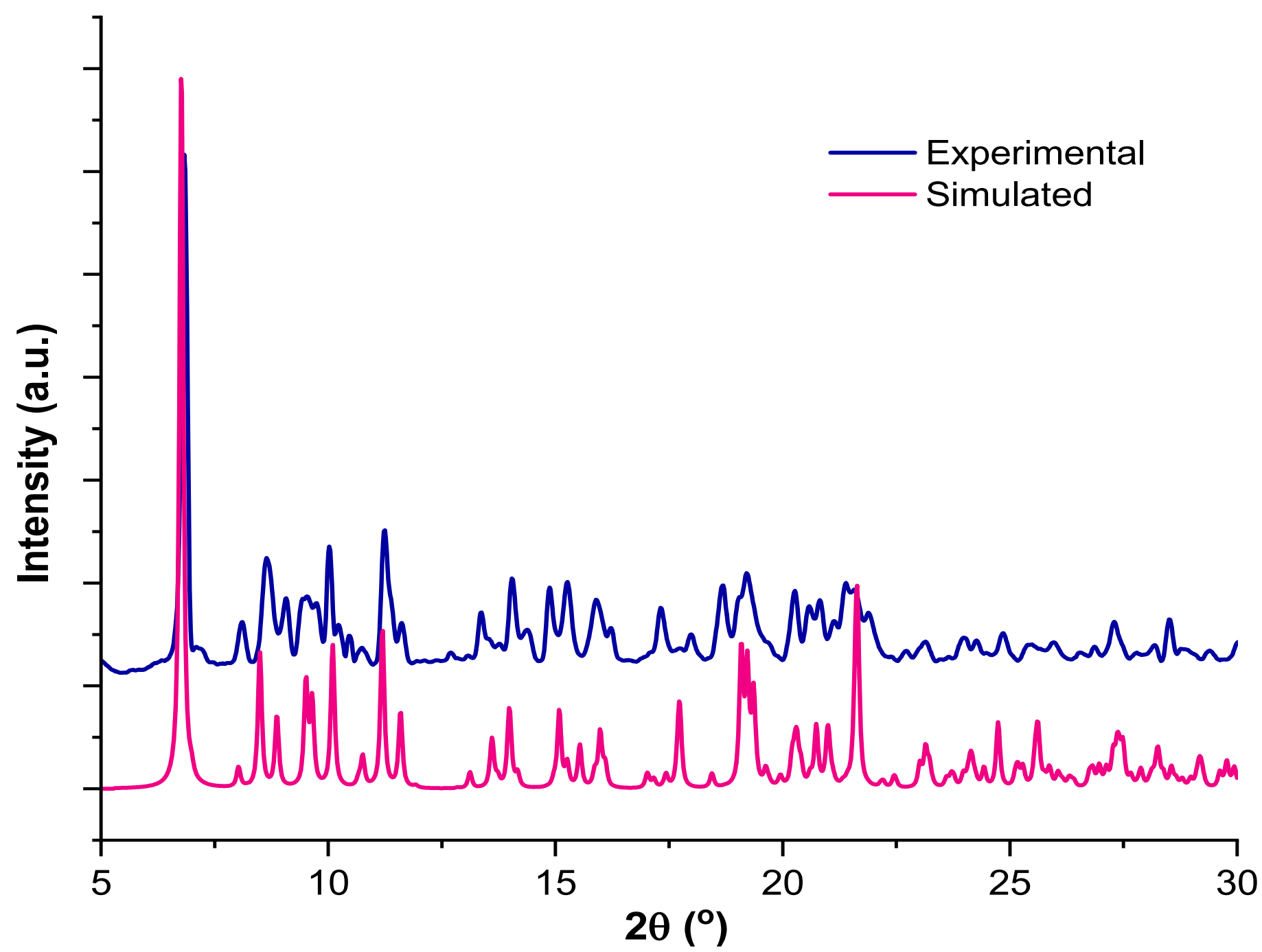

Figure S9. The experimental PXRD pattern (blue) and simulated PXRD pattern from single crystal X-ray diffraction (red) for complex **1.**

11. Thermogravimetric Analysis

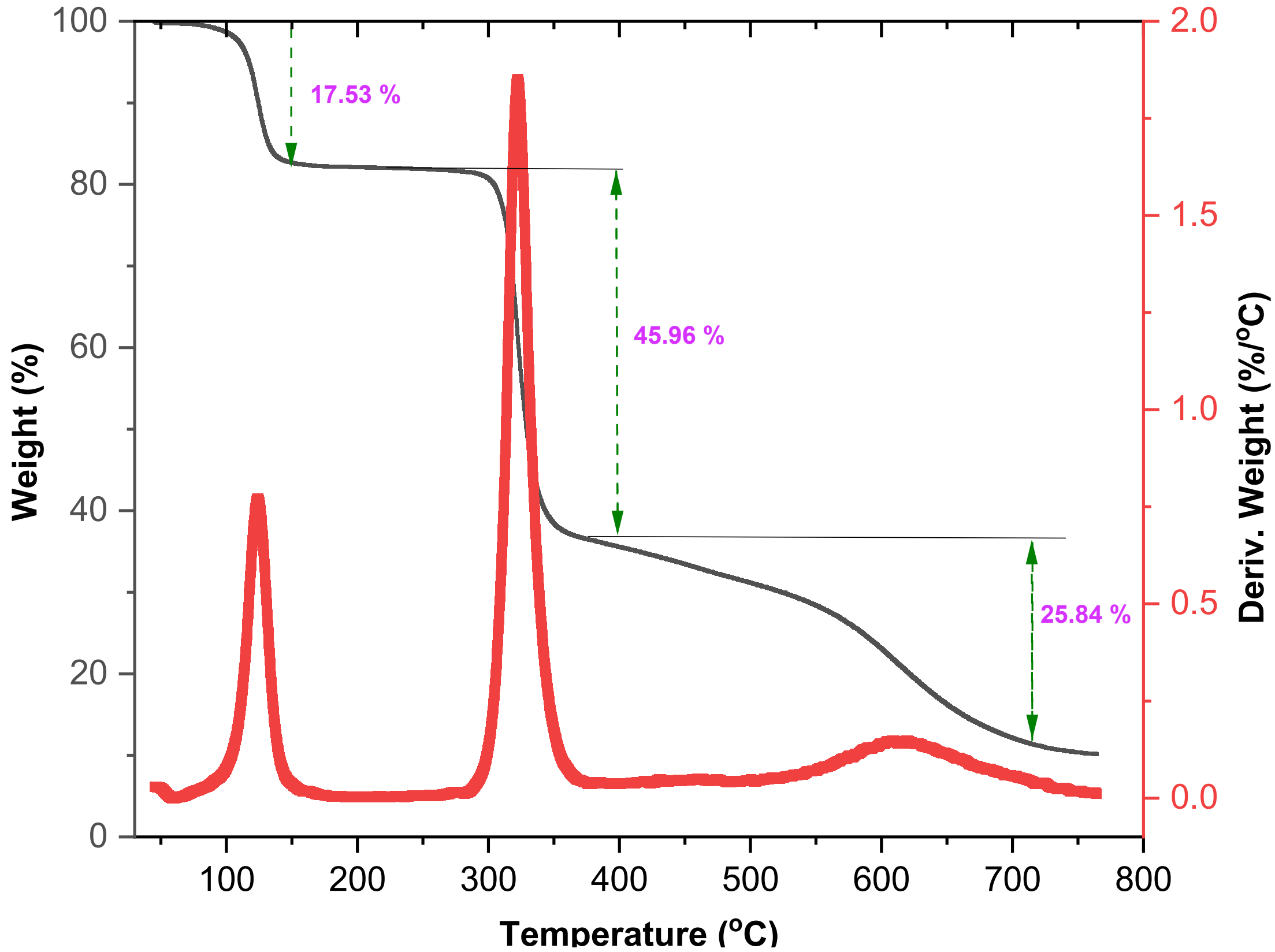

Figure S10. Thermogravimetric analysis plot for complex **1.**

The TGA of complex **1** shows an initial weight loss probable corresponding to the removal of lattice THF molecules at 150 °C, followed by further losses due to the elimination of coordinated THF ligands and subsequent decomposition of the organic framework at 360 °C. The final stable residue can be attributed to cobalt oxide formed at high temperature.

12. Magnetic and structural parameters comparison of the reported Co^{II}_3 complexes showing SMM property.

Table S6. Magnetic and structural parameters comparison of the reported trinuclear Co^{II} complexes showing SMM properties.

No.	Complex (Interaction)	Geometry	J (cm^{-1})	$\chi_m T$ ($cm^3\ mol^{-1}\ K$) (rt)	D (cm^{-1})	U_{eff} (K)	τ_0 (s)	References
1.	$\{[Co_3(pymp)_4(MeOH)_2][BPh_4]_2\}\cdot 2MeOH$<br>$[Co_3(pymp)_4(pya)_2][ClO_4]_2$<br>(FM)	Oh<br>Oh	+2.4<br>+0.65	8.95<br>8.70	-6.5<br>-2.3	18.0(2)<br>7.9(1)	1.9×10^{-8}<br>8.5×10^{-8}	S20
2.	$[(HCDP]_2Co_3(sil)_2Cl_4(THF)_2]\cdot_4THF$<br>(FM)	Oh (central)<br>Td (terminal)	+10.18	7.08	-100<br>-29	65(2)	$4(2) \times 10^{-10}$	S21
3.	$[\{Co(\mu\text{-}N_3O_3L)\}_2Co]$<br>(AFM)	Trigonal Anti-prismatic (central)<br>Trigonal Prismatic (terminal)	-6.38	8.45	-146(2)	43.8(1)	$6.62(2) \times 10^{-8}$	S22
4.	$[(S\text{-}MIC)_2(Co^{II})_3I_2(\mu_2\text{-}Cl)_4(THF)_2]\cdot 4THF$<br>(AFM)	Oh (central)<br>Td (terminal)	-1.105(5)	10.3	-30.8(3)	25(20)	$10^{-8(3)}$	This work

13. Magnetic Measurements

DC/AC Magnetometry

Direct current (DC) magnetization was measured with a Physical Property Measurement System (PPMS) DynaCool from Quantum Design equipped with a vibrating sample magnetometry (VSM) option. 11.9 mg of the powdered complex **1** was pressed into a polypropylene capsule (QDS-4096-388 from Quantum Design) and attached to the brass half- tube sample holder (QDS-4096-391 from Quantum Design). DC magnetization vs. Temperature was measured at a magnetic field of 2500 Oe from 2 K to 50 K in settle mode and from 51 K to 300 K in sweep mode with a 1 K/min heating rate. The temperature step size for signal acquisition was 1 K with a signal averaging time of 10 seconds. Magnetization vs. field was measured at 2, 3, 4, 6, 10, 15, 25, 50, and 300 K up to 7 T with a field step size of 2500 Oe (0.25 T), a signal averaging time of 10 seconds, and a two-fold redundancy per measuring point. At each temperature, the magnetic field was first increased to 7 T and then the field was decreased to zero field again. The superimposed curves for all field scans verified that no reorientation of the sample's powder particles has been induced by the magnetic field. The DC raw data sets have been corrected for diamagnetic contributions

Computational Details: All quantum mechanical calculations were performed using the ORCA 5.0.4 program package. Each Co(II) center was treated independently through a diamagnetic-substitution approach, in which the other two Co(II) ions were replaced by closed-shell Zn(II) ions. State-averaged complete active space self-consistent field (SA-CASSCF) calculations were carried out on the complete X-ray structure of complex **1** using a CAS(7,10) active space for both types of Co(II) sites, i.e. the octahedral Co(2) and the tetrahedral Co(1). This active space distributes seven 3*d* electrons over 10 orbitals (five 3*d* and five 4*d* orbitals, the latter accounting for the double-shell effect). Both quartet (S = 3/2) and doublet (S = 1/2) roots for the d^7 configuration were included: 10 quartet roots (^4F, ^4P) and 40 doublet roots (^2G, ^2P, ^2H, ^2D, ^2D, ^2F), all with equal weights in the state-averaged procedure. To apply dynamic electron correlation and capture the significant covalency and charge-transfer effects arising from the soft sulphur donors at the terminal sites, NEVPT2 calculations with an extended CAS(13,13) active space were performed for the Co(1) ions. This larger active space includes the seven 3*d* electrons together with six electrons from the three sulphur 3*p* orbitals, distributed over 13 orbitals (five 3d, five 4d, and three S 3p orbitals). Because the full X-ray structure makes NEVPT2 calculations with the extended active space computationally prohibitive, a truncated

model of **1** was employed that preserves the structural integrity and the immediate coordination environment of each Co(II) site. All NEVPT2 calculations were performed on this truncated model: with the CAS(13,13) active space for the terminal Co(1) ions and with the CAS(7,10) active space for the central octahedral Co(2) ion. Scalar relativistic effects were included throughout via the second-order Douglas-Kroll-Hess (DKH2) Hamiltonian together with finite-nucleus and picture-change corrections. The DKH-def2-TZVP basis set was used for Co, Zn, and I, and DKH-def2-SVP for all remaining atoms. Auxiliary Coulomb and exchange fitting basis sets were generated automatically via the AutoAux option, and the resolution-of-identity (RI) approximation was applied to accelerate the integral evaluation.

Spin-orbit coupling (SOC) was treated via the quasi-degenerate perturbation theory (QDPT) approach to extract the spin-Hamiltonian parameters (*D*-tensor and *g*-tensor) from the spin-orbit coupled wavefunctions. The SINGLE_ANISO and POLY_ANISO modules implemented in the ORCA package were used to compute the ab initio blocking barrier, Zeeman splitting, magnetic susceptibility at a DC field of 0.25 T, and temperature-dependent magnetization. The intramolecular exchange coupling constants were determined using broken-symmetry DFT (BS-DFT) calculations performed with the B3LYP and *ω*B97X functionals. The def2-TZVP basis set was used for Co, Zn, and I, and def2-SVP for all other atoms; the RIJCOSX approximation with AutoAux auxiliary basis sets was applied throughout.

In the linear Co(1)-Co(2)-Co(1) trimer, adjacent exchange pathways (Co(1)-Co(2)) are described by *J* and the next-nearest-neighbour terminal-terminal pathway (Co(1)...Co(1)) by *J'*. To isolate these interactions via diamagnetic substitution, *J* was obtained from calculations in which one terminal Co(1) was replaced by Zn(II), while *J'* was extracted by replacing the central Co(2). The coupling constants were evaluated using Yamaguchi's spin-projection approach for the exchange Hamiltonian:

$$\hat{H}_{exch} = -J\left(\hat{S}_{Co1}\hat{S}_{Co2} + \hat{S}_{Co2}\hat{S}_{Co1}\right) - J'\hat{S}_{Co1}\hat{S}_{Co3} \quad \text{(eq. S1)}$$

14. Diamagnetic Susceptibility Corrections of Complex 1

Molecular weight: **2026.52** g/mol

Total diamagnetic susceptibility correction (χ_D) = Atomic diamagnetic ($\Sigma_i\chi_{Di}$) + Bond diamagnetic contribution ($\Sigma_i\lambda_i$)

- Atomic diamagnetic contribution to magnetic susceptibility $(\Sigma_i\chi_{Di})_1$

= 26 x $\chi_{C(\text{open})}$ + 50 x $\chi_{C(\text{ring})}$ + 4 x $\chi_{N(\text{ring})}$ + 2 x χ_S + 3 x χ_{Co} + 4 x χ_{Cl} + 2 x χ_I + 2 x χ_O + 100 x χ_H

= -[26 x (6.00) + 50 x (6.24) + 4 x (4.61) + 2 x (15.0) + 3 x (12) + 4 x (20.1) + 2 x (44.6) + 2 x (4.6) + 100 x (2.93)] x (10^{-6} emu. mol^{-1})

= -1024.24 x 10^{-6} emu. mol^{-1}

- For, a total 4THF solvent of crystallization:

$(\Sigma_i\chi_{Di})_2$ = 16 x $\chi_{C(\text{ring})}$ + 4 x χ_O + 32 x χ_H

= -[16 x (6.24) + 4 x (4.6) + 32 x (2.93)] x (10^{-6} emu. mol^{-1})

= -212 x 10^{-6} emu. mol^{-1}

- Bond diamagnetic contribution to magnetic susceptibility ($\Sigma_i\lambda_i$)

= $(\lambda_i)_{6(\text{Benzene})}$ + $(\lambda_i)_{6(\text{Tetrahydrouran})}$ + $(\lambda_i)_{2(\text{Imidazole})}$ + $(\lambda_i)_{4(\text{Ar-Ar})}$

= [6 x (-1.4) + 6 x (0.0) + 2 x (+8.0)+ 4 x (-0.5)] x 10^{-6} emu mol^{-1}

= + 5.6 x 10^{-6} emu. mol^{-1}

Therefore,

Total diamagnetic susceptibility correction (χ_D)

= $\Sigma_i\chi_{Di}$ + $\Sigma_i\lambda_i$

= [-1024.24-212+5.6] x 10^{-6} emu. mol^{-1}

= -1230.64 x 10^{-6} emu. mol^{-1}

15. Broken-Symmetry Evaluation of The Magnetic Exchange Interactions

Table S7. Spin expectation values, deviations, and magnetic exchange coupling (*J*) for various theoretical levels in Co-Co interactions.

Interaction	Theory Level	⟨*S*⟩ (High Spin)	$\langle S^2 \rangle$ (High Spin)	Ideal $\langle S^2 \rangle$ (*S*=3)	Deviation (High Spin)	$\langle S^2 \rangle$ (BrokenSym)	Deviation (Broken Symmetry)	*J* (cm^{-1})
J	B3LYP	3	12.013439	12.000000	0.013439	3.0075	3.0075	−3.48
J'	B3LYP	3	12.016421	12.000000	0.016421	3.0159	3.0159	−1.15
J	wB97	3	12.013515	12.000000	0.013515	3.0098	3.0098	−1.68
J'	wB97	3	12.015286	12.000000	0.015286	3.0151	3.0151	−0.77

16. a. Ab-initio Calculations for Both Type of Co sites in Complex 1

We used ORCA 5.0.4 for SA-CASSCF(7,10) on full X-ray structure of complex **1** to compute low-lying energy spectrum of both types of Co sites (middle and terminal sites).

Table S8. Main values of the g tensor for the ground Kramers doublets within the formalism of pseudospin *S* = 1/2 for the Co(1) and Co(2) site with CAS(7,10). Zero-field splitting parameters are also listed (for full X-ray structure)

Co-site	Method	*D* (cm^{-1})	*E/D*	g_x^{KD1} g_x^{KD2}	g_y^{KD1} g_y^{KD2}	g_z^{KD1} g_z^{KD2}
Co (2)	2nd order SOC	-103.537	0.145			
	Effective Hamiltonian	-76.297	0.267	1.40	1.89	8.34

				2.54	2.93	5.21
Co (1)	2nd order SOC	-22.371	0.328			
	Effective Hamiltonian	-17.970	0.299	1.55<br>2.02	2.18<br>2.61	7.1<br>6.11

Table S9. Kramers doublet (KD) energies obtained from the spin-orbit coupled states of the Co(1) and Co(2) sites. Energies are reported relative to the ground doublet (KD1 = 0 cm^{-1}) (for full X-ray structure).

Kramers Doublet (Co(2))	Energy (cm^{-1})
KD1	0.00
KD2	168.13
KD3	987.00
KD4	1273.68
KD5	1410.78
KD6	1522.66

Kramers Doublet (Co(1))	Energy (cm^{-1})
KD1	0.00
KD2	40.47
KD3	2006.56
KD4	2167.87
KD5	2856.68
KD6	2984.45

Table S10. Multi-configurational wavefunction description and their contribution towards D and E/D parameters based on CAS(7,5) SA-CASSCF calculation for the octahedral middle Co(2) site in complex **1**.

Energy (cm^{-1})	d_{z^2}	d_{xz}	d_{yz}	$d_{x^2-y^2}$	d_{xy}	Weight (%)	Contribution towards D (cm^{-1})	Contribution towards E (cm^{-1})

0.0	1	2	1	2	1	76.23	0.000	0.000
	1	2	1	1	2	7.36		
783.6	1	1	2	2	1	70.05	-140.663	-0.441
	1	1	2	1	2	8.76		
968.0	1	2	2	1	1	78.94	26.040	-27.082
	1	1	1	2	2	15.58		

Table S11. Multi-configurational wavefunction description and their contribution towards *D* and *E/D* parameters based on CAS(7,5) SA-CASSCF calculation for the tetrahedral terminal Co(1) sites in complex **1**.

Energy (cm^{-1})	d_{z^2}	d_{xz}	d_{yz}	$d_{x^2-y^2}$	d_{xy}	Weight (%)	Contribution towards *D* (cm^{-1})	Contribution towards *E* (cm^{-1})
0.0	2	1	1	1	2	38.48	0.000	0.000
	1	1	2	1	2	27.96		
1865.0	2	1	1	2	1	39.26	-56.704	-0.267
	1	1	2	2	1	28.10		
2661.1	1	2	1	1	2	28.48	19.482	4.367
	2	1	2	1	1	19.89		

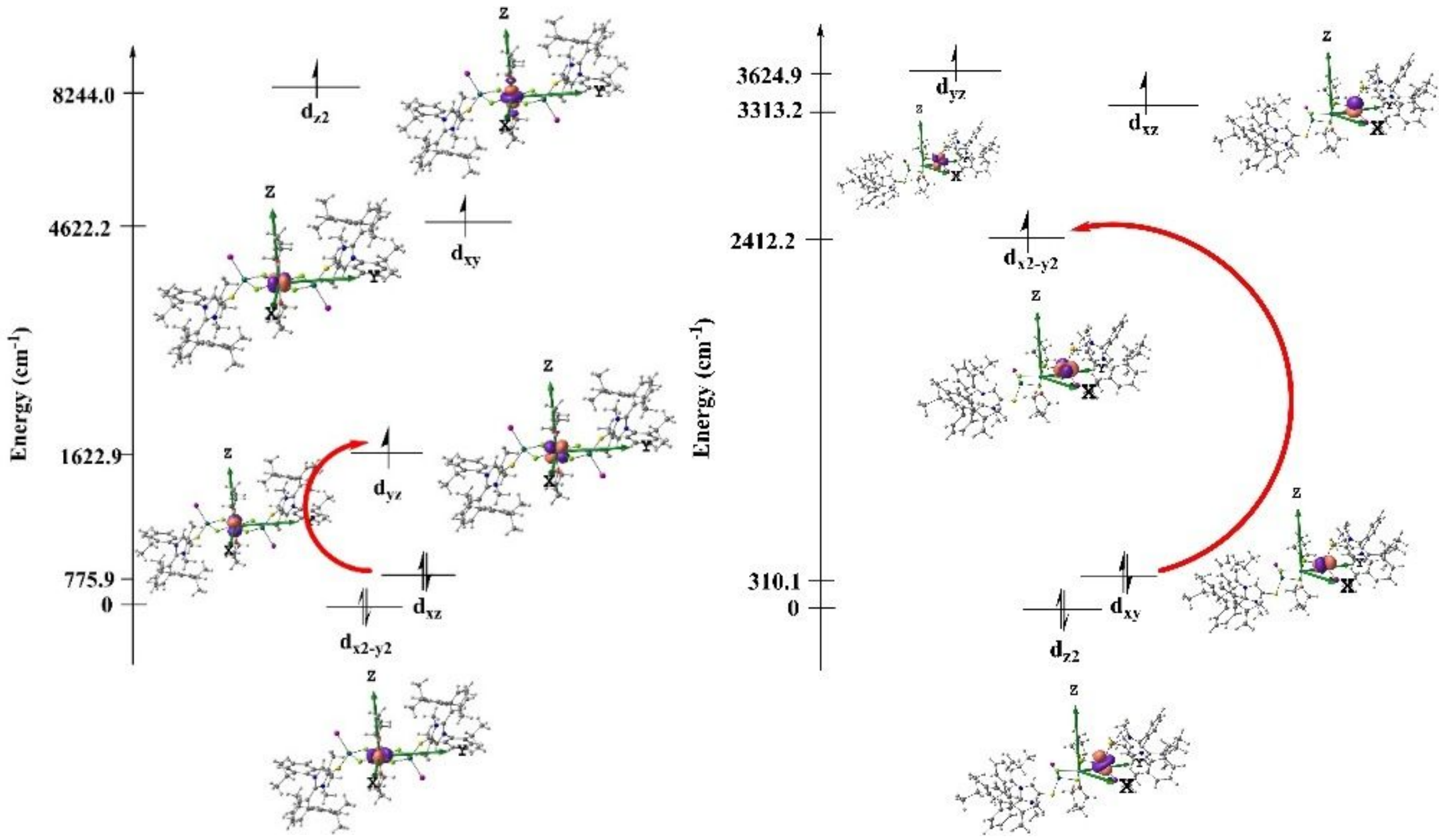

Figure S11. Main electronic configuration of the ground state and AILFT computed d-orbital energy diagram based on CAS(7,5) SA-CASSCF: A for Co(2) and B for Co(1) in complex **1**.

Then we used truncated version of complex **1** for mentioned reasons in main text to perform NEVPT2 and extend the active space for terminal Co ions. The low-lying energy spectrum of this version does not change significantly at SA-CASSCF(7,10) level from full X-ray structure computational calculations, validating the truncation of complex **1** and utilizing it for extending active space and dynamical correlations.

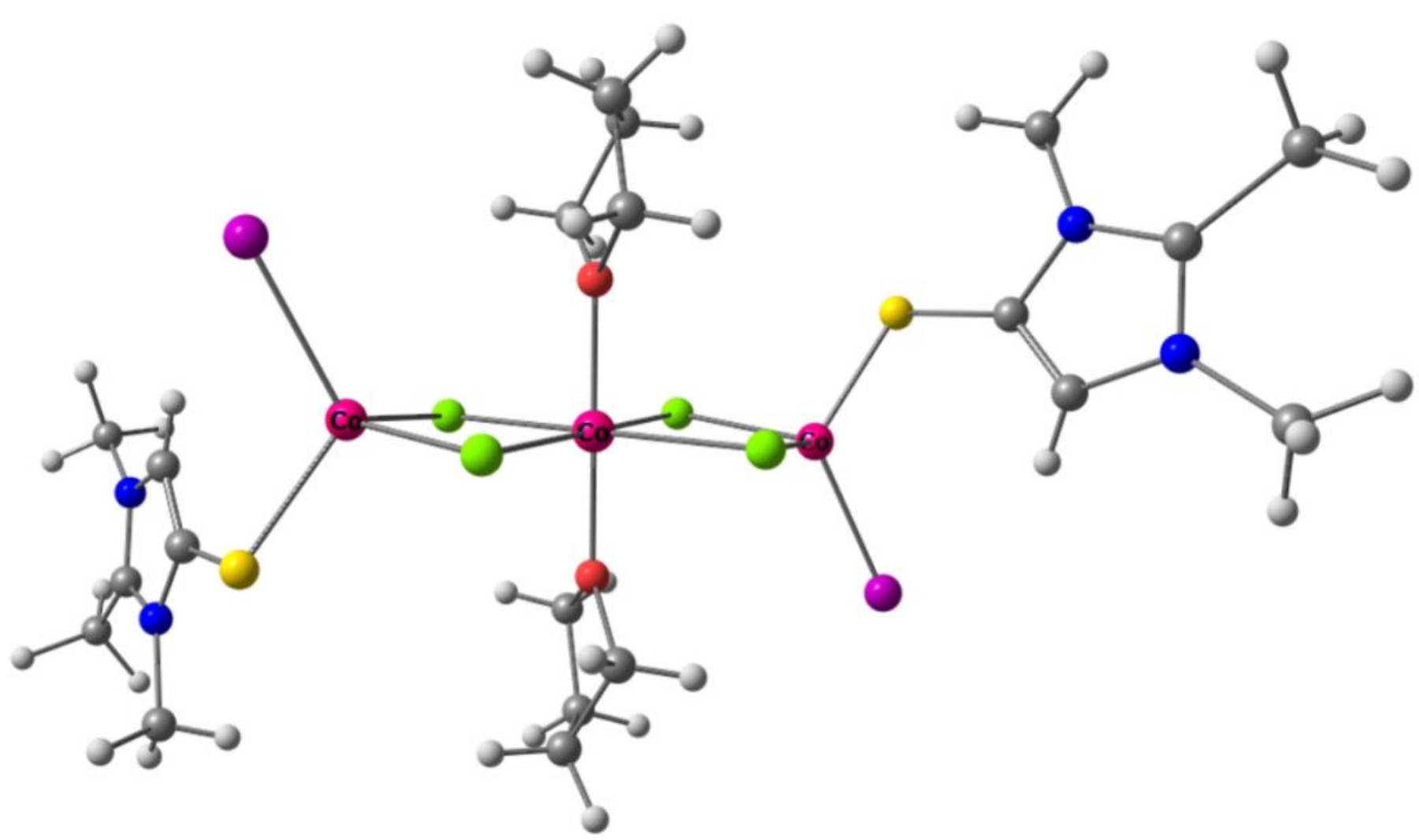

Figure S12. Truncated version of full X-ray structure of complex **1**.

Spin-Free State Energies for truncated version of complex **1**.

Table S12: NEVPT2 spin-free quartet state energies (cm^{-1})

Root	Co(2)	Co(1)
1	0	0
2	1091	2425
3	1255	3654
4	5640	4165
5	6303	5336
6	7640	6080
7	13302	7021
8	18523	17237
9	21867	17522
10	22699	19365

The octahedral Co(2) shows closely spaced low-lying quartets (0, 1091, 1255 cm^{-1}), arising from the $^4T_1$g ground term. This quasi-degeneracy drives the exceptionally large single-ion anisotropy. The tetrahedral Co(1) have a larger first gap (2425 cm^{-1}), consistent with a $^4A_2$ ground state.

Table S13: NEVPT2+SOC Kramers doublet energies (cm^{-1}).

KD	Co(2)	Co(1)
1	0.0	0.0
2	145.8	44.9
3	1141.0	2464.4
4	1409.3	2596.7
5	1544.9	3683.3
6	1666.3	3775.8

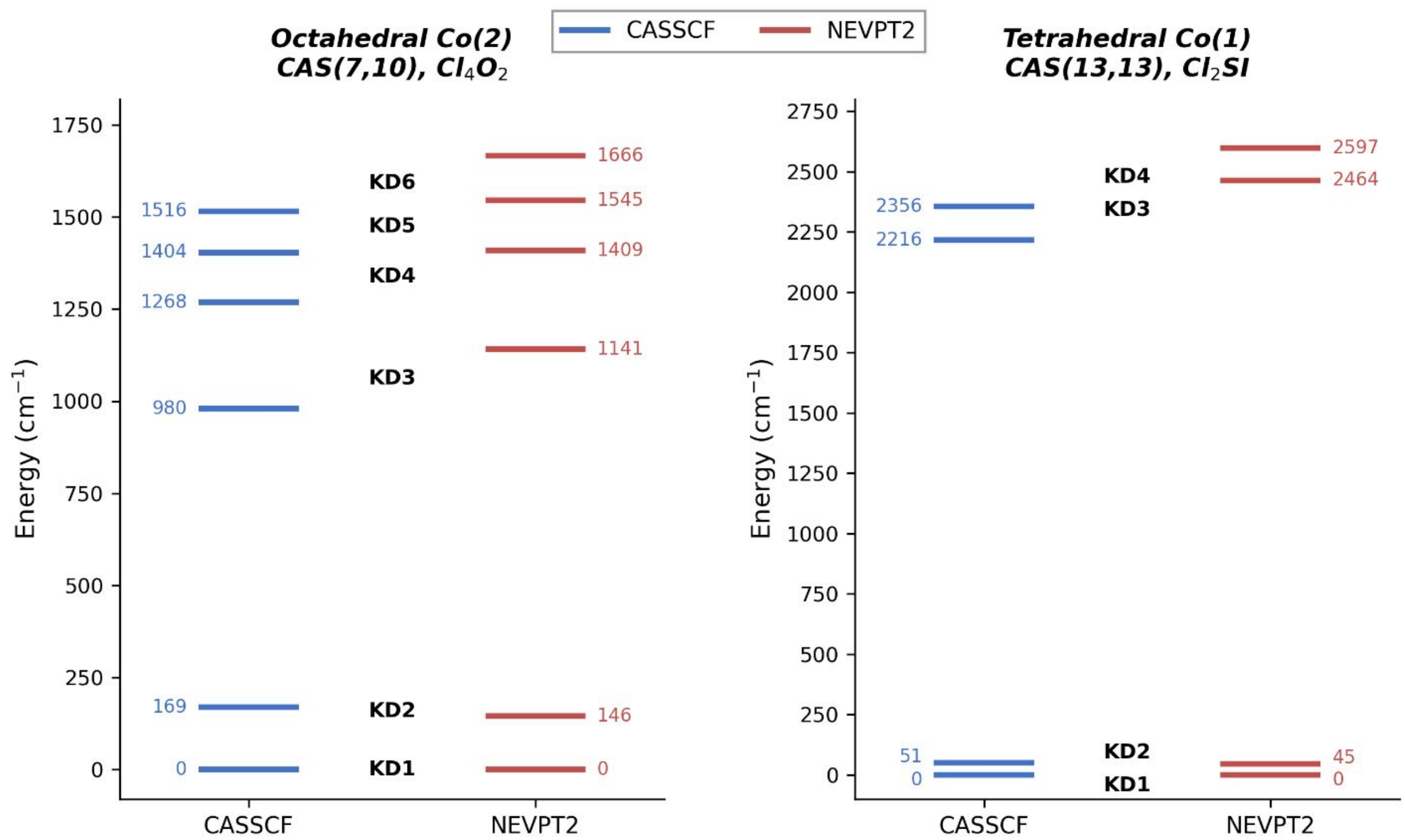

Figure S13: SOC Energy level diagram for central and terminal Co sites with their immediate coordination core and effect of NEVPT2 dynamical correlation correction on CASSCF energy levels.

16. b. Prediction of QTM Rate and Effective Barrier from Ground KD g-factors

The QTM rate and effective energy barrier of the ground exchange-coupled Kramers doublet were estimated following the approach described by Yin and Li.[S23]

Constants: $b = 9.2741 \times 10^{-24}$ J T^{-1}, $h = 6.6261 \times 10^{-34}$ J s, $B_{ave} = 20$ mT, $k_B = 0.69504$ cm^{-1} K^{-1}.

Table S14. Exchange-coupled Kramers doublets (KDs) derived from POLY_ANISO analysis.

KD_i	E_i (cm^{-1})	g_x	g_y	g_z
1	0.000	0.219	0.289	7.101
2	7.262	0.160	0.509	7.124
3	7.907	0.290	0.454	7.243

4	23.377	0.129	0.287	20.201

1. QTM Rate of the Ground KD

Working equation:

$$\tau_{\mathrm{QTM}}^{-1} = \frac{\beta B_{\mathrm{ave}}}{h} \times \frac{g_{\mathrm{XY}}{}^2}{2\left(g_{\mathrm{XY}}{}^2 + g_{\mathrm{Z}}{}^2\right)^{1/2}}$$

Step 1. Transversal *g*-component

$$g_{\mathrm{XY}} = \sqrt{(0.219)^2 + (0.289)^2} = \sqrt{0.04796 + 0.08352} = \sqrt{0.13148} = 0.3626$$

Step 2. Effective rate

$$\tau_{\mathrm{QTM}}^{-1,eff} = \frac{0.1315}{2 \times \sqrt{0.1315 + 50.424}} = \frac{0.1315}{2 \times 7.110} = \frac{0.1315}{14.220} = 9.246 \times 10^{-3}$$

Step 3. Prefactor

$$\frac{\beta B_{\mathrm{ave}}}{h} = \frac{9.2741 \times 10^{-24}\ \mathrm{J\,T^{-1}} \times 0.020\ \mathrm{T}}{6.6261 \times 10^{-34}\ \mathrm{J\,s}} = 2.799 \times 10^{8}\ \mathrm{s^{-1}}$$

Step 4. QTM rate and time

$$\tau_{\mathrm{QTM}}^{-1} = 2.799 \times 10^{8} \times 9.246 \times 10^{-3} = 2.589 \times 10^{6}\ \mathrm{s^{-1}}$$

$$\tau_{\mathrm{QTM}} = 3.86 \times 10^{-7}\ \mathrm{s} \approx 386\ \mathrm{ns}$$

2. Effective Barrier via TA-QTM (KDs 1-4)

Working equations:

$$\tau_{\mathrm{QTM,i}}^{-1,eff} = \frac{g_{\mathrm{XY,i}}{}^2}{2\left(g_{\mathrm{XY,i}}{}^2 + g_{\mathrm{Z,i}}{}^2\right)^{1/2}}$$

$$U_{\mathrm{eff}}(T) = \sum \frac{w_i}{N} \cdot E_i$$

$$w_i = \frac{\exp\left(-E_i/k_{\mathrm{B}}T\right)}{Z} \cdot \tau_{\mathrm{QTM,i}}^{-1,eff}, \quad N = \sum w_i$$

Step 1. $\tau_{\mathrm{QTM,i}}^{-1,eff}$ for each KD

KD 1:

$$g_{\mathrm{XY,1}} = \sqrt{(0.219)^2 + (0.289)^2} = \sqrt{0.13148} = 0.3626$$

$$\tau_{\mathrm{QTM,1}}^{-1,eff} = \frac{(0.3626)^2}{2\sqrt{(0.3626)^2 + (7.101)^2}} = \frac{0.1315}{14.220} = 9.246 \times 10^{-3}$$

KD 2:

$$g_{\mathrm{XY,2}} = \sqrt{(0.160)^2 + (0.509)^2} = \sqrt{0.28468} = 0.5336$$

$$\tau_{\mathrm{QTM,2}}^{-1,eff} = \frac{(0.5336)^2}{2\sqrt{(0.5336)^2 + (7.124)^2}} = \frac{0.2847}{14.288} = 1.993 \times 10^{-2}$$

KD 3:

$$g_{\mathrm{XY,3}} = \sqrt{(0.290)^2 + (0.454)^2} = \sqrt{0.29022} = 0.5387$$

$$\tau_{\mathrm{QTM,3}}^{-1,eff} = \frac{(0.5387)^2}{2\sqrt{(0.5387)^2 + (7.243)^2}} = \frac{0.2902}{14.526} = 1.998 \times 10^{-2}$$

KD 4:

$$g_{\mathrm{XY,4}} = \sqrt{(0.129)^2 + (0.287)^2} = \sqrt{0.09901} = 0.3147$$

$$\tau_{\mathrm{QTM,4}}^{-1,eff} = \frac{(0.3147)^2}{2\sqrt{(0.3147)^2 + (20.201)^2}} = \frac{0.09901}{40.407} = 2.450 \times 10^{-3}$$

Table S15. Exchange-coupled Kramers doublets (KDs) derived from POLY_ANISO analysis together with the corresponding effective QTM rates.

**KD$_i$**	E_i **(cm^{-1})**	$g_{xy,i}$	$g_{Z,i}$	$\tau_{QTM,i}^{-1,eff}$

1	0.000	0.363	7.101	9.246×10^{-3}
2	7.262	0.534	7.124	1.993×10^{-2}
3	7.907	0.539	7.243	1.998×10^{-2}
4	23.377	0.315	20.201	2.450×10^{-3}

Step 2. Boltzmann factors at T = 300 K

$$k_B T = 0.69504 \times 300 = 208.51 \text{ cm}^{-1}$$

$$\exp(-E_1/k_B T) = 1.0000$$

$$\exp(-E_2/k_B T) = 0.9658$$

$$\exp(-E_3/k_B T) = 0.9628$$

$$\exp(-E_4/k_B T) = 0.8939$$

$$Z = 1.0000 + 0.9658 + 0.9628 + 0.8939 = 3.8225$$

Step 3. Weighted rates

$$w_1 = \frac{1.0000}{3.8225} \times 9.246 \times 10^{-3} = 2.419 \times 10^{-3}$$

$$w_2 = \frac{0.9658}{3.8225} \times 1.993 \times 10^{-2} = 5.034 \times 10^{-3}$$

$$w_3 = \frac{0.9628}{3.8225} \times 1.998 \times 10^{-2} = 5.032 \times 10^{-3}$$

$$w_4 = \frac{0.8939}{3.8225} \times 2.450 \times 10^{-3} = 5.730 \times 10^{-4}$$

Step 4. Normalization

$$N = \sum w_i = 1.306 \times 10^{-2}$$

Step 5. Fractional weights and U_{eff}

$$\frac{w_1}{N} = 0.185, \frac{w_2}{N} = 0.386, \frac{w_3}{N} = 0.385, \frac{w_4}{N} = 0.044$$

$$U_{\text{eff}}(300\,K) = 0.185 \times 0.000 + 0.386 \times 7.262 + 0.385 \times 7.907 + 0.044 \times 23.377$$

$$= 0.000 + 2.800 + 3.047 + 1.026 = 6.9 \text{ cm}^{-1} = 10 \text{ K}$$

KDs 2 and 3 together account for 77.1% of the total weight (38.6% + 38.5%), consistent with the TM-based barrier analysis, which identified D2/D3 cross-relaxation as the dominant pathway. KD 4 contributes only 4.4% because its excellent axiality (g_z = 20.2) strongly suppresses tunnelling.

17. AC Magnetic Susceptibility and Relaxation Dynamics Under Variable Field and Temperature

In the low-frequency limit where $\omega \ll \tau^{-1}$, the sample can maintain equilibrium in the AC field and χ_{T} (isothermal susceptibility) is measured, but in the high-frequency limit $\omega \gg \tau^{-1}$ then the magnetisation of the sample is fixed on the time-scale of ω and χ_{S} (adiabatic susceptibility) is measured. A distribution of relaxation times can be emulated by using the generalized Debye model where the parameter α empirically models the distribution. When α=0 there is no distribution of relaxation times (i.e. the distribution in τ is a delta function and the original Debye model is recovered) and when α=1 there is an infinitely flat distribution of relaxation times and $\chi''=0$. Thus, there is a monotonous but non-linear decrease in the peak value of χ'' as a function of α.

$$\chi_{\mathrm{AC}}(\omega) = \chi_{\mathrm{S}} + \frac{\chi_{\mathrm{T}} - \chi_{\mathrm{S}}}{1 + (i\omega\tau)^{1-\alpha}} \quad \text{(S2)}$$

$$\chi''_{\mathrm{Peak}}(\alpha) = (\chi_{\mathrm{T}} - \chi_{\mathrm{S}}) \frac{\cos\left[\frac{\pi\alpha}{2}\right]}{2\left(1 + \sin\left[\frac{\pi\alpha}{2}\right]\right)} \quad \text{(S2b)}$$

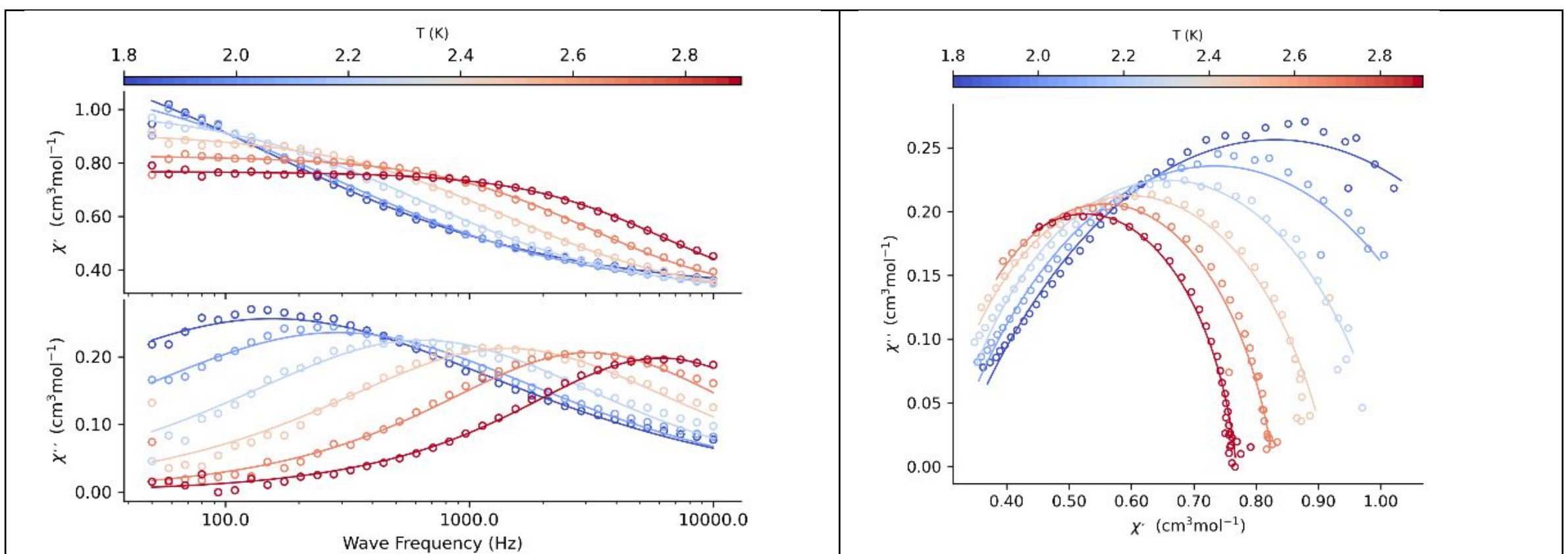

H_{DC} = 0 Oe

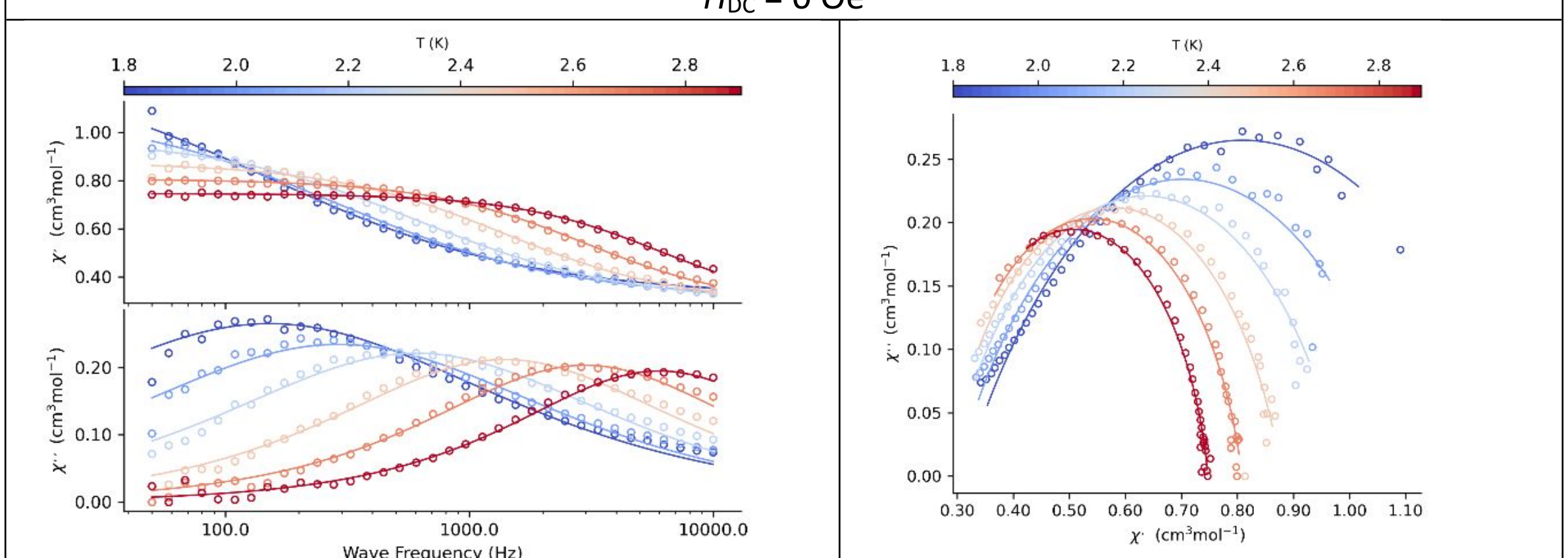

H_{DC} = 2.6 Oe

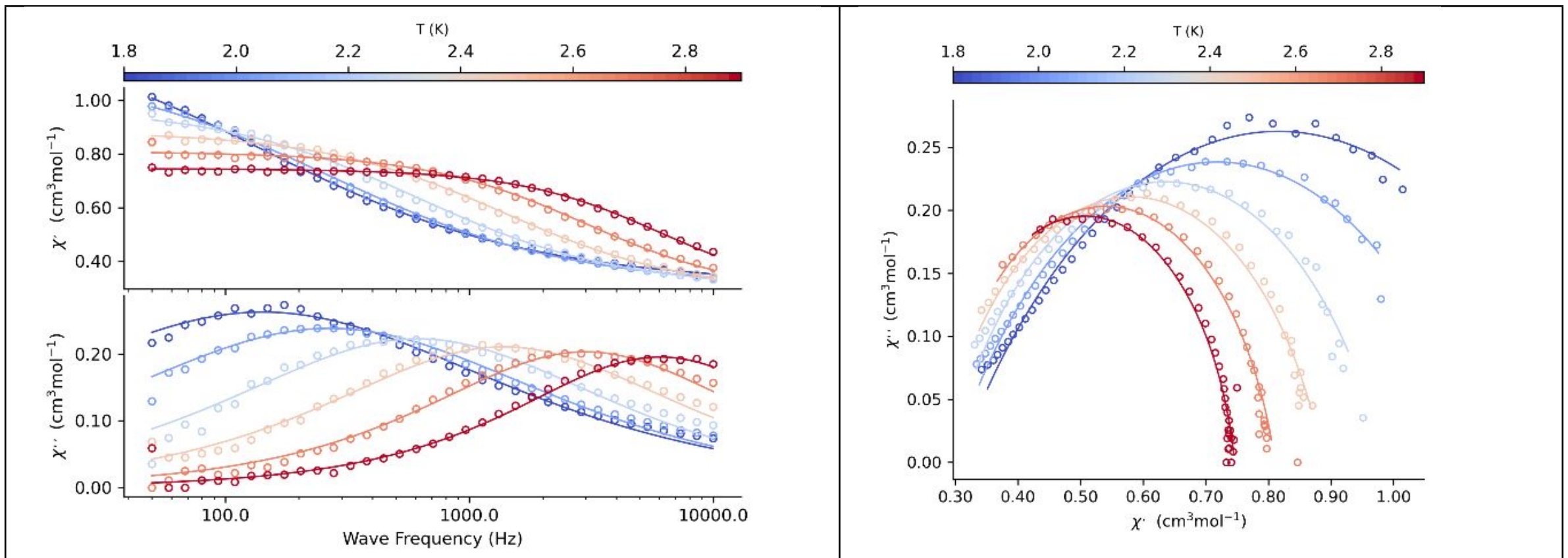

H_{DC} = 4.1 Oe

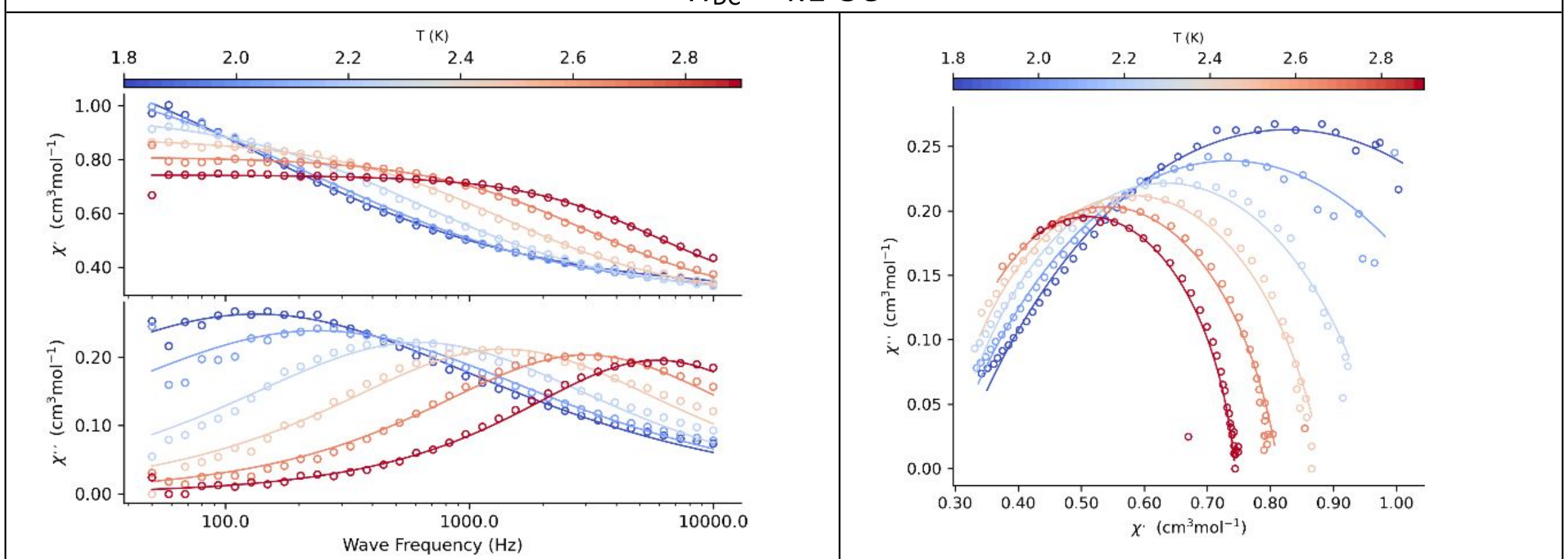

H_{DC} = 6.4 Oe

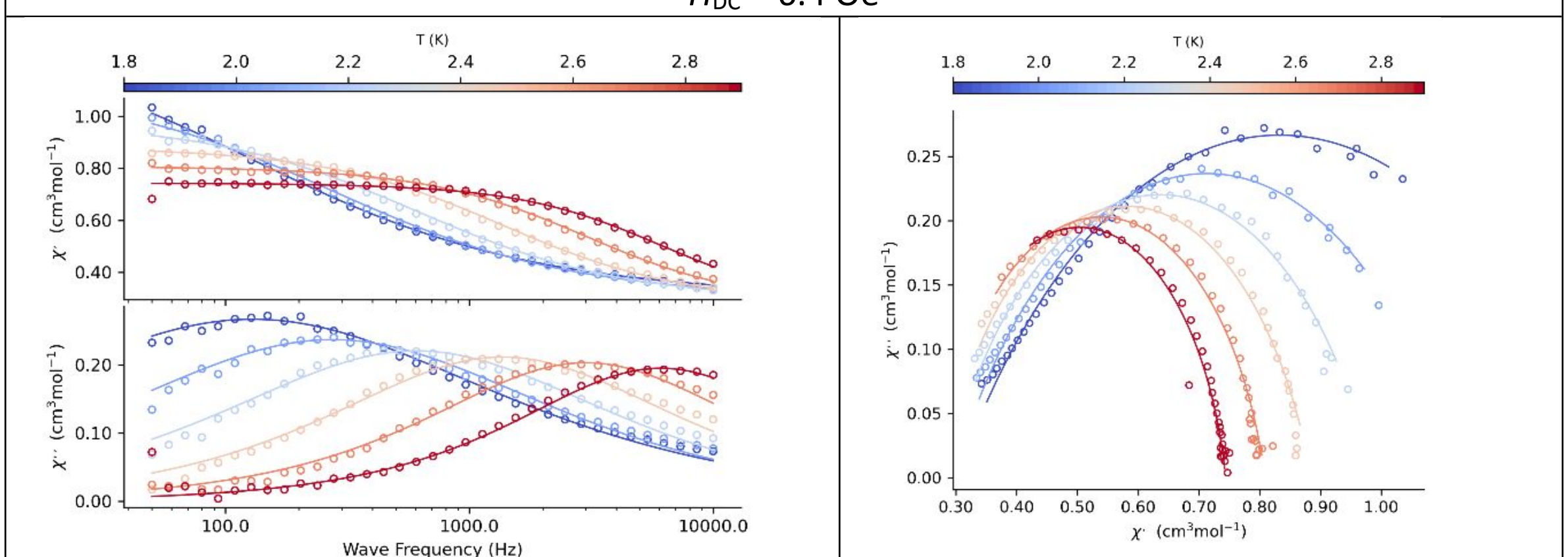

H_{DC} = 10 Oe

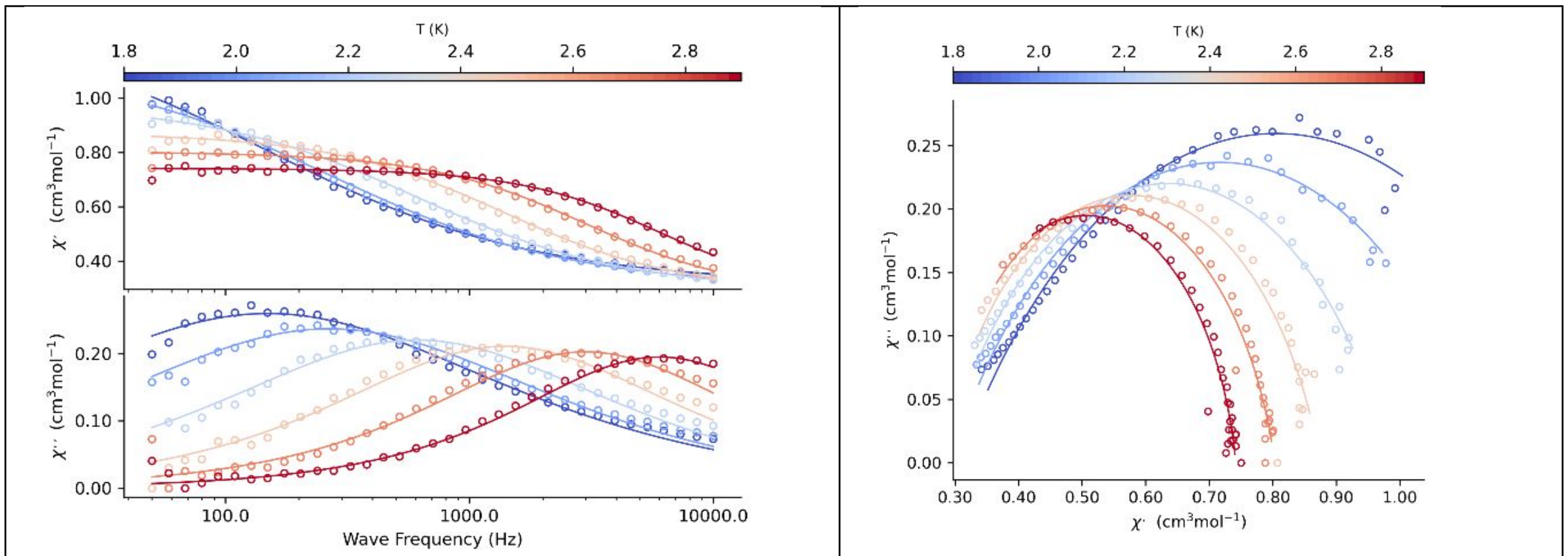

H_{DC} = 15 Oe

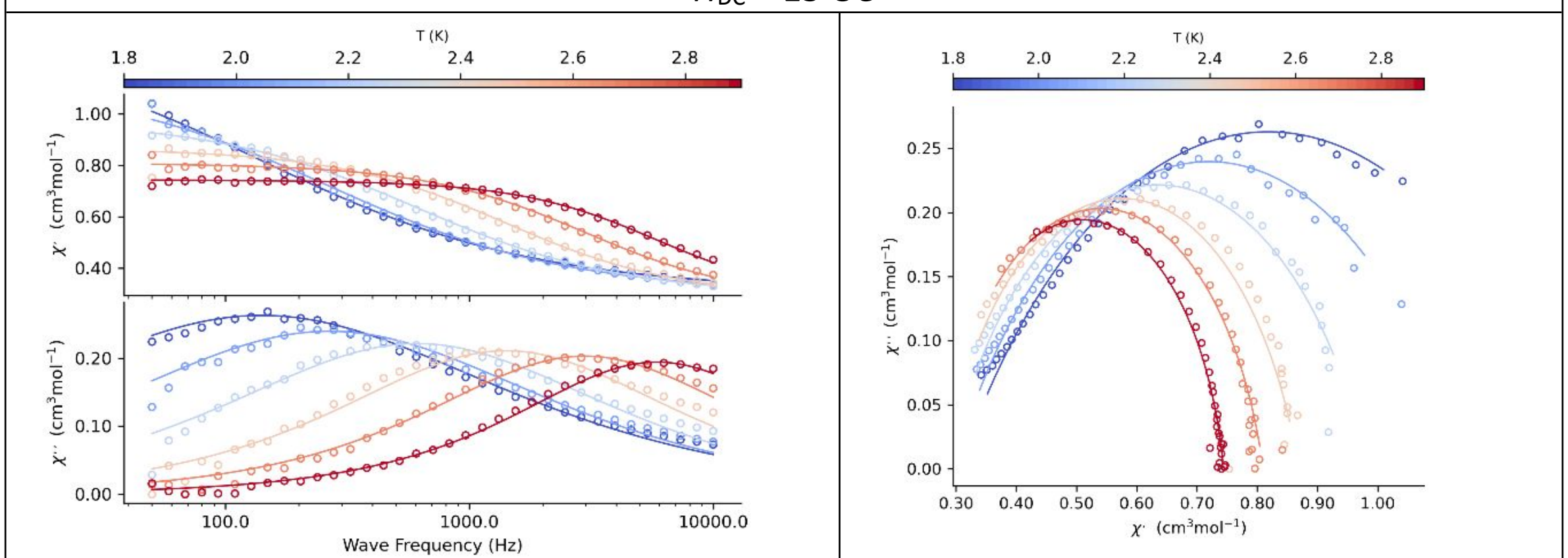

H_{DC} = 24 Oe

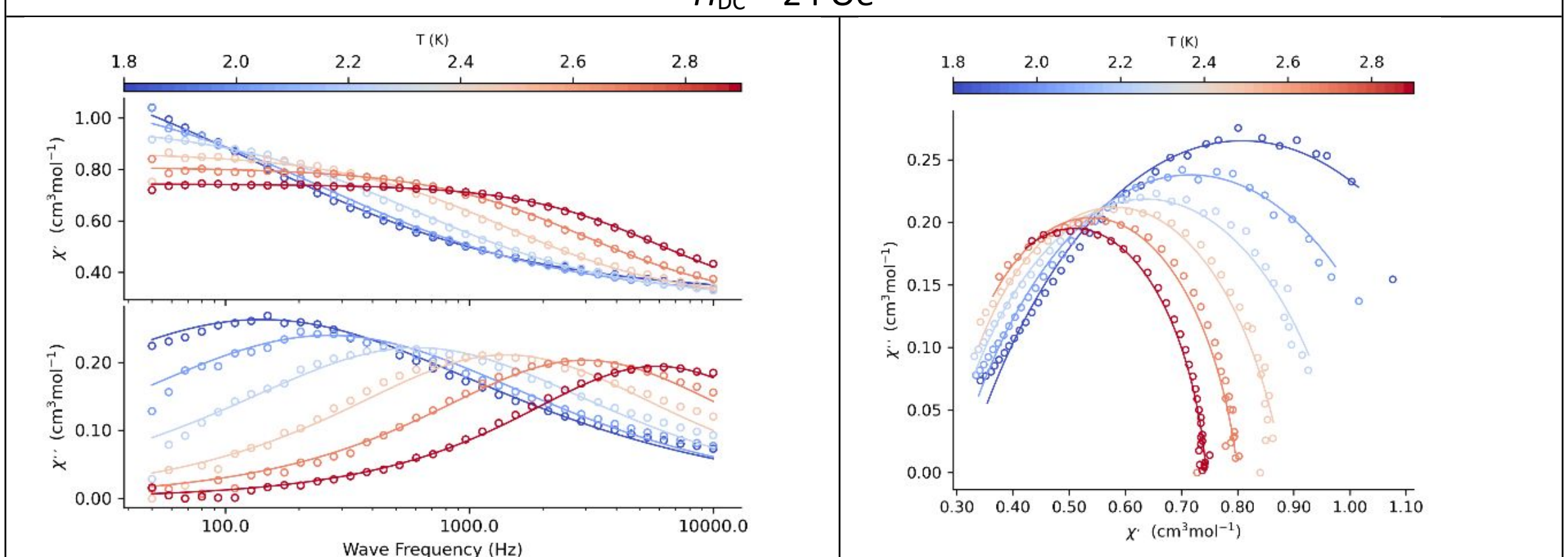

H_{DC} = 37 Oe

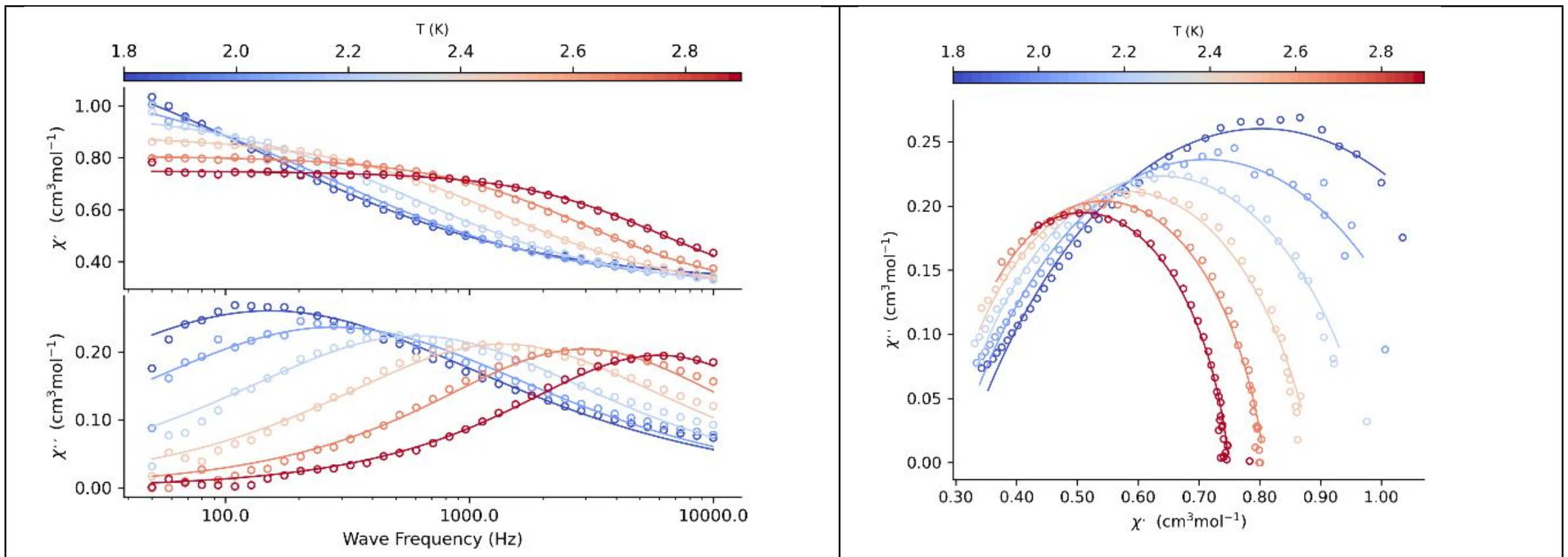

H_{DC} = 59 Oe

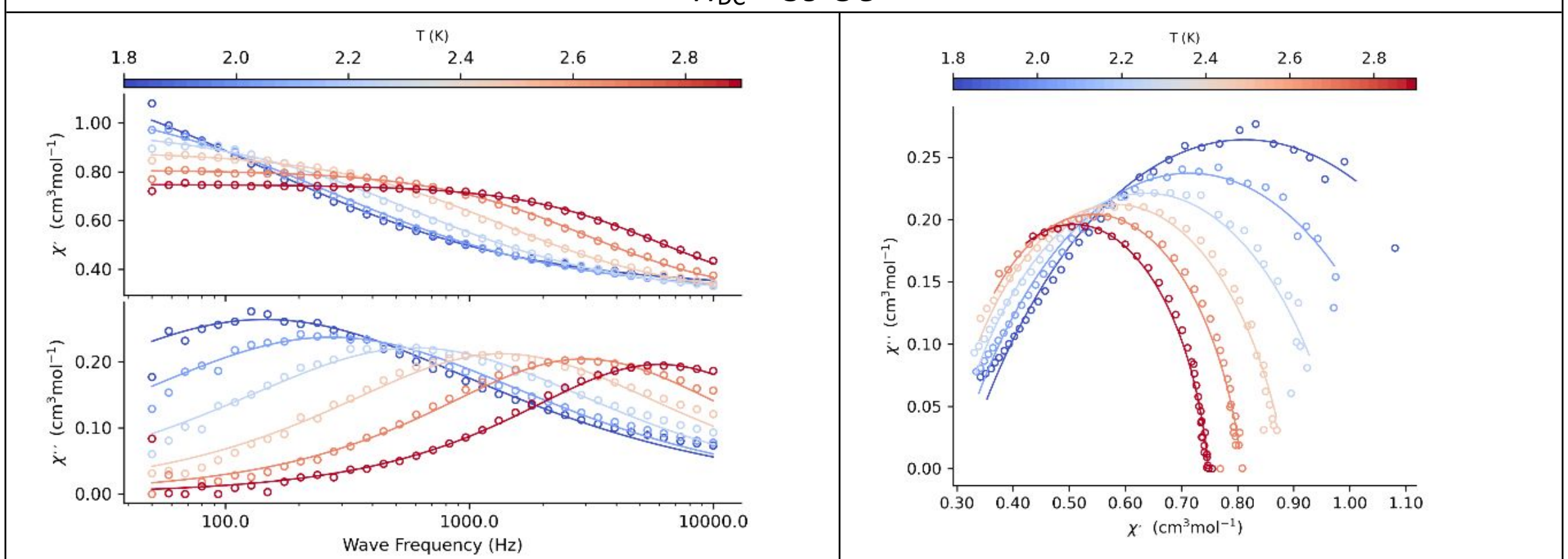

H_{DC} = 92 Oe

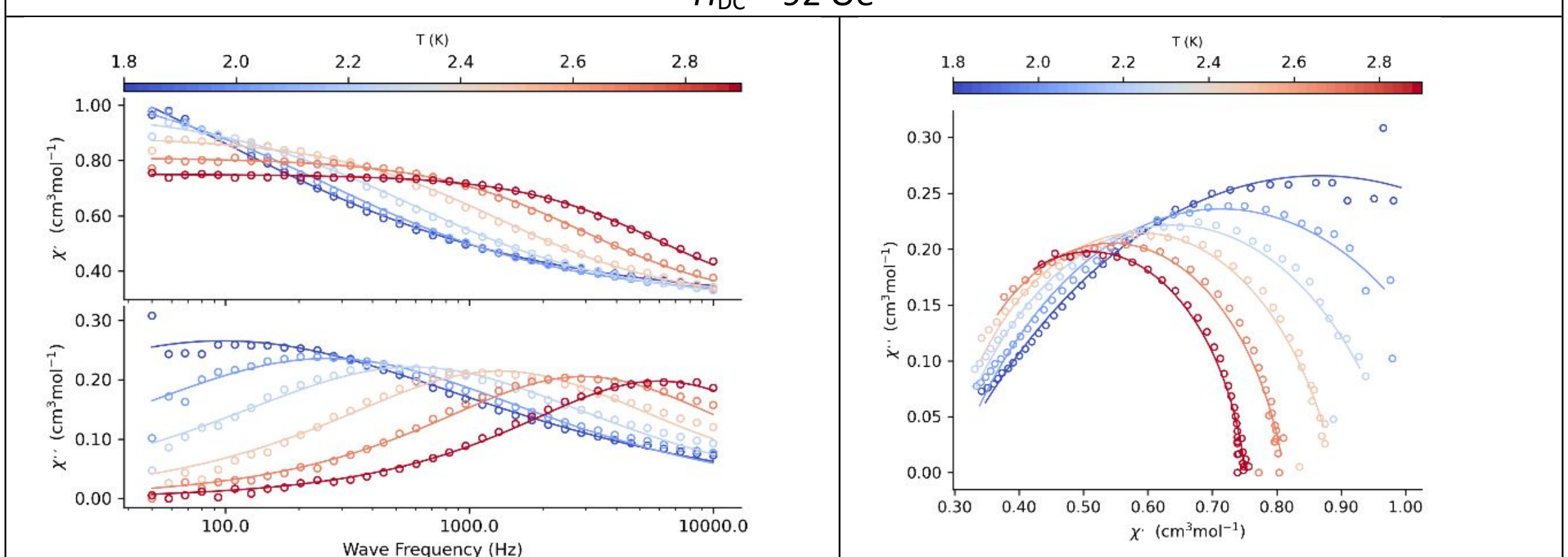

H_{DC} = 143 Oe

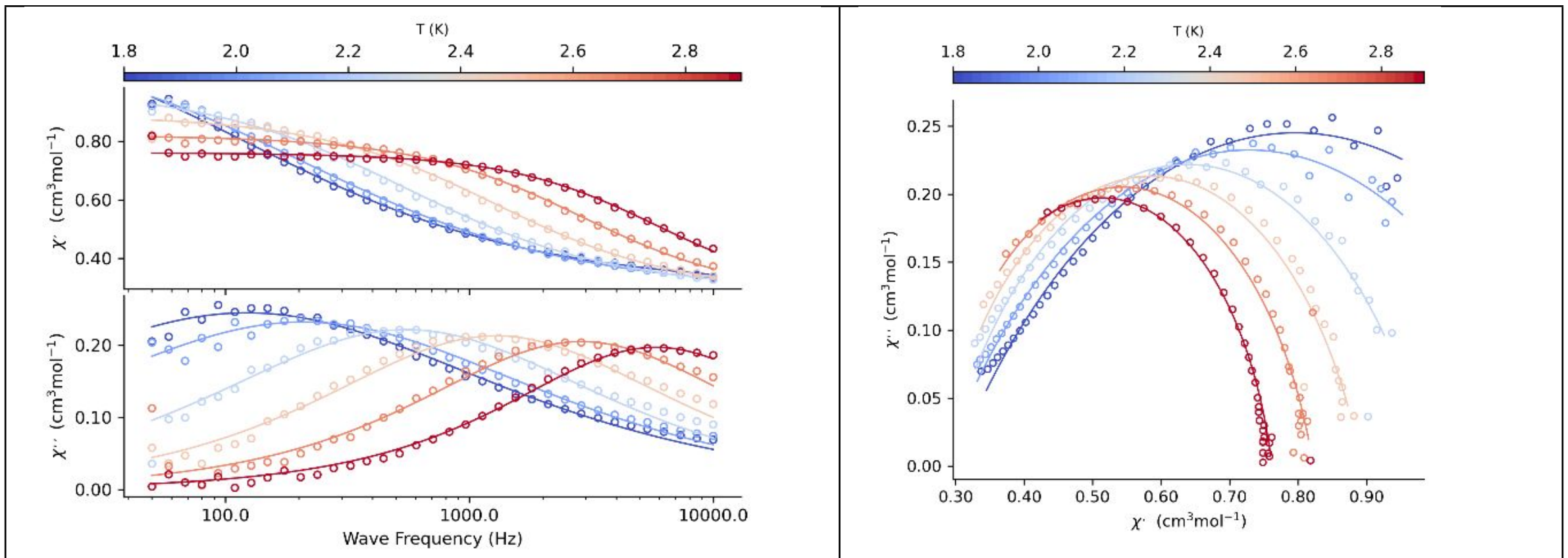

H_{DC} = 223 Oe

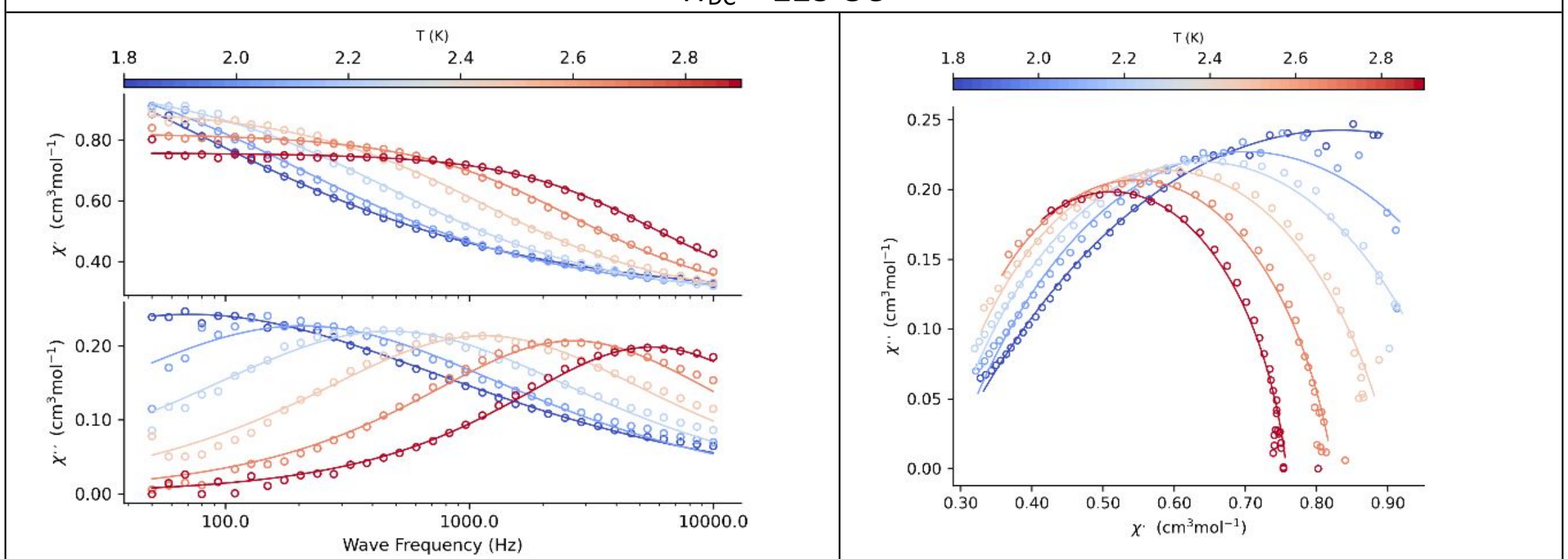

H_{DC} = 348 Oe

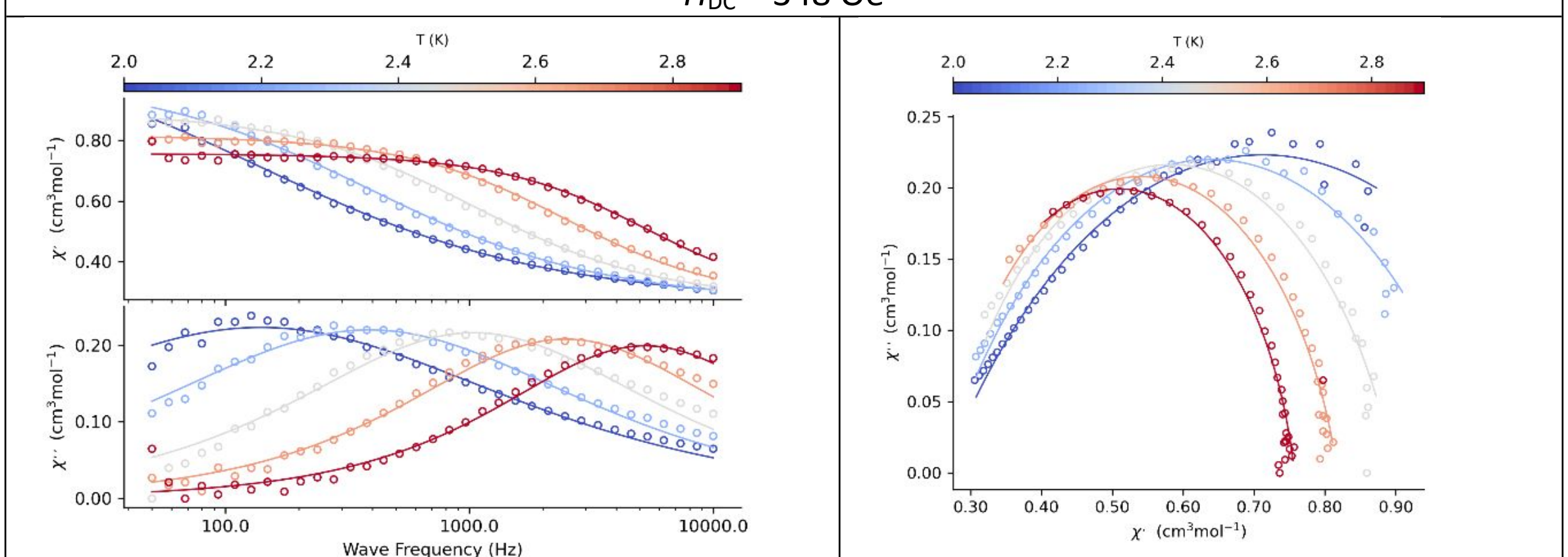

H_{DC} = 543 Oe

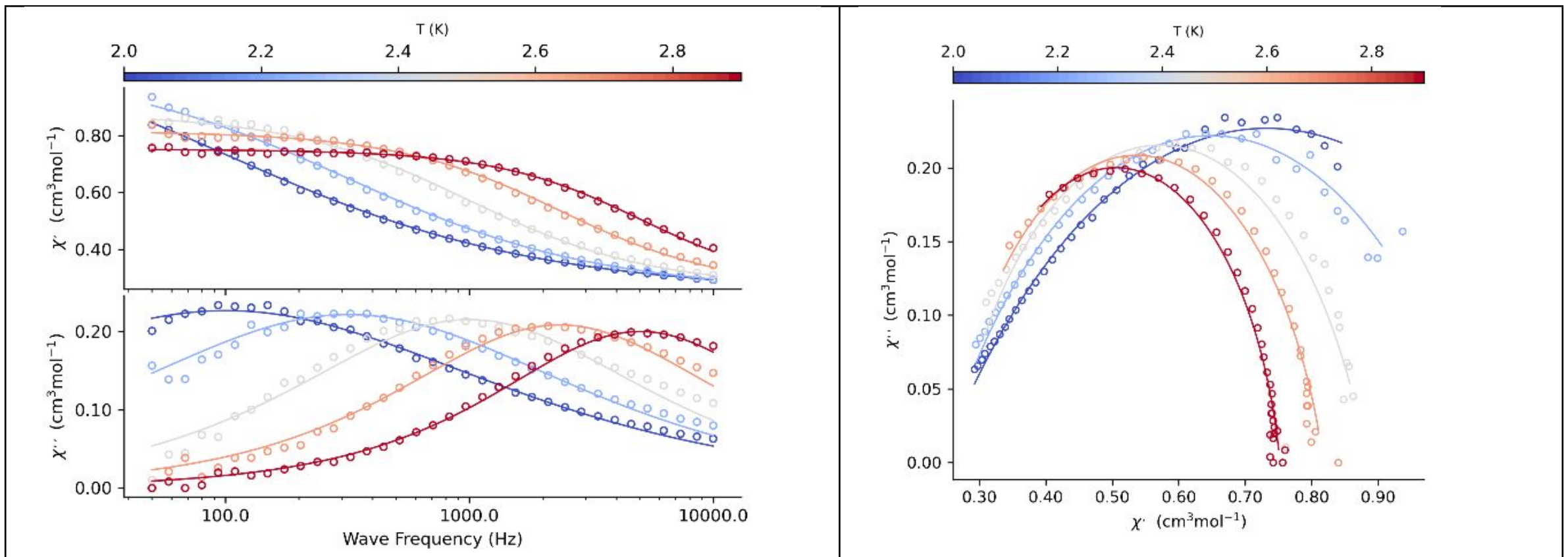

H_{DC} = 847 Oe

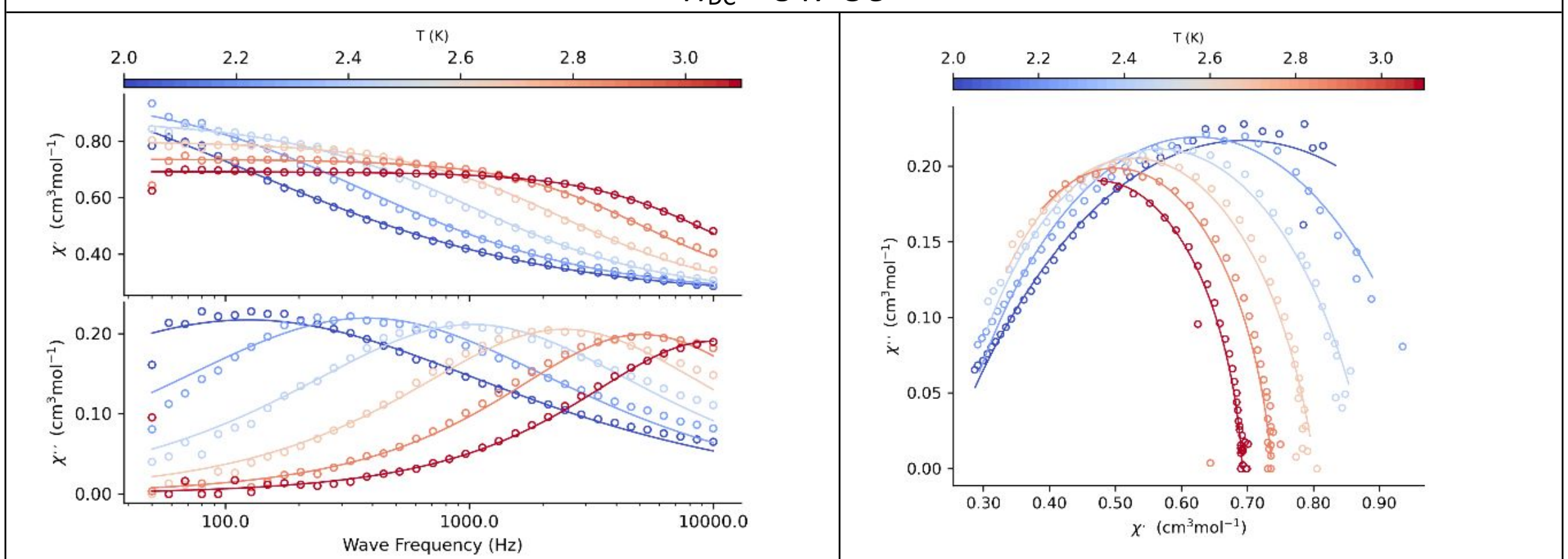

H_{DC} = 1320 Oe

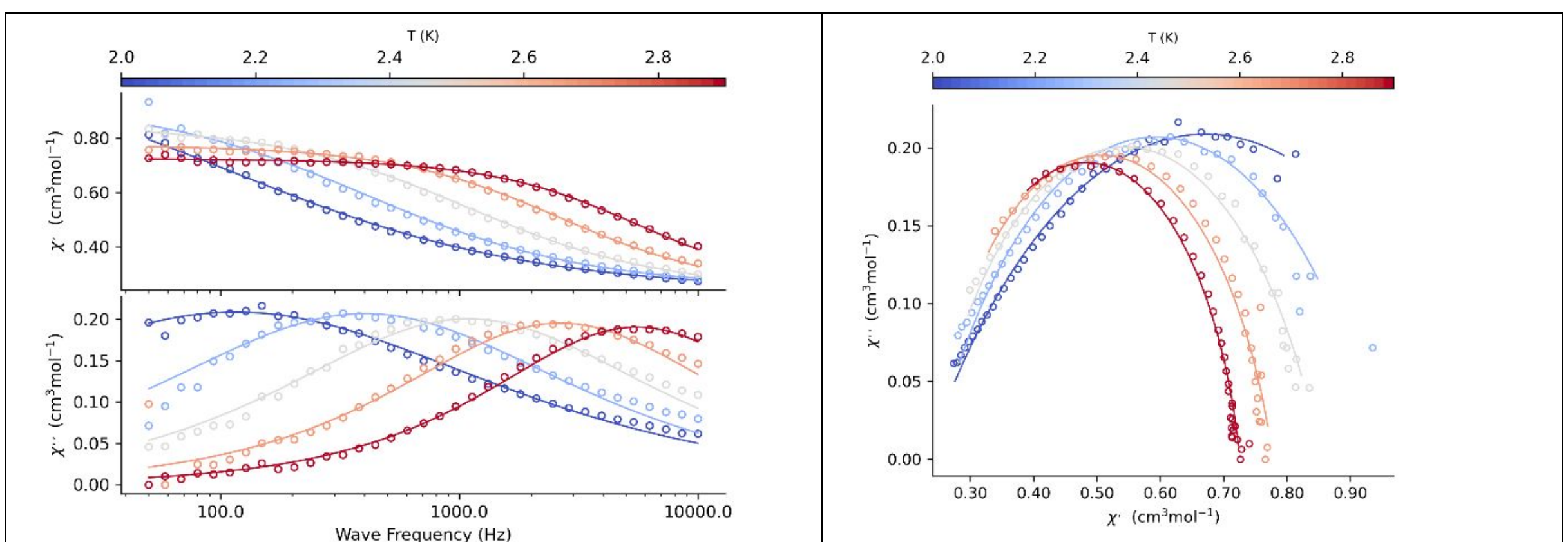

H_{DC} = 2057 Oe

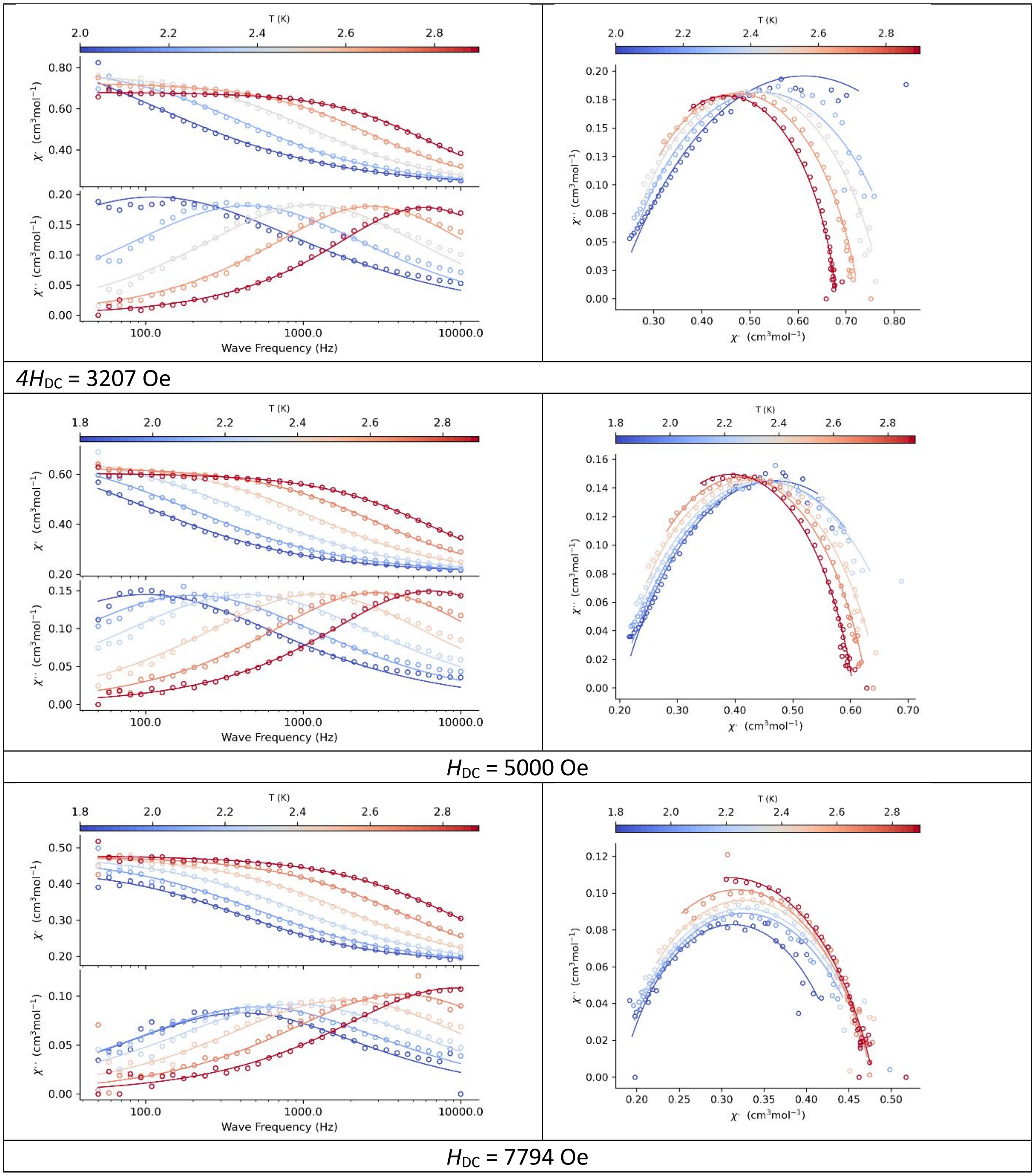

Figure S14. Frequency dependence of the in-phase (χ') and out-of-phase ((χ'') AC magnetic susceptibility measured under 20 different applied DC magnetic fields (H_{DC}).

The plots illustrate the evolution of both χ' and χ'' components with increasing DC field, highlighting field-dependent shifts in relaxation dynamics and peak positions across the investigated frequency range.

Table S16. Summary of the extracted magnetic relaxation parameters, including the relaxation times (τ), isothermal (χ_T) and adiabatic (χ_S) susceptibilities, and the Cole-Cole distribution parameter (α), together with their associated estimated uncertainties, derived from fitting the AC susceptibility data.

H (Oe)	*T* (K)	□τ(s^{-1})	$\tau_{err.}$ (s^{-1})	□χ_S (cm^3 mol^{-1})	χ_S err (cm^3 mol^{-1})	χ_T (cm^3 mol^{-1})	χ_T err (cm^3 mol^{-1})	α	□α err.
0	1.8	1.02E-03	6.62E-05	3.12E-01	7.88E-03	1.35E+00	2.55E-02	4.16E-01	1.48E-02
0	2	5.48E-04	2.59E-05	3.04E-01	8.47E-03	1.16E+00	1.68E-02	3.61E-01	1.62E-02
0	2.2	2.49E-04	5.93E-06	3.00E-01	6.01E-03	1.01E+00	6.49E-03	2.86E-01	1.10E-02
0	2.4	1.11E-04	3.97E-06	2.81E-01	1.04E-02	9.18E-01	6.15E-03	2.51E-01	1.63E-02
0	2.6	5.17E-05	2.06E-06	2.84E-01	1.21E-02	8.29E-01	3.94E-03	1.76E-01	1.73E-02
0	2.8	2.62E-05	8.25E-07	2.84E-01	9.50E-03	7.69E-01	1.63E-03	1.28E-01	1.13E-02
2.6	1.8	1.05E-03	6.34E-05	3.11E-01	7.24E-03	1.31E+00	2.43E-02	3.77E-01	1.52E-02
2.6	2	5.48E-04	1.82E-05	2.97E-01	5.83E-03	1.11E+00	1.15E-02	3.34E-01	1.21E-02
2.6	2.2	2.62E-04	5.20E-06	2.84E-01	4.84E-03	9.88E-01	5.42E-03	2.86E-01	9.18E-03
2.6	2.4	1.14E-04	3.01E-06	2.80E-01	7.43E-03	8.79E-01	4.52E-03	2.17E-01	1.29E-02
2.6	2.6	5.33E-05	1.07E-06	2.72E-01	5.98E-03	8.08E-01	2.01E-03	1.74E-01	8.83E-03
2.6	2.8	2.65E-05	7.83E-07	2.69E-01	8.77E-03	7.47E-01	1.52E-03	1.31E-01	1.06E-02
4.1	1.8	1.12E-03	3.98E-05	3.04E-01	4.05E-03	1.33E+00	1.43E-02	3.96E-01	8.26E-03
4.1	2	5.94E-04	1.61E-05	2.94E-01	4.58E-03	1.14E+00	9.68E-03	3.48E-01	9.29E-03
4.1	2.2	2.59E-04	6.83E-06	2.89E-01	6.43E-03	9.84E-01	7.13E-03	2.74E-01	1.24E-02
4.1	2.4	1.15E-04	2.34E-06	2.73E-01	5.76E-03	8.88E-01	3.51E-03	2.36E-01	9.63E-03
4.1	2.6	5.32E-05	1.41E-06	2.69E-01	7.92E-03	8.11E-01	2.65E-03	1.80E-01	1.15E-02
4.1	2.8	2.64E-05	1.07E-06	2.68E-01	1.21E-02	7.46E-01	2.10E-03	1.27E-01	1.46E-02
6.4	1.8	1.18E-03	5.59E-05	2.98E-01	5.15E-03	1.35E+00	1.89E-02	4.12E-01	1.02E-02
6.4	2	6.64E-04	2.99E-05	2.84E-01	7.16E-03	1.19E+00	1.65E-02	3.81E-01	1.37E-02
6.4	2.2	2.55E-04	5.47E-06	2.88E-01	5.24E-03	9.80E-01	5.75E-03	2.75E-01	1.01E-02
6.4	2.4	1.15E-04	2.67E-06	2.78E-01	6.55E-03	8.84E-01	4.00E-03	2.23E-01	1.12E-02
6.4	2.6	5.30E-05	1.50E-06	2.67E-01	8.44E-03	8.13E-01	2.80E-03	1.86E-01	1.21E-02
6.4	2.8	2.70E-05	1.36E-06	2.77E-01	1.50E-02	7.44E-01	2.68E-03	1.13E-01	1.90E-02
10	1.8	1.23E-03	4.73E-05	3.01E-01	4.11E-03	1.36E+00	1.56E-02	4.08E-01	8.25E-03
10	2	5.79E-04	1.55E-05	2.95E-01	4.59E-03	1.13E+00	9.51E-03	3.45E-01	9.38E-03
10	2.2	2.63E-04	5.42E-06	2.84E-01	5.00E-03	9.87E-01	5.61E-03	2.87E-01	9.51E-03
10	2.4	1.16E-04	2.21E-06	2.77E-01	5.36E-03	8.85E-01	3.30E-03	2.26E-01	9.19E-03
10	2.6	5.31E-05	1.09E-06	2.69E-01	6.08E-03	8.09E-01	2.03E-03	1.80E-01	8.86E-03
10	2.8	2.61E-05	1.62E-06	2.64E-01	1.84E-02	7.44E-01	3.14E-03	1.32E-01	2.19E-02
15	1.8	1.06E-03	4.78E-05	3.06E-01	5.23E-03	1.30E+00	1.77E-02	3.89E-01	1.08E-02
15	2	5.96E-04	1.50E-05	2.93E-01	4.24E-03	1.14E+00	8.98E-03	3.51E-01	8.59E-03
15	2.2	2.61E-04	5.09E-06	2.84E-01	4.74E-03	9.86E-01	5.30E-03	2.86E-01	9.02E-03
15	2.4	1.14E-04	3.20E-06	2.79E-01	7.87E-03	8.75E-01	4.78E-03	2.16E-01	1.38E-02
15	2.6	5.33E-05	1.99E-06	2.73E-01	1.11E-02	8.03E-01	3.72E-03	1.70E-01	1.66E-02
15	2.8	2.65E-05	1.14E-06	2.70E-01	1.29E-02	7.42E-01	2.24E-03	1.21E-01	1.58E-02
24	1.8	1.13E-03	4.43E-05	3.04E-01	4.42E-03	1.33E+00	1.57E-02	3.98E-01	9.00E-03

24	2	5.98E-04	2.19E-05	2.96E-01	6.21E-03	1.14E+00	1.32E-02	3.45E-01	1.27E-02
24	2.2	2.61E-04	6.50E-06	2.87E-01	6.04E-03	9.85E-01	6.75E-03	2.80E-01	1.16E-02
24	2.4	1.12E-04	4.31E-06	2.82E-01	1.07E-02	8.69E-01	6.46E-03	2.06E-01	1.91E-02
24	2.6	5.36E-05	1.46E-06	2.71E-01	8.09E-03	8.10E-01	2.73E-03	1.78E-01	1.19E-02
24	2.8	2.71E-05	7.03E-07	2.77E-01	7.71E-03	7.44E-01	1.38E-03	1.17E-01	9.71E-03
37	1.8	1.04E-03	6.53E-05	3.13E-01	7.51E-03	1.30E+00	2.52E-02	3.75E-01	1.59E-02
37	2	5.89E-04	1.80E-05	2.95E-01	5.19E-03	1.14E+00	1.09E-02	3.45E-01	1.06E-02
37	2.2	2.65E-04	7.51E-06	2.78E-01	6.90E-03	9.94E-01	7.80E-03	3.02E-01	1.28E-02
37	2.4	1.16E-04	2.84E-06	2.81E-01	6.88E-03	8.81E-01	4.24E-03	2.18E-01	1.20E-02
37	2.6	5.31E-05	1.84E-06	2.75E-01	1.03E-02	8.01E-01	3.49E-03	1.61E-01	1.57E-02
37	2.8	2.68E-05	6.34E-07	2.73E-01	7.05E-03	7.44E-01	1.24E-03	1.20E-01	8.73E-03
59	1.8	1.04E-03	4.93E-05	3.09E-01	5.61E-03	1.30E+00	1.87E-02	3.82E-01	1.18E-02
59	2	5.71E-04	2.24E-05	2.97E-01	6.76E-03	1.12E+00	1.38E-02	3.40E-01	1.40E-02
59	2.2	2.64E-04	7.72E-06	2.89E-01	7.09E-03	9.89E-01	7.99E-03	2.77E-01	1.37E-02
59	2.4	1.16E-04	2.15E-06	2.75E-01	5.22E-03	8.88E-01	3.21E-03	2.31E-01	8.83E-03
59	2.6	5.39E-05	1.20E-06	2.76E-01	6.64E-03	8.08E-01	2.26E-03	1.68E-01	9.98E-03
59	2.8	2.66E-05	8.79E-07	2.69E-01	9.80E-03	7.49E-01	1.71E-03	1.32E-01	1.18E-02
92	1.8	1.09E-03	6.76E-05	3.10E-01	7.12E-03	1.32E+00	2.48E-02	3.83E-01	1.50E-02
92	2	5.85E-04	1.63E-05	2.97E-01	4.75E-03	1.13E+00	9.91E-03	3.42E-01	9.79E-03
92	2.2	2.65E-04	6.20E-06	2.85E-01	5.67E-03	9.89E-01	6.40E-03	2.85E-01	1.08E-02
92	2.4	1.16E-04	2.34E-06	2.78E-01	5.68E-03	8.88E-01	3.50E-03	2.26E-01	9.71E-03
92	2.6	5.40E-05	1.33E-06	2.76E-01	7.33E-03	8.08E-01	2.50E-03	1.66E-01	1.10E-02
92	2.8	2.65E-05	1.50E-06	2.69E-01	1.70E-02	7.48E-01	2.95E-03	1.27E-01	2.05E-02
143	1.8	1.63E-03	1.25E-04	2.88E-01	6.64E-03	1.44E+00	3.08E-02	4.52E-01	1.27E-02
143	2	6.04E-04	2.19E-05	2.96E-01	5.98E-03	1.13E+00	1.28E-02	3.44E-01	1.25E-02
143	2.2	2.71E-04	6.94E-06	2.85E-01	6.16E-03	9.92E-01	7.08E-03	2.87E-01	1.18E-02
143	2.4	1.19E-04	3.04E-06	2.83E-01	7.21E-03	8.91E-01	4.54E-03	2.17E-01	1.26E-02
143	2.6	5.43E-05	1.34E-06	2.76E-01	7.38E-03	8.12E-01	2.53E-03	1.67E-01	1.11E-02
143	2.8	2.66E-05	6.27E-07	2.68E-01	7.11E-03	7.51E-01	1.24E-03	1.27E-01	8.53E-03
223	1.8	1.30E-03	6.40E-05	2.96E-01	4.65E-03	1.30E+00	1.83E-02	4.20E-01	9.96E-03
223	2	7.59E-04	2.67E-05	2.80E-01	4.92E-03	1.18E+00	1.26E-02	3.92E-01	9.85E-03
223	2.2	2.87E-04	7.57E-06	2.85E-01	6.19E-03	9.91E-01	7.43E-03	2.87E-01	1.21E-02
223	2.4	1.24E-04	3.44E-06	2.76E-01	7.82E-03	8.93E-01	5.03E-03	2.30E-01	1.35E-02
223	2.6	5.55E-05	2.42E-06	2.64E-01	1.31E-02	8.22E-01	4.51E-03	1.93E-01	1.86E-02
223	2.8	2.73E-05	1.24E-06	2.65E-01	1.35E-02	7.62E-01	2.42E-03	1.45E-01	1.59E-02
348	1.8	2.23E-03	1.04E-04	2.75E-01	2.91E-03	1.39E+00	1.70E-02	4.78E-01	6.22E-03
348	2	7.71E-04	3.57E-05	2.84E-01	6.13E-03	1.12E+00	1.60E-02	3.64E-01	1.38E-02
348	2.2	3.38E-04	6.85E-06	2.73E-01	4.47E-03	1.01E+00	6.07E-03	3.14E-01	8.71E-03
348	2.4	1.39E-04	2.80E-06	2.67E-01	5.65E-03	9.09E-01	3.95E-03	2.52E-01	9.59E-03
348	2.6	5.97E-05	1.33E-06	2.67E-01	6.71E-03	8.23E-01	2.47E-03	1.86E-01	9.95E-03
348	2.8	2.86E-05	1.13E-06	2.67E-01	1.20E-02	7.58E-01	2.25E-03	1.35E-01	1.46E-02
543	2	1.14E-03	5.37E-05	2.62E-01	4.53E-03	1.16E+00	1.61E-02	4.14E-01	1.03E-02
543	2.2	4.10E-04	9.68E-06	2.60E-01	4.73E-03	1.02E+00	7.46E-03	3.31E-01	9.44E-03

543	2.4	1.53E-04	3.92E-06	2.66E-01	6.99E-03	8.99E-01	5.27E-03	2.36E-01	1.26E-02
543	2.6	6.42E-05	1.37E-06	2.63E-01	6.37E-03	8.18E-01	2.50E-03	1.81E-01	9.80E-03
543	2.8	3.03E-05	1.49E-06	2.63E-01	1.49E-02	7.57E-01	2.98E-03	1.36E-01	1.86E-02
847	2	1.58E-03	6.45E-05	2.42E-01	3.09E-03	1.22E+00	1.40E-02	4.49E-01	6.92E-03
847	2.2	4.97E-04	1.30E-05	2.43E-01	4.75E-03	1.05E+00	8.73E-03	3.62E-01	9.25E-03
847	2.4	1.59E-04	5.99E-06	2.60E-01	1.00E-02	8.84E-01	7.80E-03	2.30E-01	1.88E-02
847	2.6	6.78E-05	1.67E-06	2.54E-01	7.35E-03	8.18E-01	3.00E-03	1.89E-01	1.13E-02
847	2.8	3.17E-05	7.24E-07	2.59E-01	6.93E-03	7.53E-01	1.46E-03	1.33E-01	8.91E-03
1320	2	1.28E-03	9.49E-05	2.38E-01	6.36E-03	1.15E+00	2.45E-02	4.36E-01	1.44E-02
1320	2.2	4.20E-04	1.42E-05	2.50E-01	6.59E-03	9.97E-01	1.06E-02	3.24E-01	1.36E-02
1320	2.4	1.54E-04	3.28E-06	2.48E-01	5.72E-03	8.83E-01	4.33E-03	2.52E-01	1.02E-02
1320	2.6	6.53E-05	1.55E-06	2.53E-01	6.99E-03	8.02E-01	2.78E-03	1.83E-01	1.09E-02
1320	2.8	3.12E-05	1.69E-06	2.63E-01	1.64E-02	7.37E-01	3.42E-03	1.13E-01	2.20E-02
1320	3	1.60E-05	2.03E-06	2.57E-01	3.72E-02	6.93E-01	3.53E-03	8.64E-02	3.67E-02
2057	2	1.37E-03	6.48E-05	2.29E-01	3.68E-03	1.11E+00	1.50E-02	4.37E-01	8.85E-03
2057	2.2	3.96E-04	1.77E-05	2.40E-01	8.55E-03	9.46E-01	1.31E-02	3.24E-01	1.82E-02
2057	2.4	1.46E-04	2.75E-06	2.36E-01	4.93E-03	8.53E-01	3.58E-03	2.66E-01	8.75E-03
2057	2.6	6.01E-05	2.35E-06	2.36E-01	1.11E-02	7.78E-01	4.08E-03	2.04E-01	1.67E-02
2057	2.8	2.87E-05	7.01E-07	2.40E-01	7.04E-03	7.26E-01	1.32E-03	1.52E-01	8.62E-03
3207	2	1.42E-03	1.64E-04	2.19E-01	8.01E-03	1.01E+00	3.43E-02	4.13E-01	2.29E-02
3207	2.2	3.61E-04	1.23E-05	2.22E-01	5.98E-03	8.29E-01	8.55E-03	3.14E-01	1.45E-02
3207	2.4	1.40E-04	3.07E-06	2.22E-01	5.27E-03	7.77E-01	3.70E-03	2.59E-01	1.03E-02
3207	2.6	5.85E-05	1.47E-06	2.21E-01	6.60E-03	7.28E-01	2.36E-03	2.12E-01	1.04E-02
3207	2.8	2.70E-05	7.68E-07	2.23E-01	7.67E-03	6.80E-01	1.34E-03	1.57E-01	9.61E-03
5000	1.8	1.59E-03	1.08E-04	2.03E-01	3.05E-03	7.34E-01	1.48E-02	3.64E-01	1.47E-02
5000	2	7.99E-04	2.85E-05	1.99E-01	2.88E-03	7.13E-01	7.75E-03	3.50E-01	1.09E-02
5000	2.2	3.63E-04	1.35E-05	1.91E-01	5.34E-03	7.07E-01	7.69E-03	3.48E-01	1.47E-02
5000	2.4	1.31E-04	2.98E-06	1.95E-01	4.49E-03	6.52E-01	2.99E-03	2.78E-01	1.02E-02
5000	2.6	5.55E-05	1.42E-06	1.91E-01	5.55E-03	6.29E-01	1.87E-03	2.46E-01	9.59E-03
5000	2.8	2.46E-05	1.04E-06	1.81E-01	9.49E-03	6.05E-01	1.48E-03	2.18E-01	1.17E-02
7794	1.8	3.91E-04	1.62E-05	1.81E-01	3.10E-03	4.45E-01	4.70E-03	2.86E-01	1.82E-02
7794	2	3.06E-04	1.83E-05	1.75E-01	5.66E-03	4.77E-01	7.14E-03	3.21E-01	2.57E-02
7794	2.2	1.75E-04	4.98E-06	1.72E-01	3.45E-03	4.82E-01	2.85E-03	3.21E-01	1.23E-02
7794	2.4	8.58E-05	3.02E-06	1.72E-01	4.92E-03	4.81E-01	2.35E-03	2.91E-01	1.38E-02
7794	2.6	3.78E-05	4.00E-06	1.60E-01	1.61E-02	4.77E-01	3.82E-03	2.74E-01	3.14E-02
7794	2.8	1.73E-05	2.22E-06	1.42E-01	2.08E-02	4.79E-01	2.24E-03	2.73E-01	2.57E-02

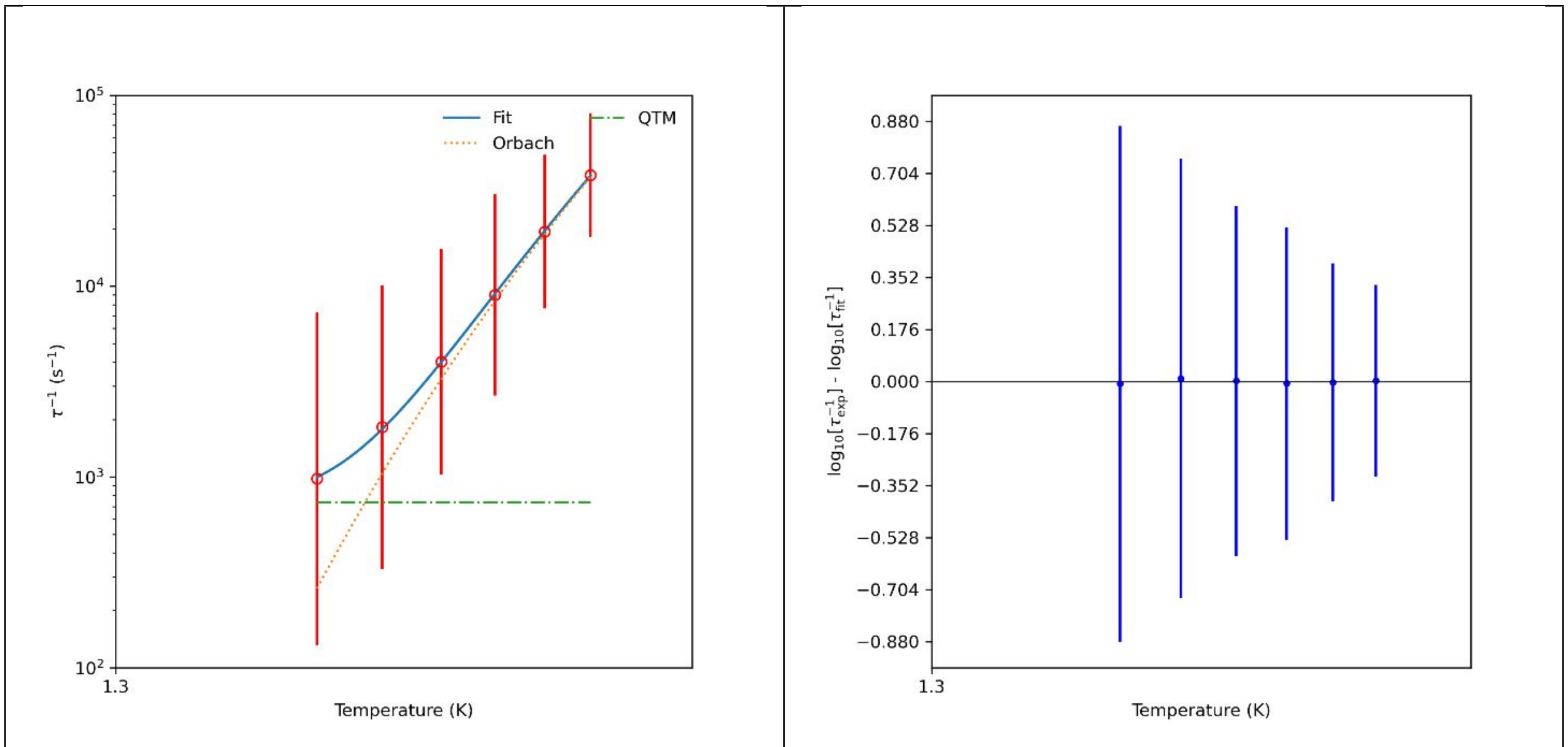

H_{DC} = 0 Oe

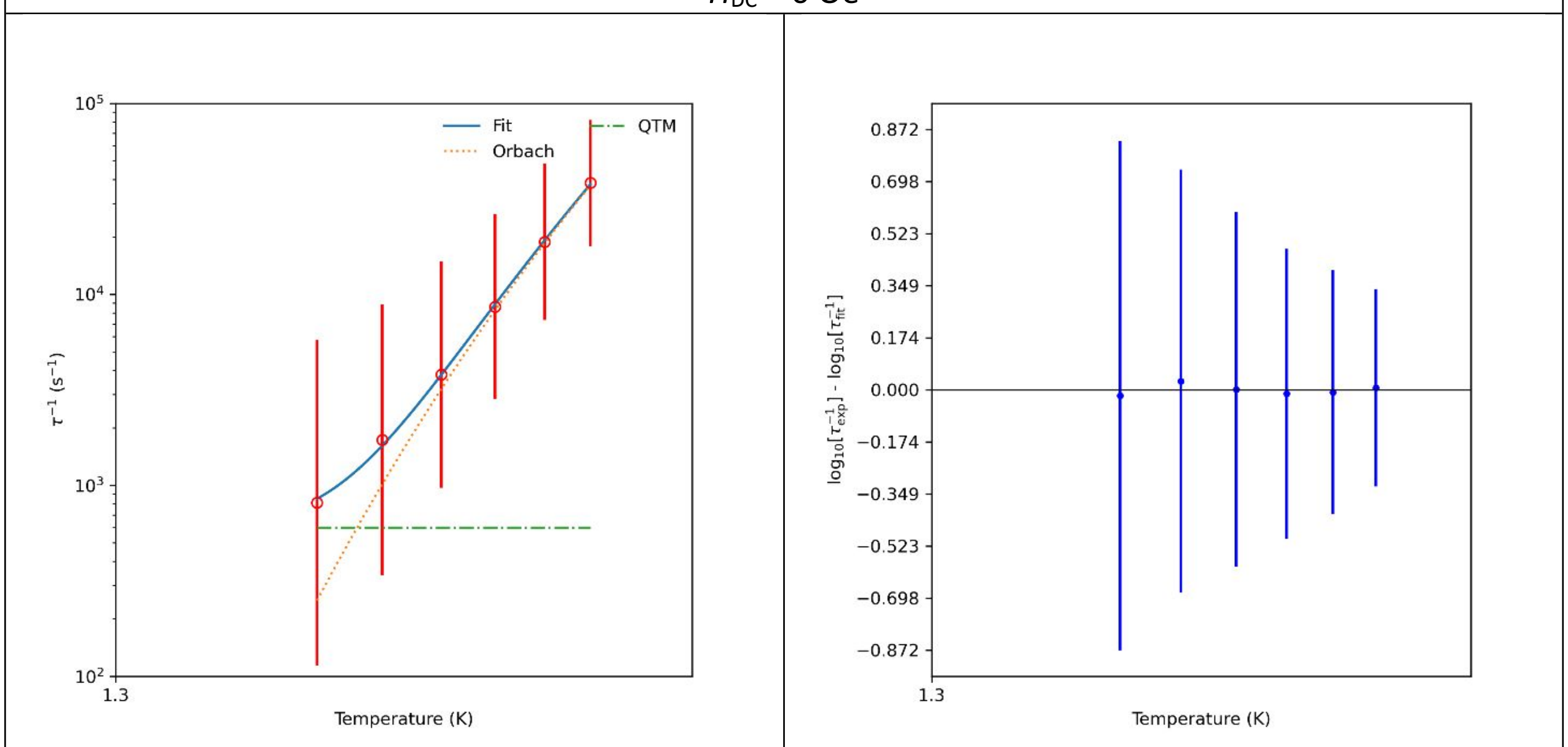

H_{DC} = 10 Oe

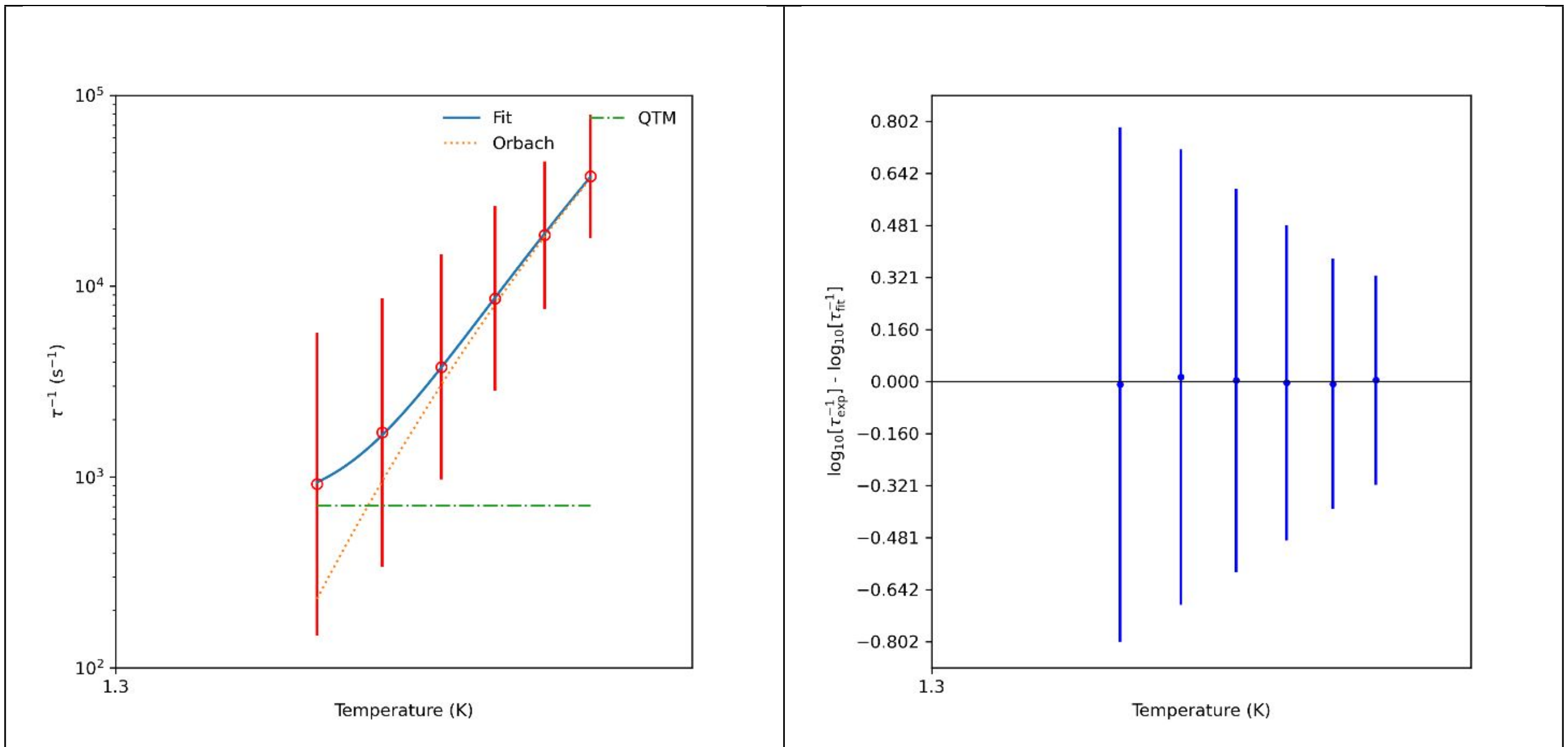

H_{DC} = 92 Oe

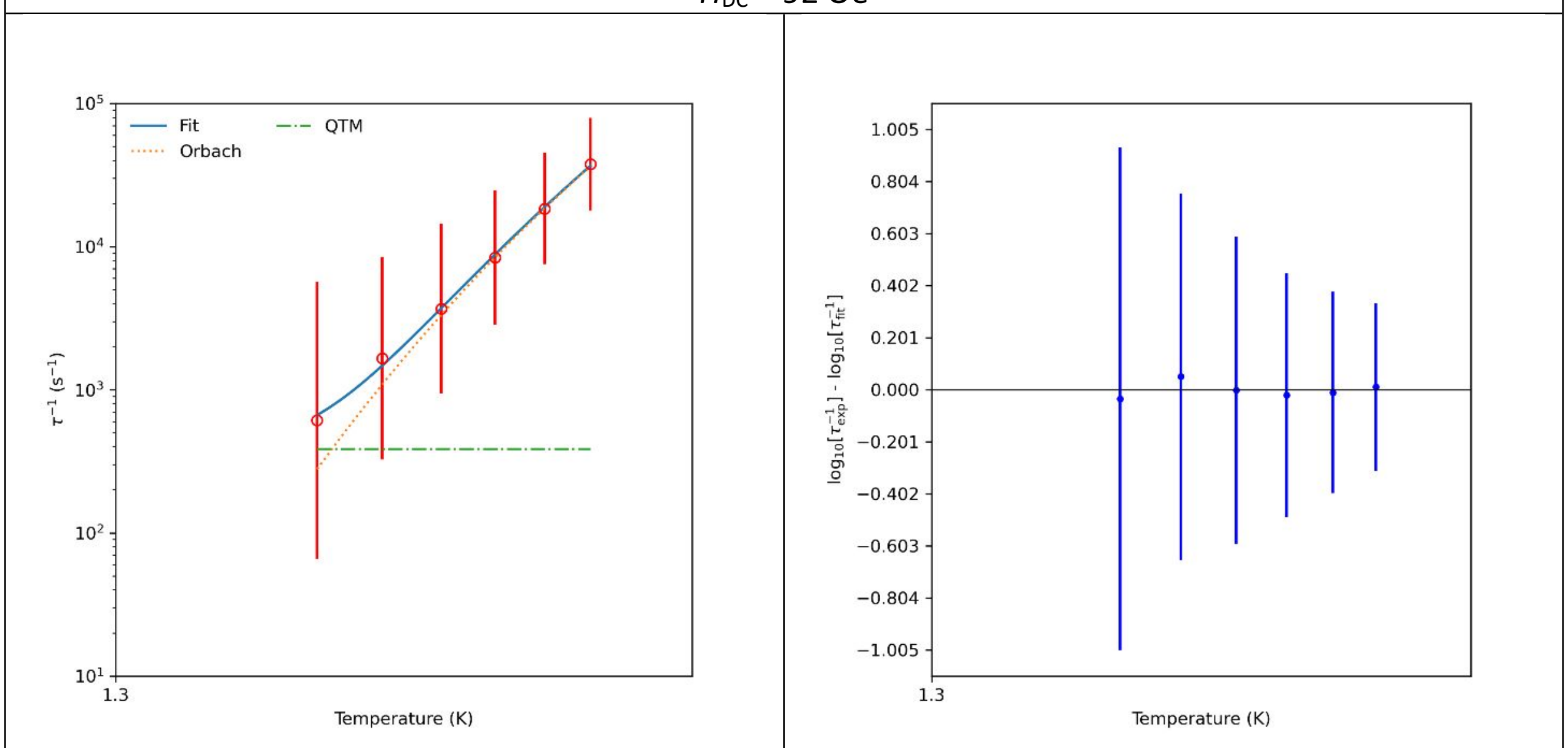

H_{DC} = 143 Oe

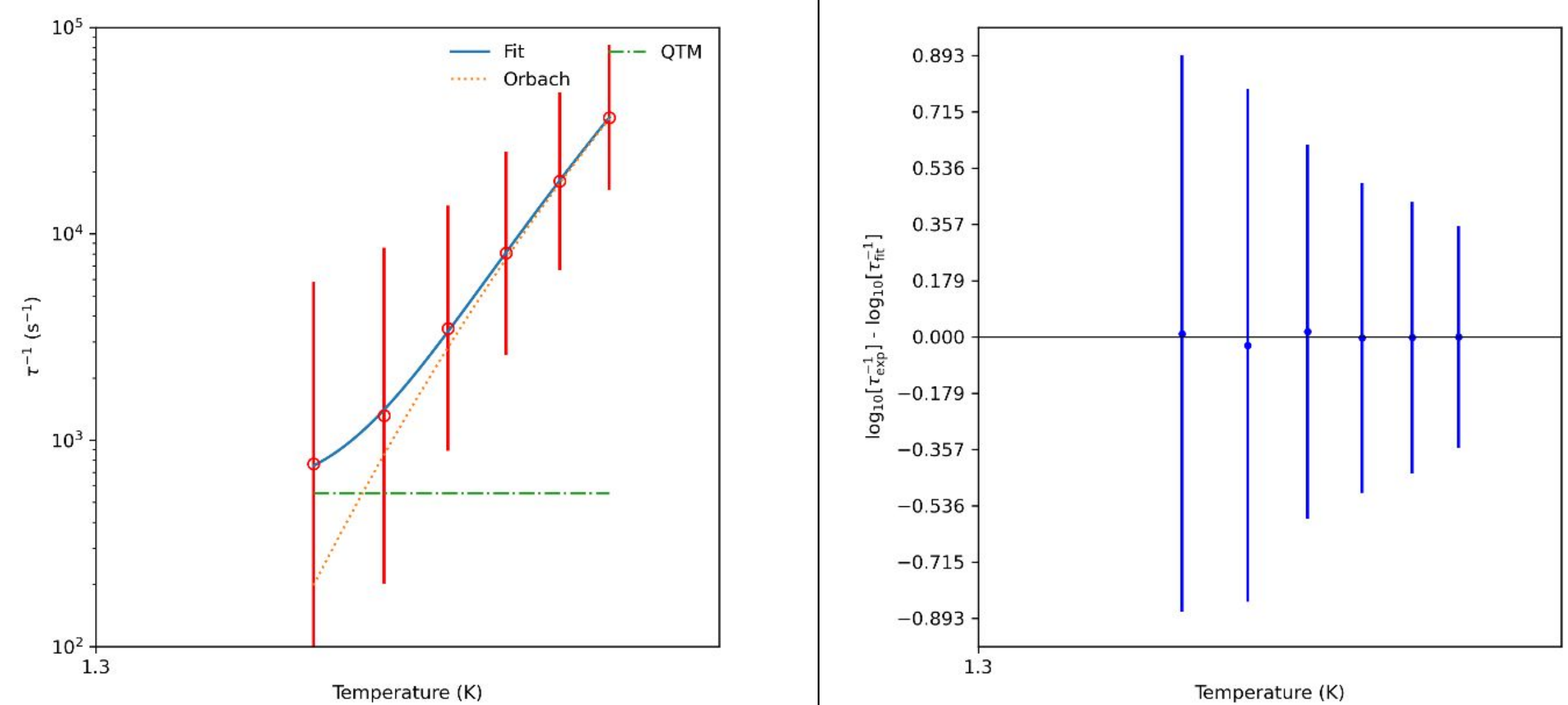

H_{DC} = 223 Oe

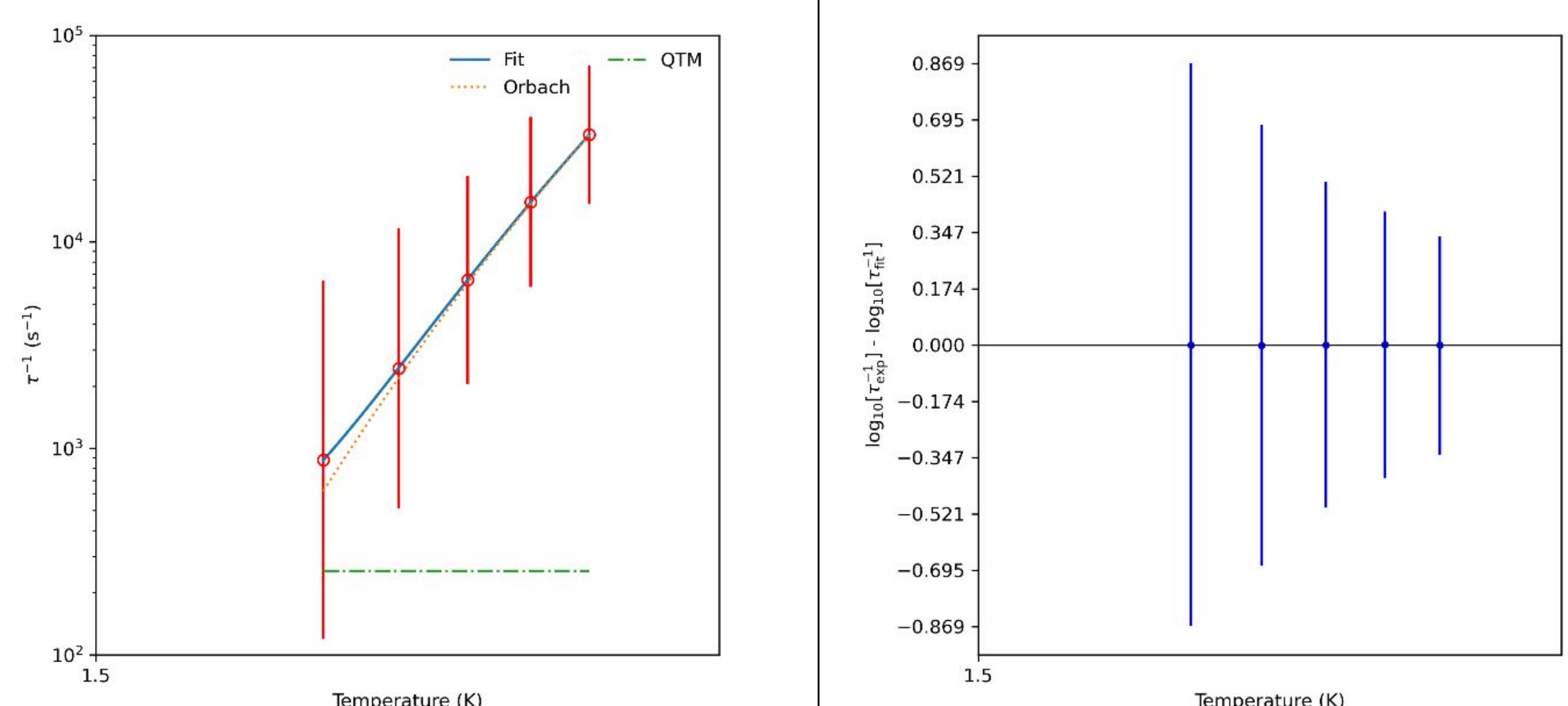

H_{DC} = 543 Oe

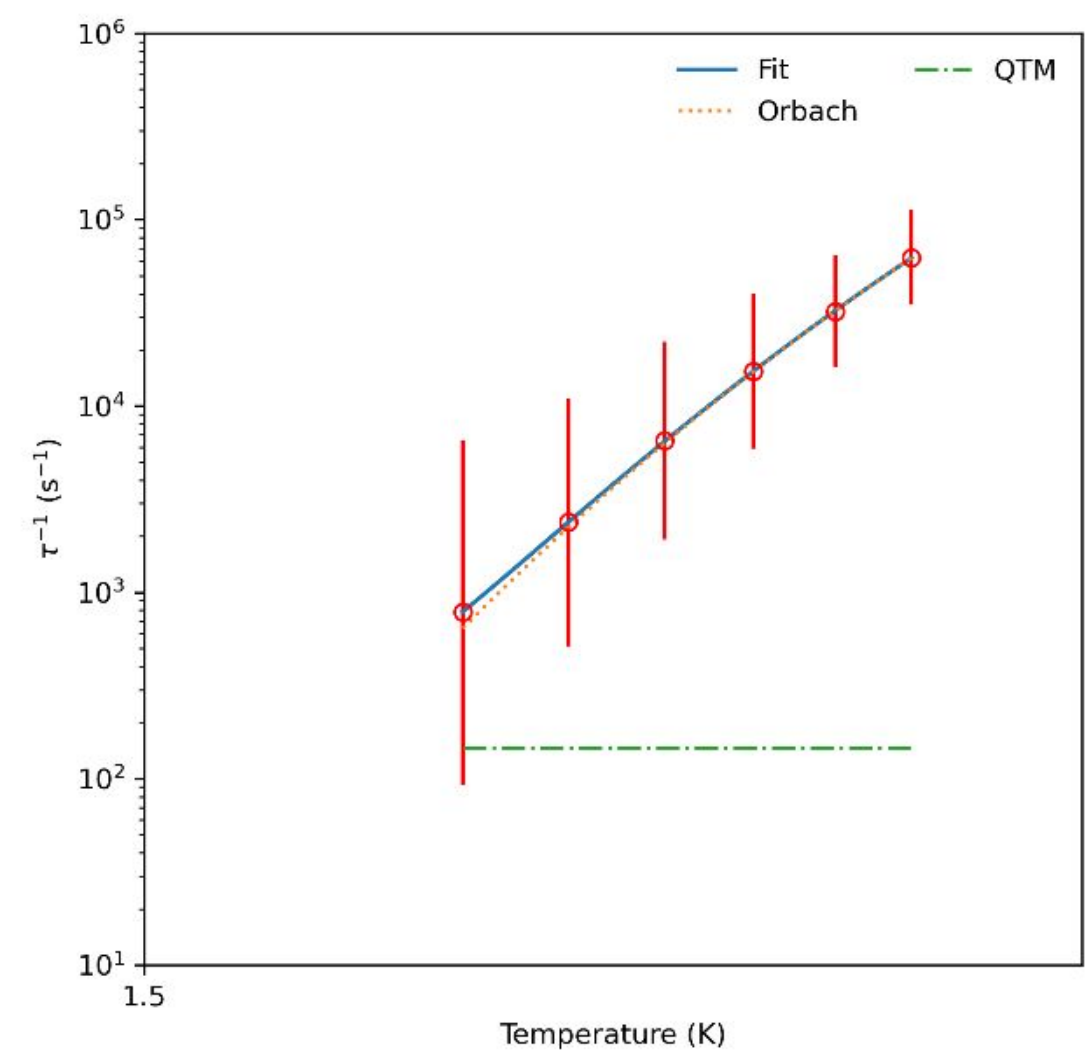

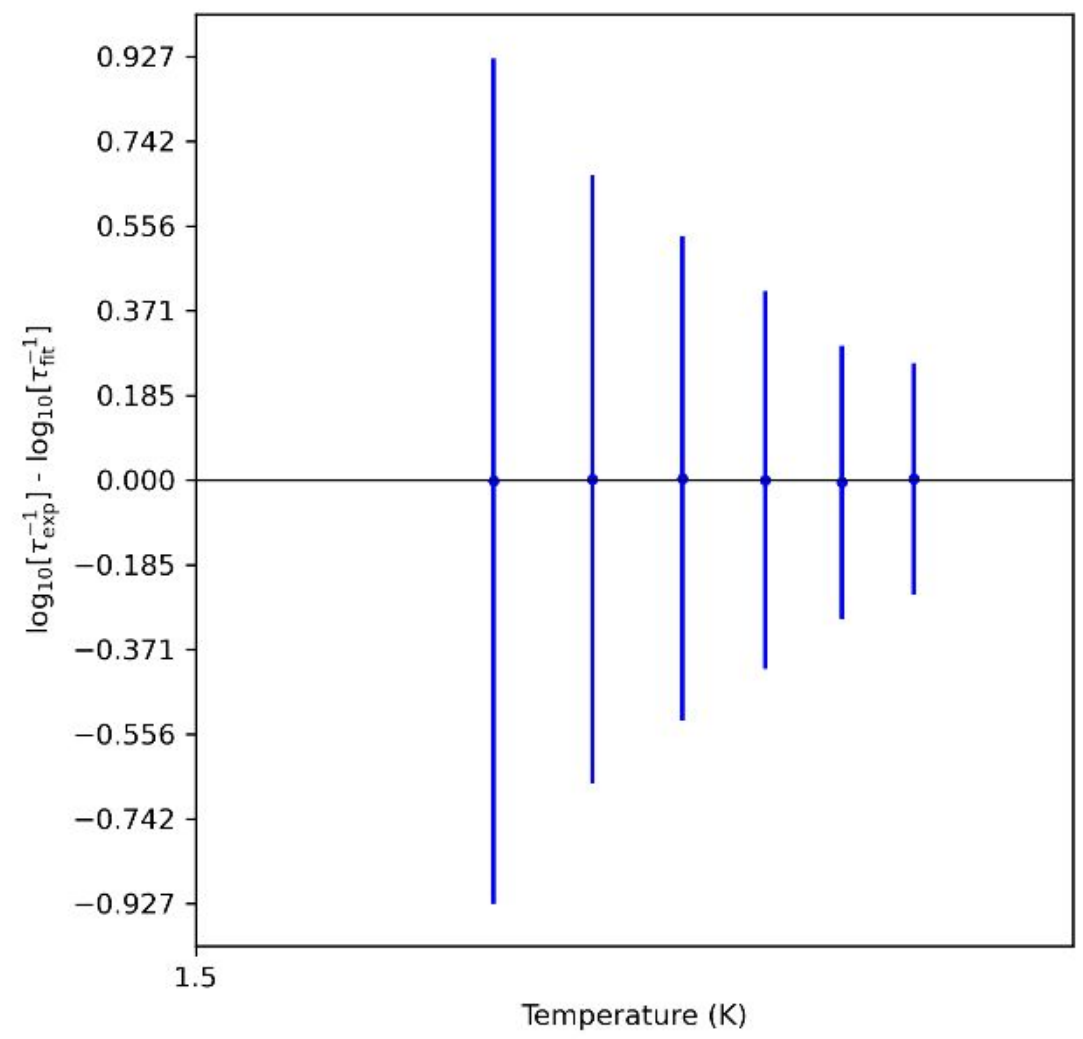

H_{DC} = 1320 Oe

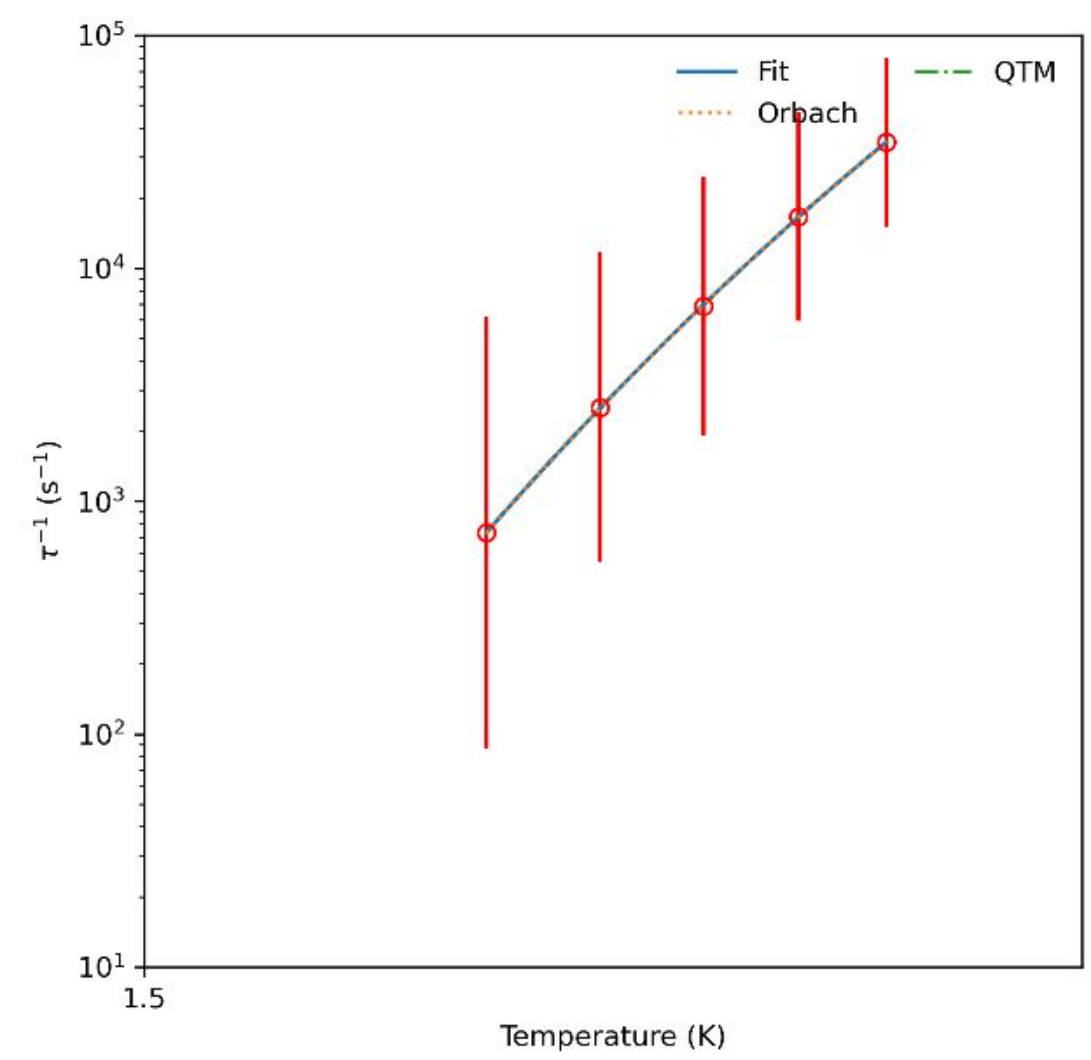

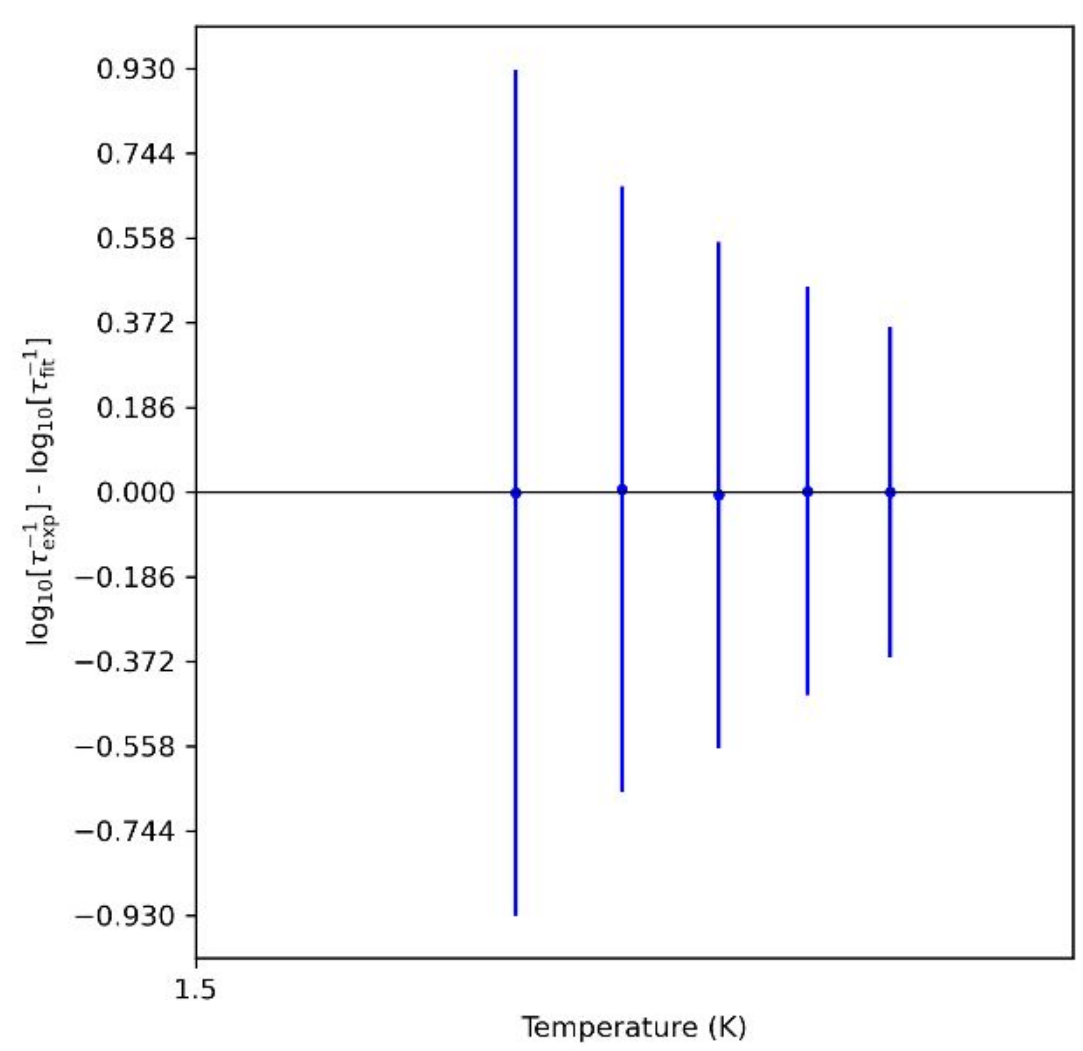

H_{DC} = 2057 Oe

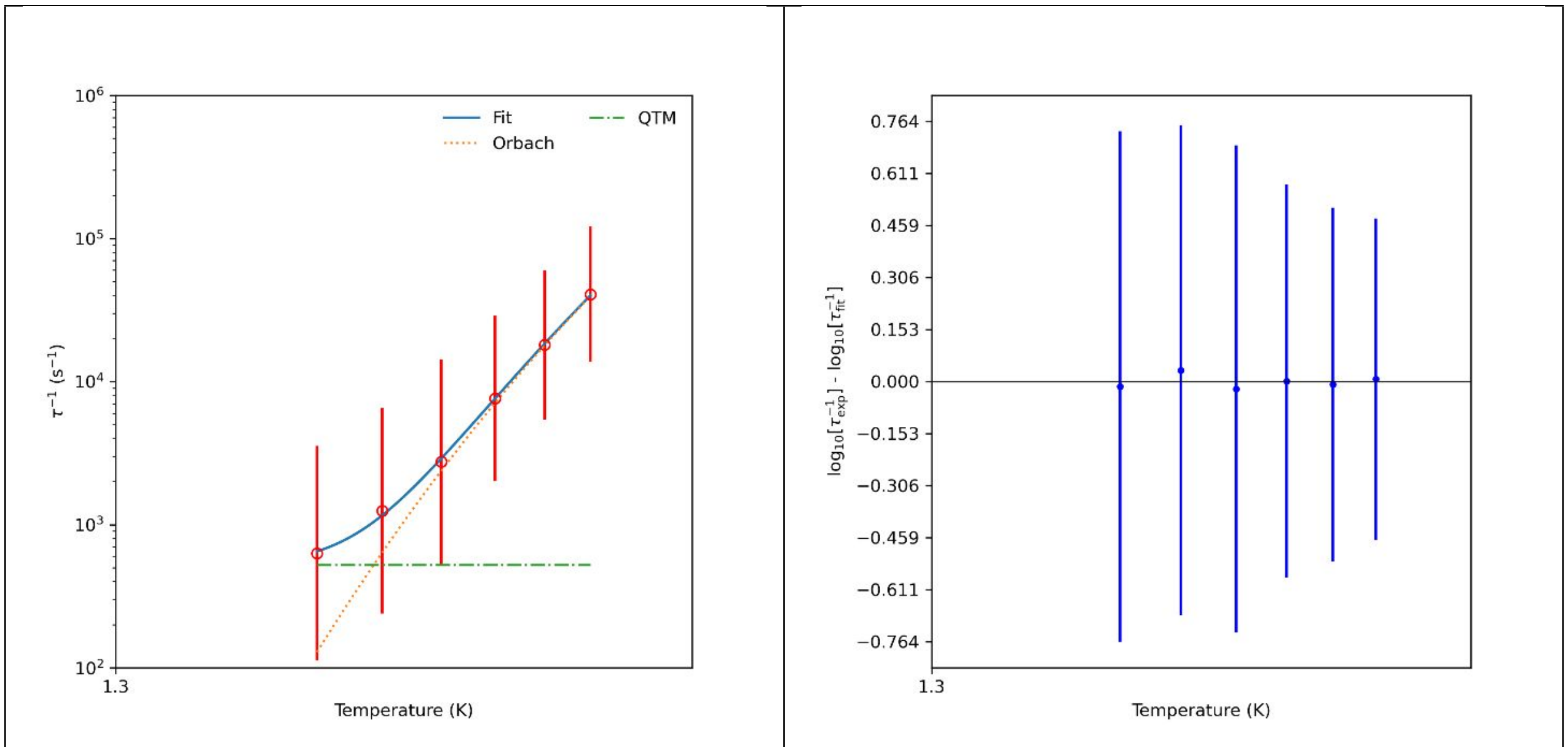

H_{DC} = 5000 Oe

Figure S15. Temperature dependence of the relaxation rate (τ^{-1}) with corresponding fits using a combined orbach and quantum tunneling model, and residuals of the fit shown as $\log_{10}(\tau_{exp})$-$\log_{10}(\tau_{fit})$ versus temperature for different applied DC magnetic fields.

Table S17. Extracted relaxation parameters, including the Orbach pre-factor (τ_0), effective energy barrier (U_{eff}), and quantum tunneling rate (τ_{QTM}), with associated uncertainties.

**Field (Oe)**	**$\log_{10}[\tau_0]$ ($\log_{10}$ [s])**	**$\log_{10}[\tau_0]$ err. ($\log_{10}$ [s])**	**U_{eff} (K)**	**U_{eff} err. (K)**	**$\log_{10}[\tau_{QTM}]$ ($\log_{10}$ [s])**	**$\log_{10}[\tau_{QTM}]$ err. ($\log_{10}$ [s])**
0	-8.45	3.07	25.01	18.68	-2.87	1.40
10	-8.48	2.93	25.21	17.69	-2.78	1.46
92	-8.53	2.97	25.56	18.00	-2.85	1.24
143	-8.37	2.74	24.55	16.54	-2.59	2.13
223	-8.63	3.03	26.23	18.26	-2.74	1.40
543	-8.82	3.93	27.77	24.19	-2.41	5.14
1320	-8.77	2.34	27.45	15.26	-2.16	7.53
2057	-8.75	3.92	27.10	24.08	-0.86	187.82
5000	-9.07	3.54	28.86	21.00	-2.72	1.09

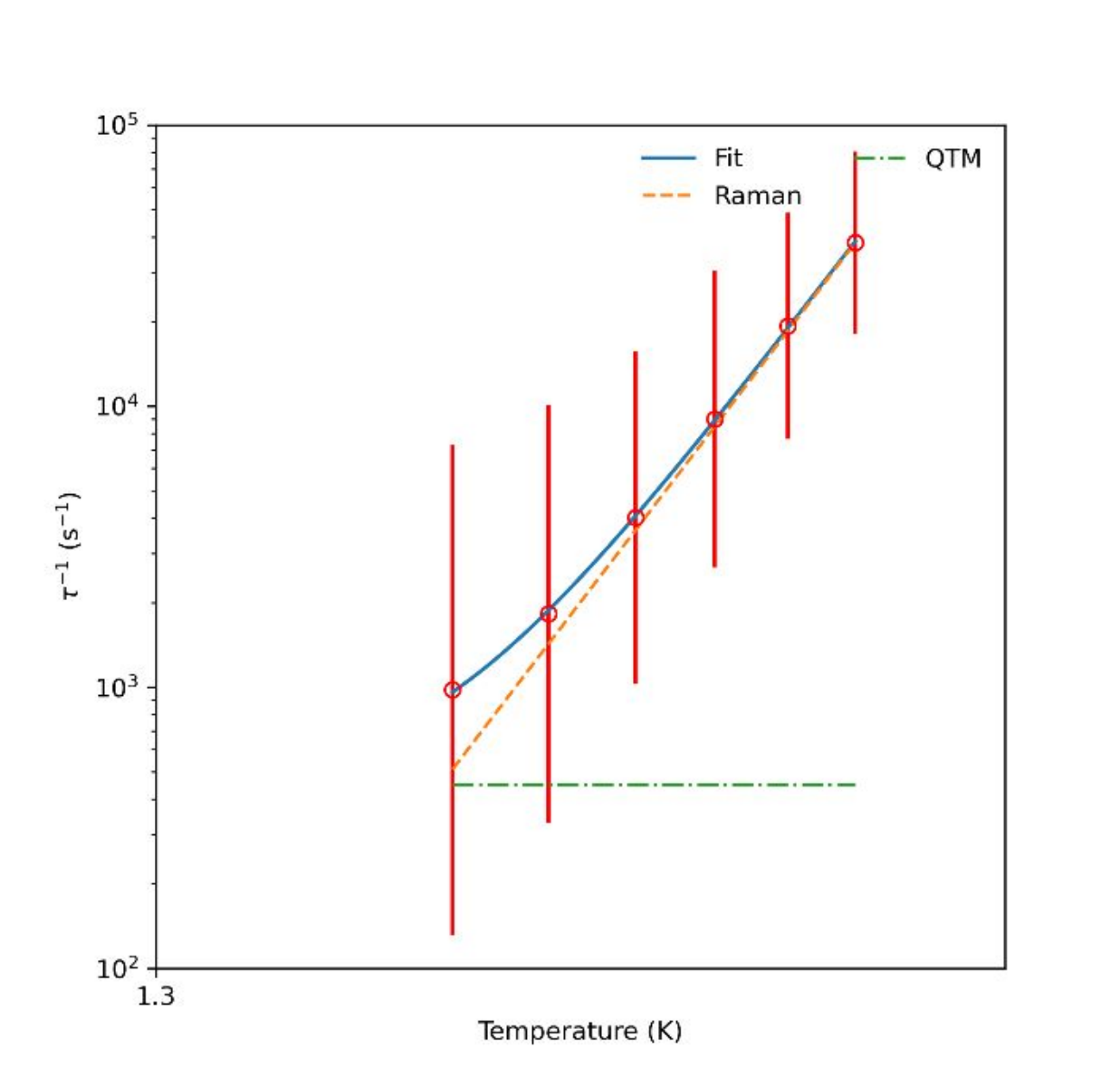

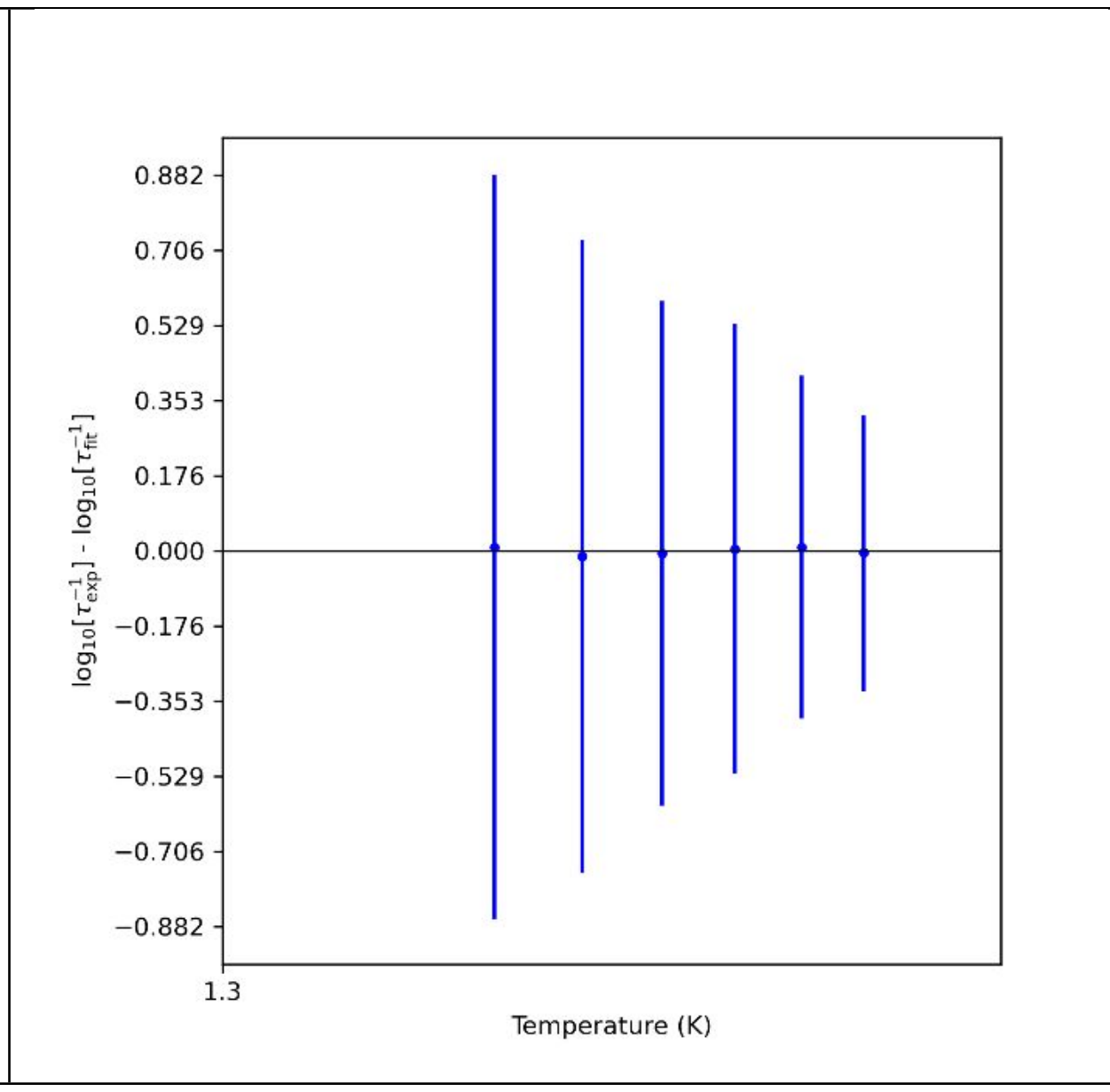

H_{DC} = 0 Oe

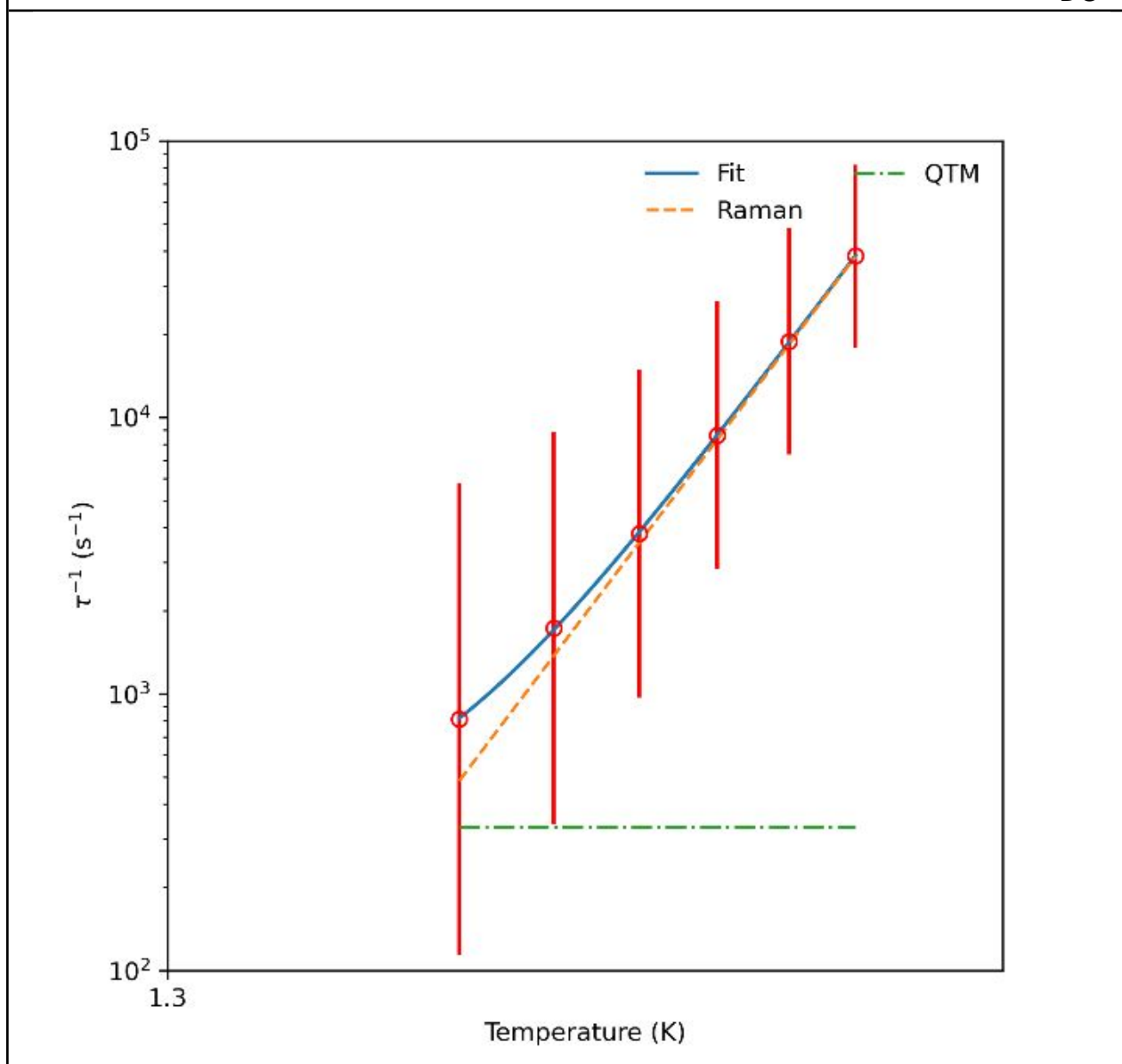

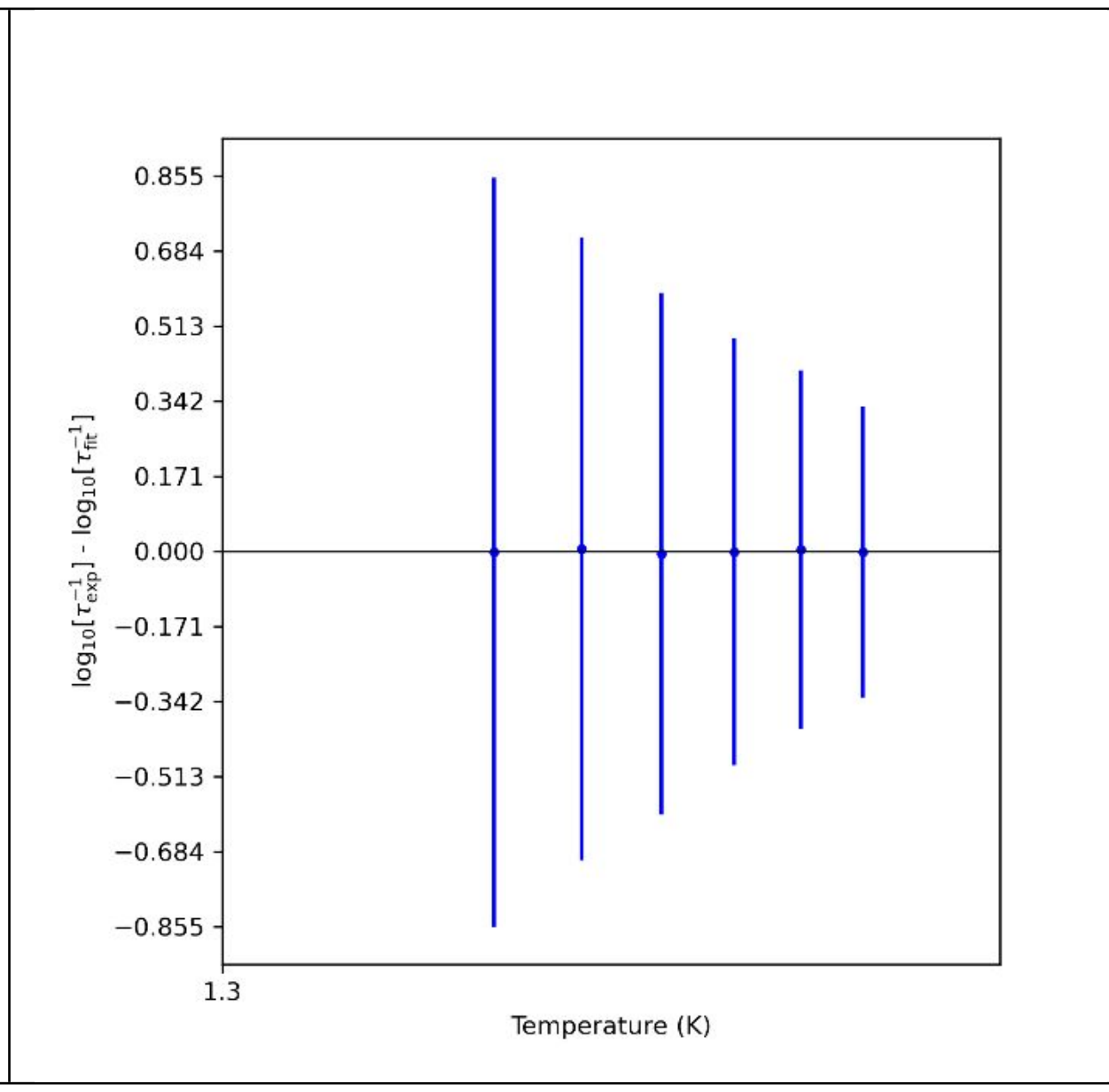

H_{DC} = 10 Oe

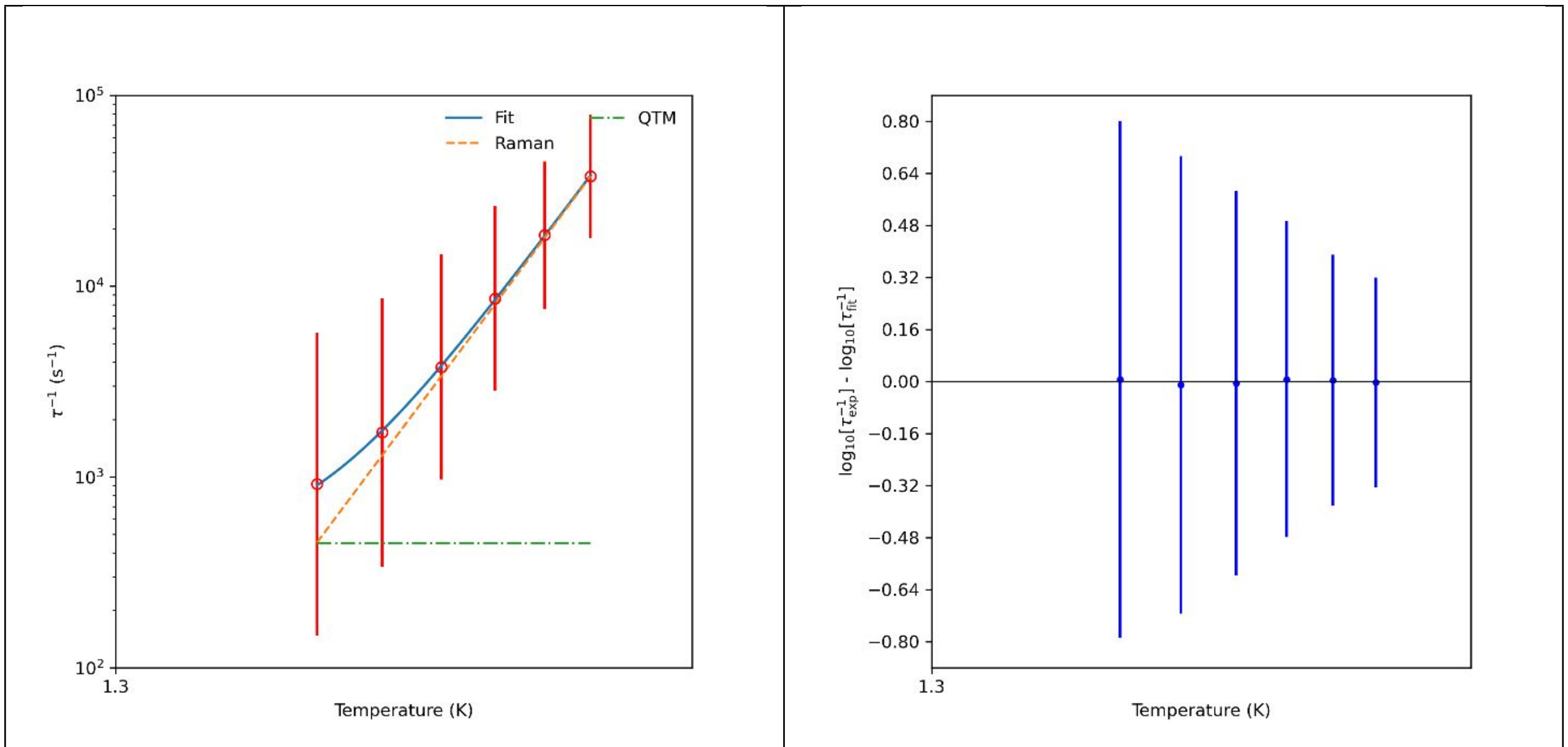

H_{DC} = 92 Oe

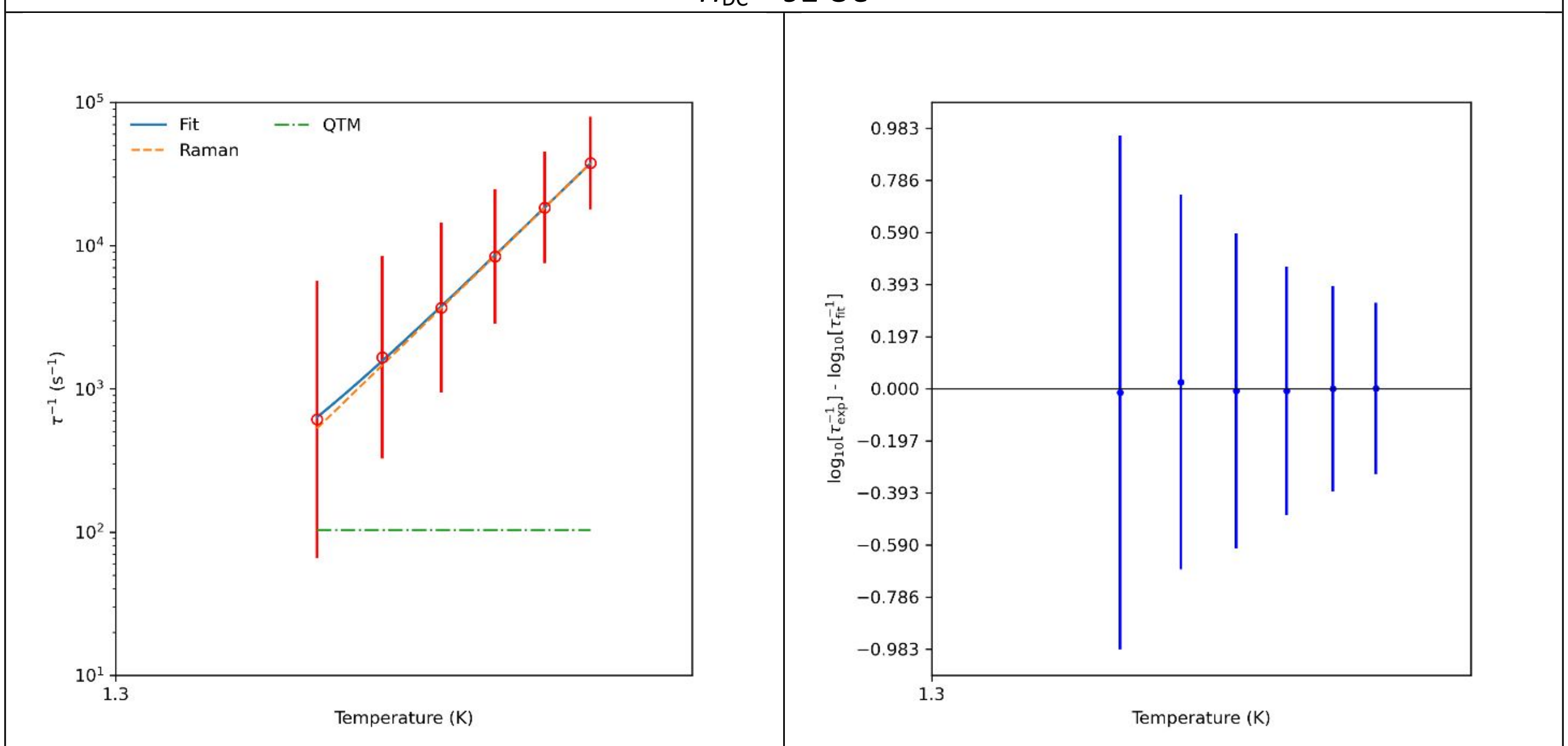

H_{DC} = 143 Oe

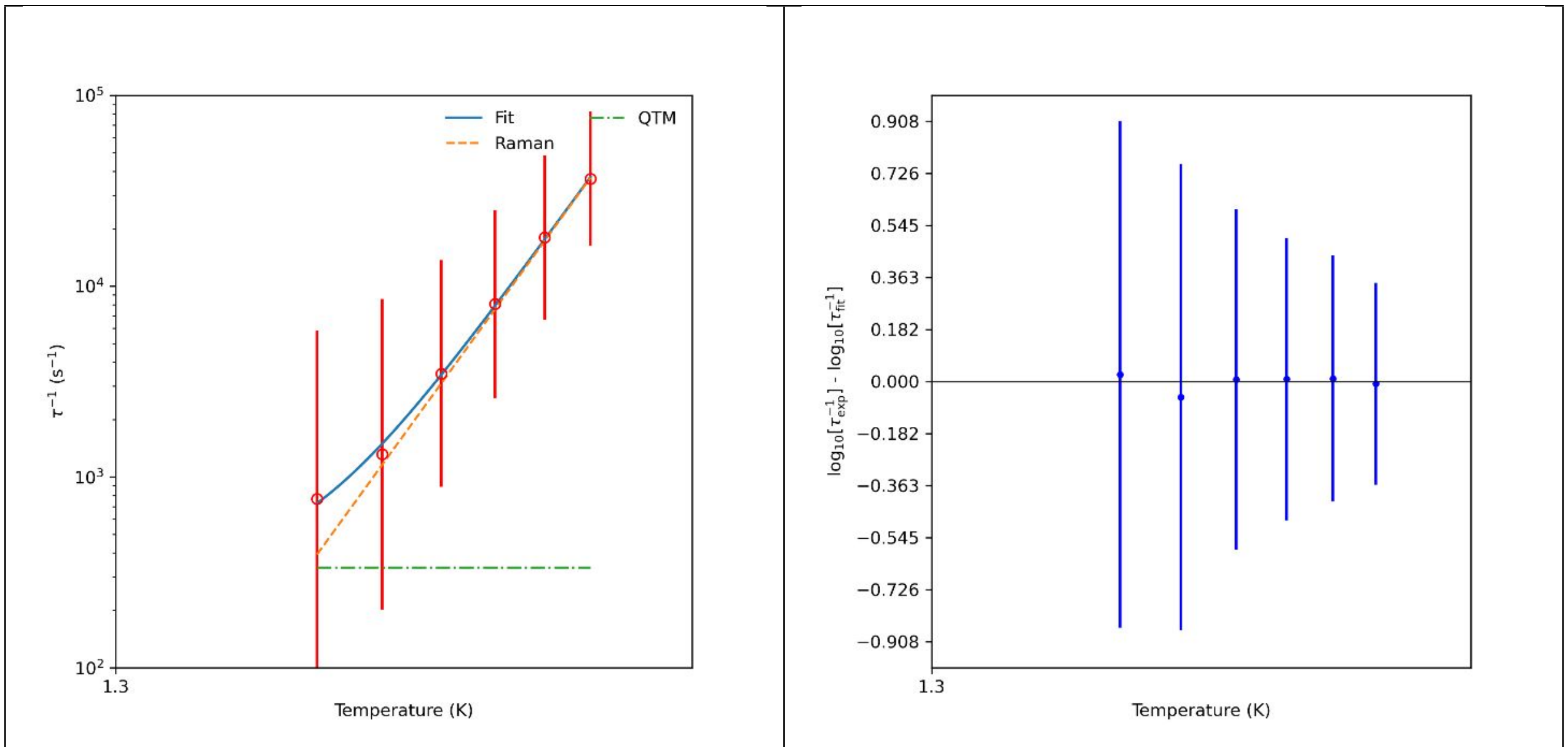

H_{DC} = 223 Oe

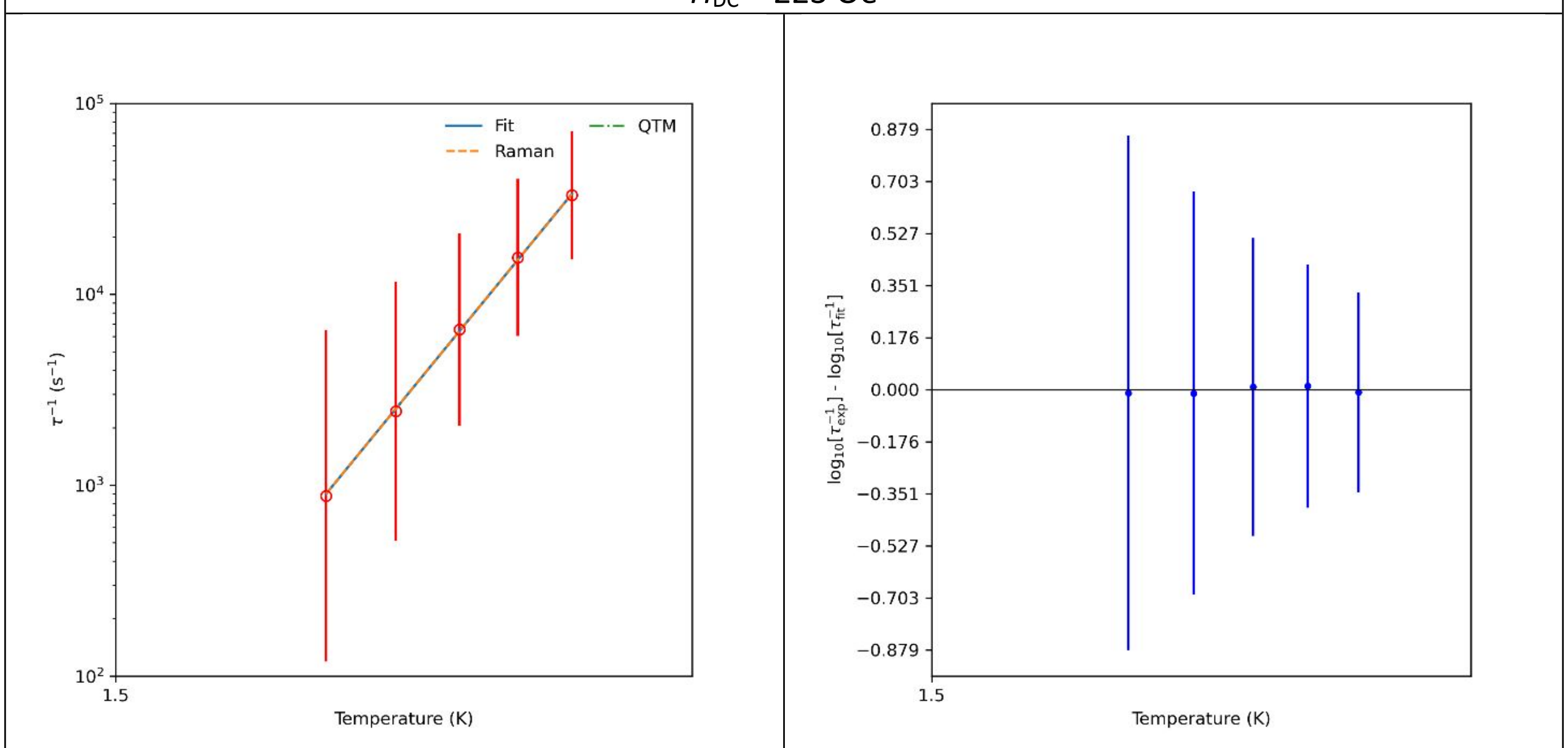

H_{DC} = 543 Oe

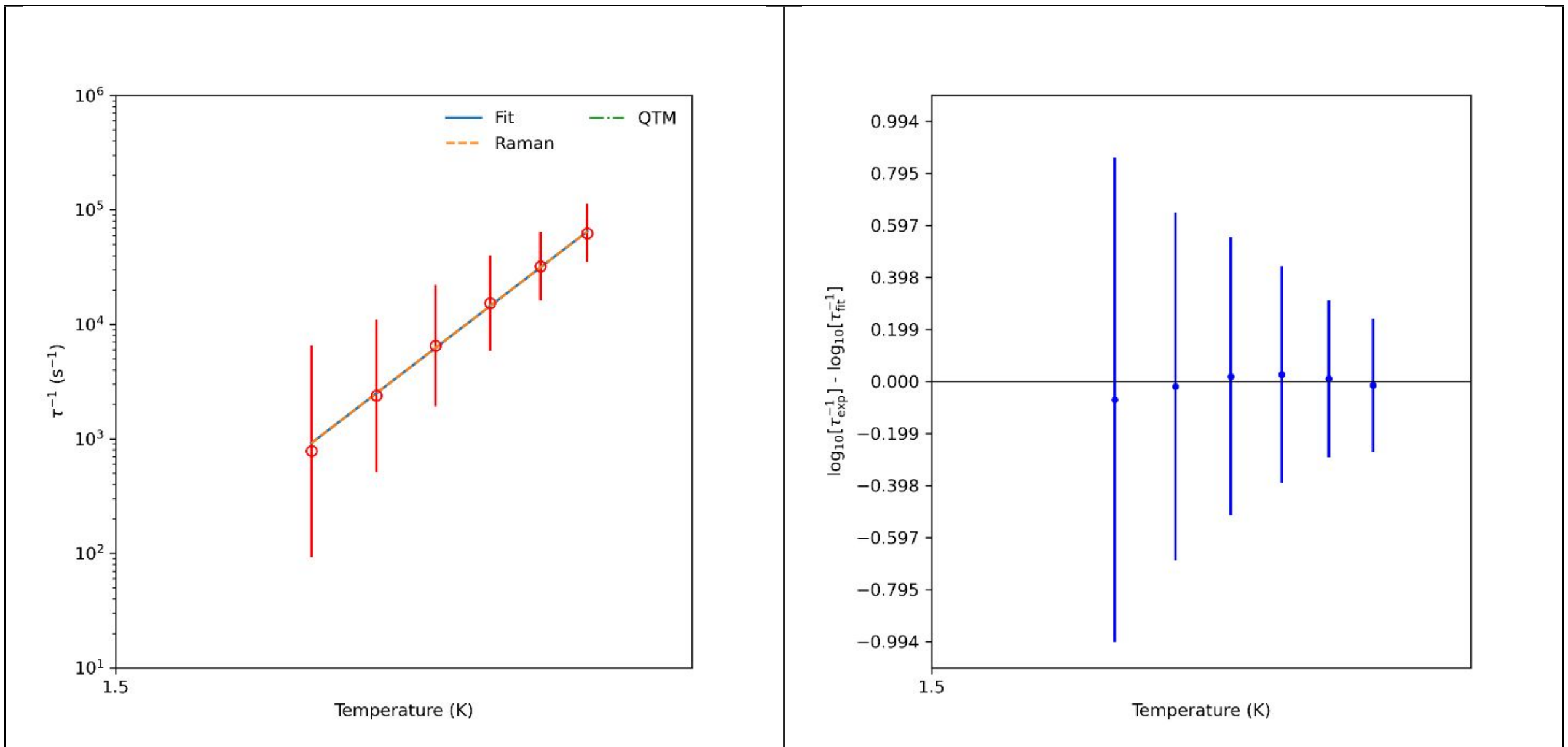

H_{DC} = 1320 Oe

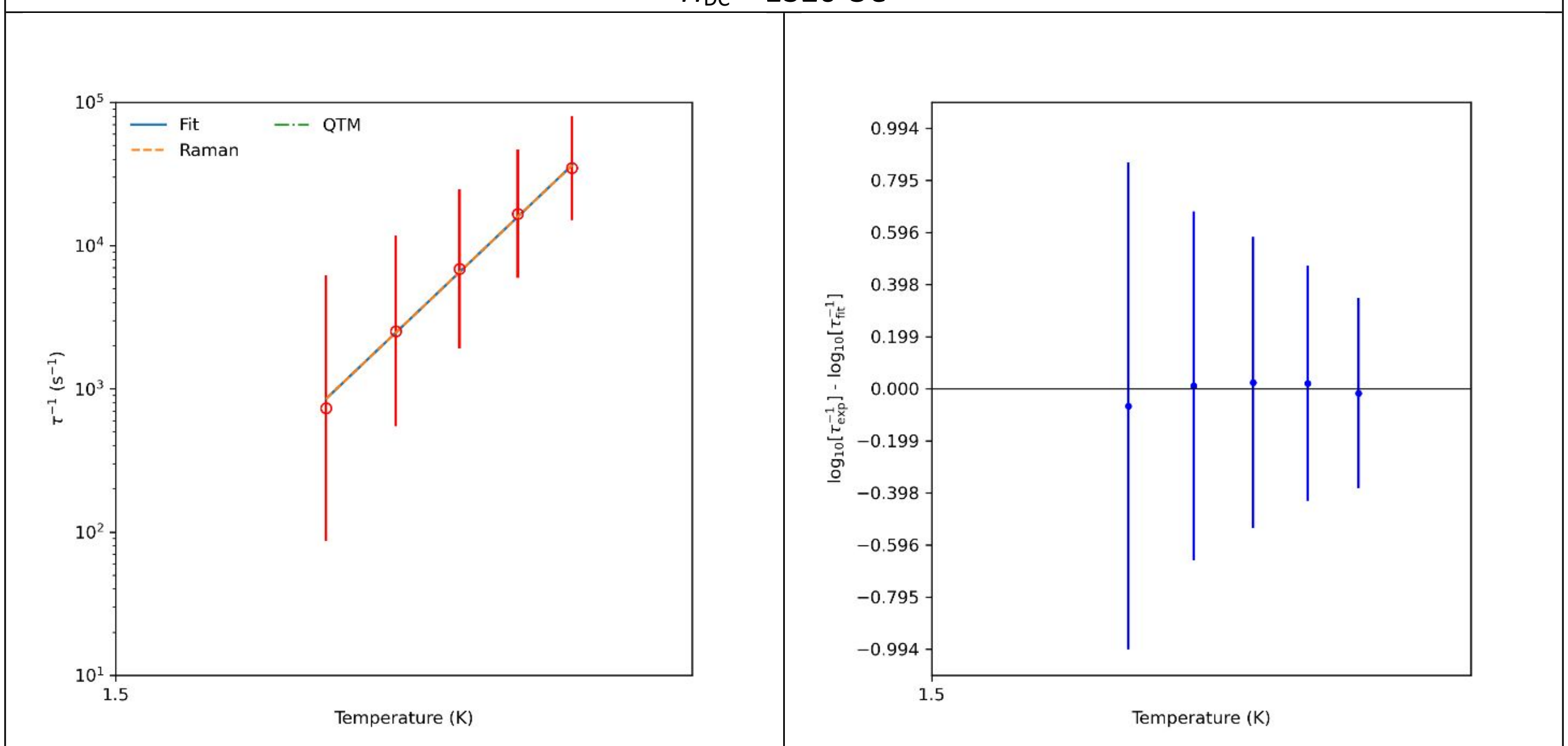

H_{DC} = 2057 Oe

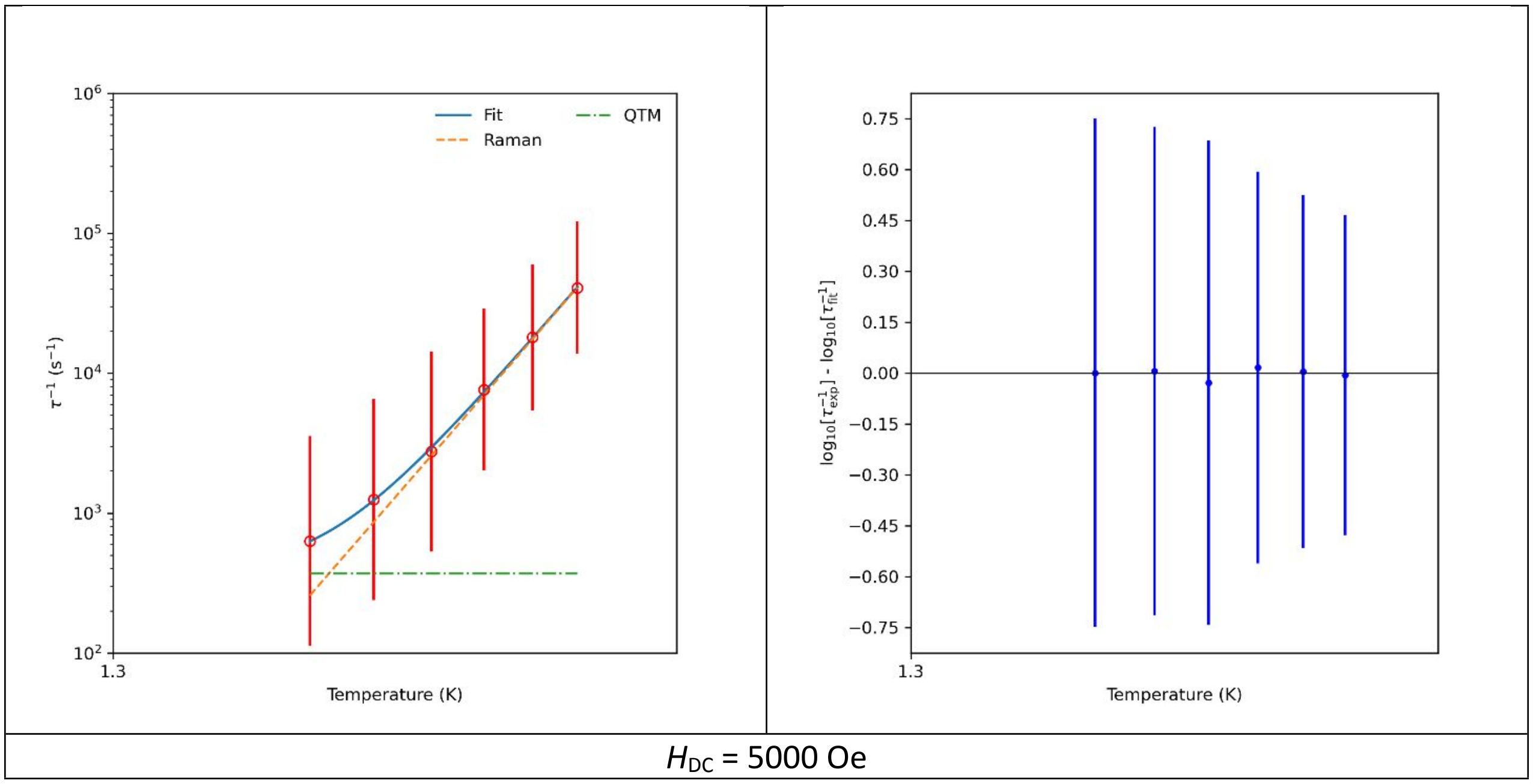

Figure S16. Fits of the temperature dependence of the relaxation times (τ) at different applied DC magnetic fields using a Raman relaxation model.

Table S18. Extracted Raman relaxation parameters, including the Raman pre-factor (C) and the temperature exponent (n), with associated uncertainties.

**Field (Oe)**	**$\log_{10}[C]$ ($\log_{10}$ [s^{-1}K^{-n}])**	**$\log_{10}[C]$ err. ($\log_{10}$ [s^{-1}K^{-n}])**	***n***	***n* err.**	**$\log_{10}[\tau_{QTM}]$ ($\log_{10}$ [s])**	**$\log_{10}[\tau_{QTM}]$ err. ($\log_{10}$ [s])**
0	0.21	3.21	9.77	7.53	-2.65	2.74
10	0.16	3.03	9.88	7.15	-2.52	3.18
92	0.10	3.08	10.00	7.25	-2.65	2.32
143	0.26	2.84	9.63	6.71	-2.02	9.60
223	-0.03	3.12	10.29	7.38	-2.53	2.75
543	-0.29	2.02	10.76	4.92	13.77	0.00
1320	-0.20	1.54	10.50	3.49	13.03	0.00
2057	-0.43	2.12	11.16	5.18	8.13	0.00
5000	-0.51	3.52	11.46	8.46	-2.57	1.81

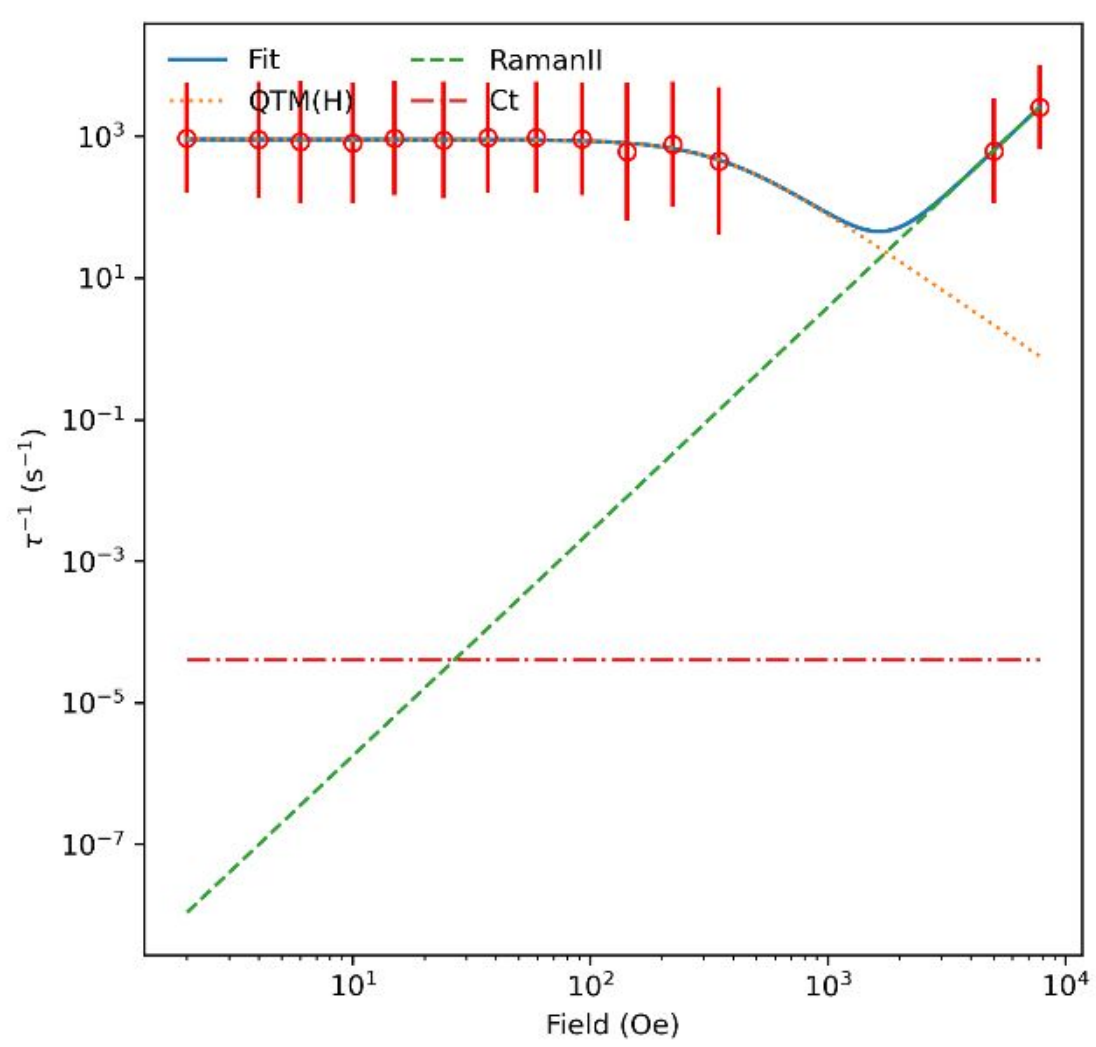
Fit
RamanII
QTM(H)
Ct
10^3
10^1
10^{-1}
10^{-3}
10^{-5}
10^{-7}
τ^{-1} (s^{-1})
10^1 10^2 10^3 10^4
Field (Oe)

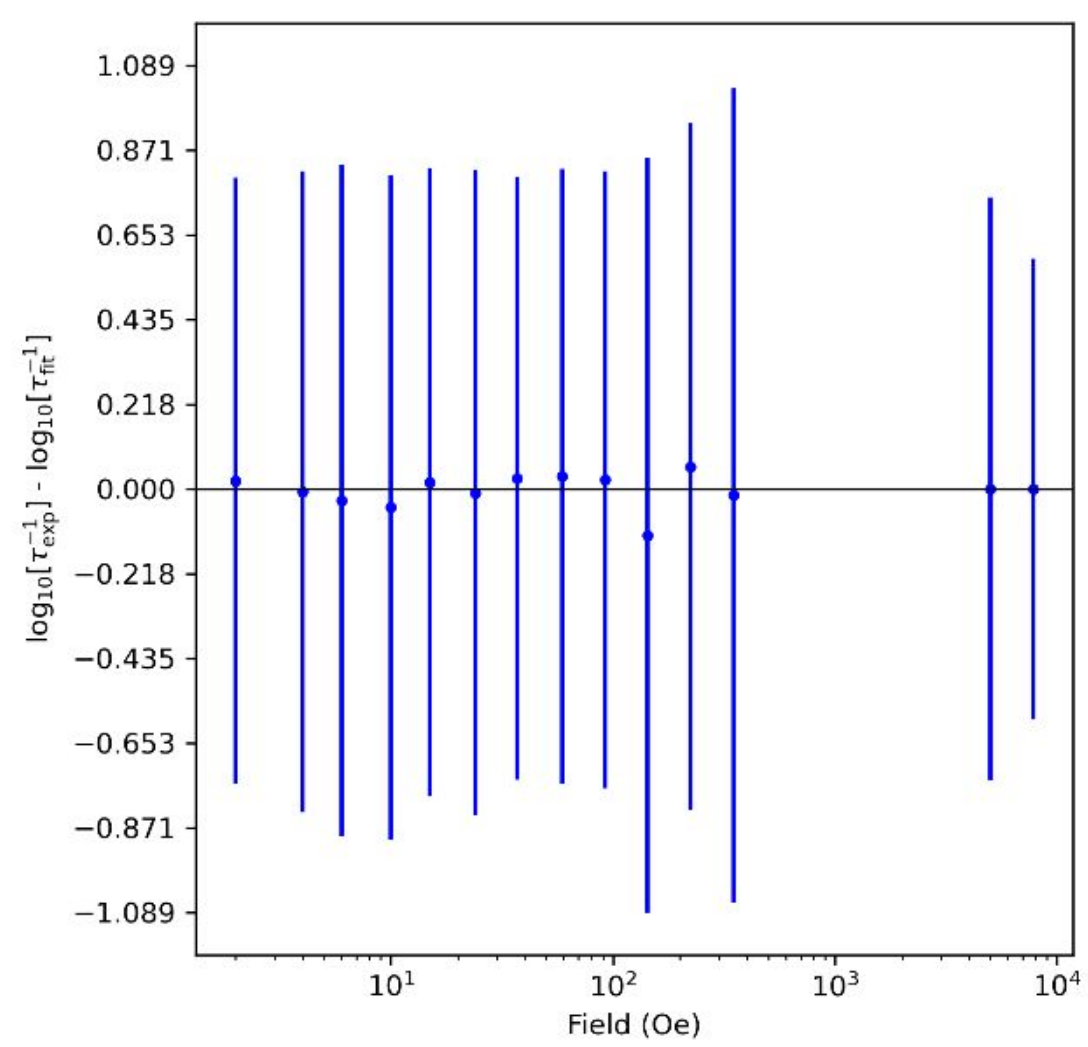
1.089
0.871
0.653
0.435
0.218
0.000
−0.218
−0.435
−0.653
−0.871
−1.089
$\log_{10}[\tau_{exp}^{-1}]$ - $\log_{10}[\tau_{fit}^{-1}]$
10^1 10^2 10^3 10^4
Field (Oe)

T = 1.8 K

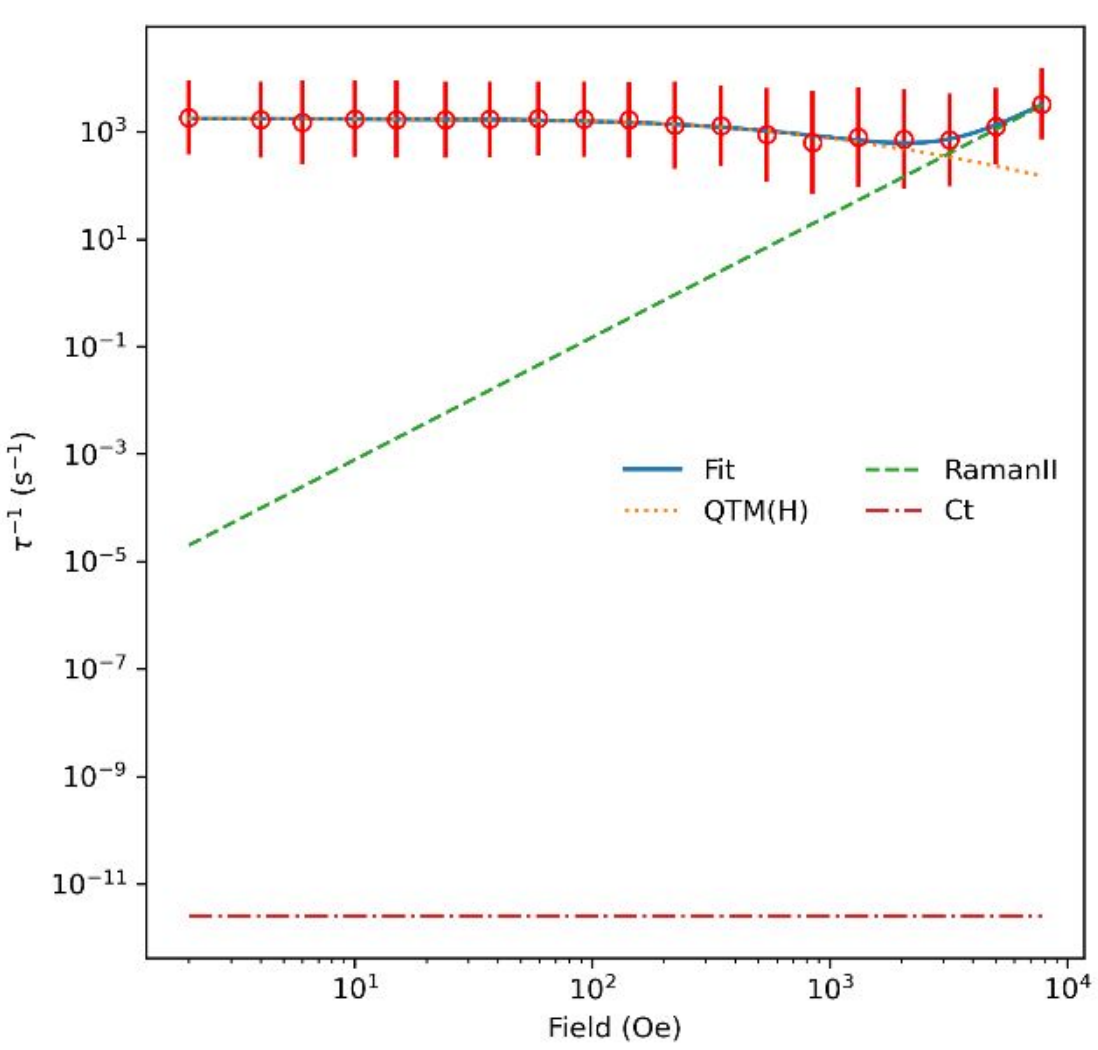
10^3
10^1
10^{-1}
10^{-3}
10^{-5}
10^{-7}
10^{-9}
10^{-11}
τ^{-1} (s^{-1})
Fit
RamanII
QTM(H)
Ct
10^1 10^2 10^3 10^4
Field (Oe)

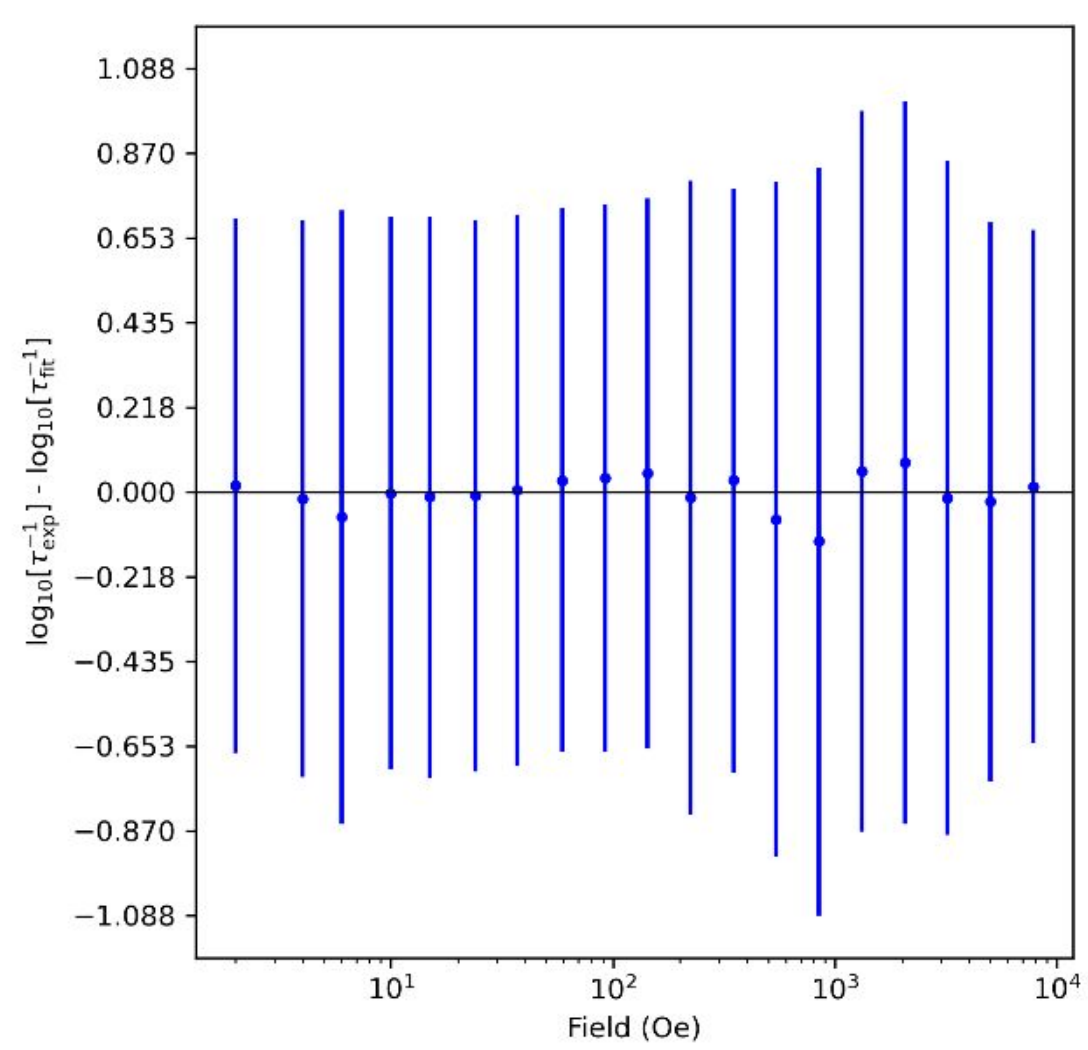
1.088
0.870
0.653
0.435
0.218
0.000
−0.218
−0.435
−0.653
−0.870
−1.088
$\log_{10}[\tau_{exp}^{-1}]$ - $\log_{10}[\tau_{fit}^{-1}]$
10^1 10^2 10^3 10^4
Field (Oe)

T = 2.0 K

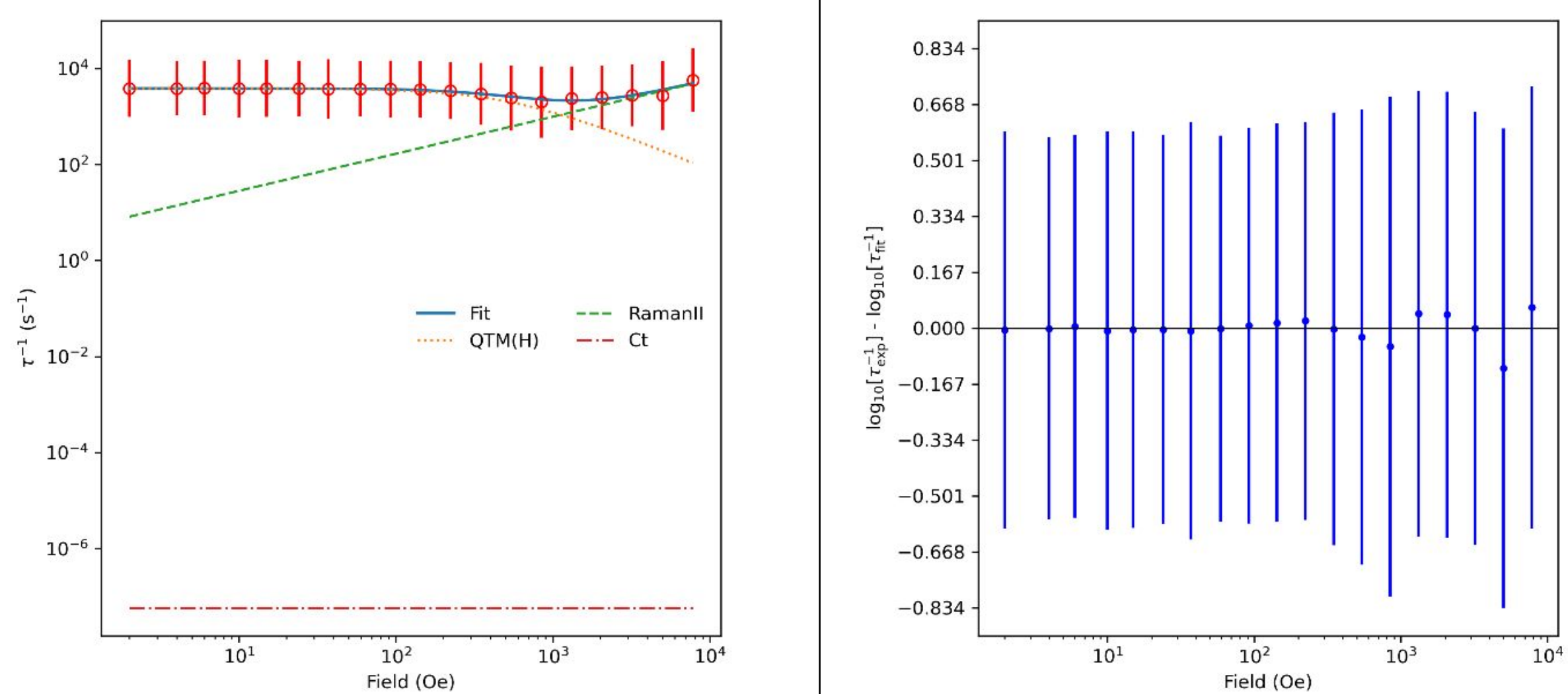
Fit
QTM(H)
RamanII
Ct
τ^{-1} (s^{-1})
Field (Oe)
$\log_{10}[\tau_{exp}^{-1}]$ - $\log_{10}[\tau_{fit}^{-1}]$
Field (Oe)

T = 2.2 K

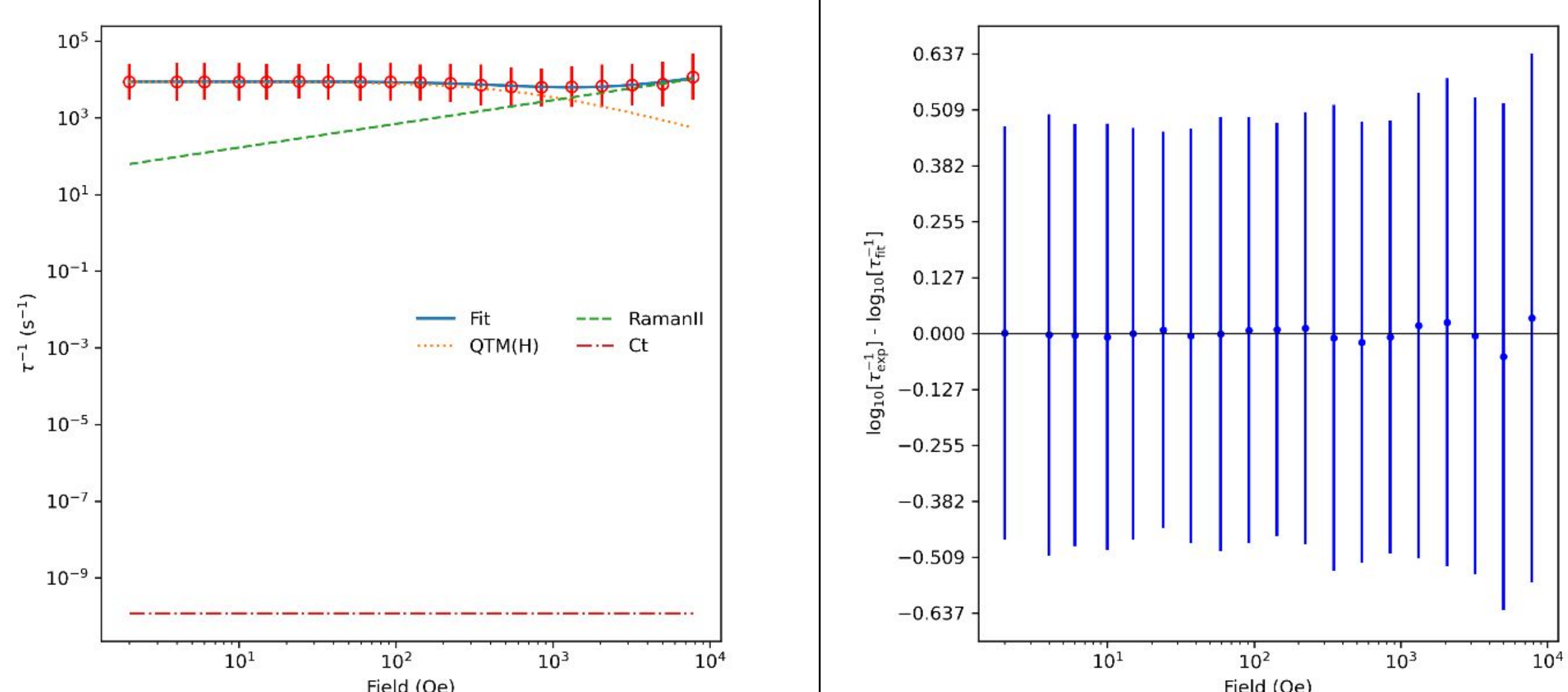
Fit
QTM(H)
RamanII
Ct
τ^{-1} (s^{-1})
Field (Oe)
$\log_{10}[\tau_{exp}^{-1}]$ - $\log_{10}[\tau_{fit}^{-1}]$
Field (Oe)

T = 2.4 K

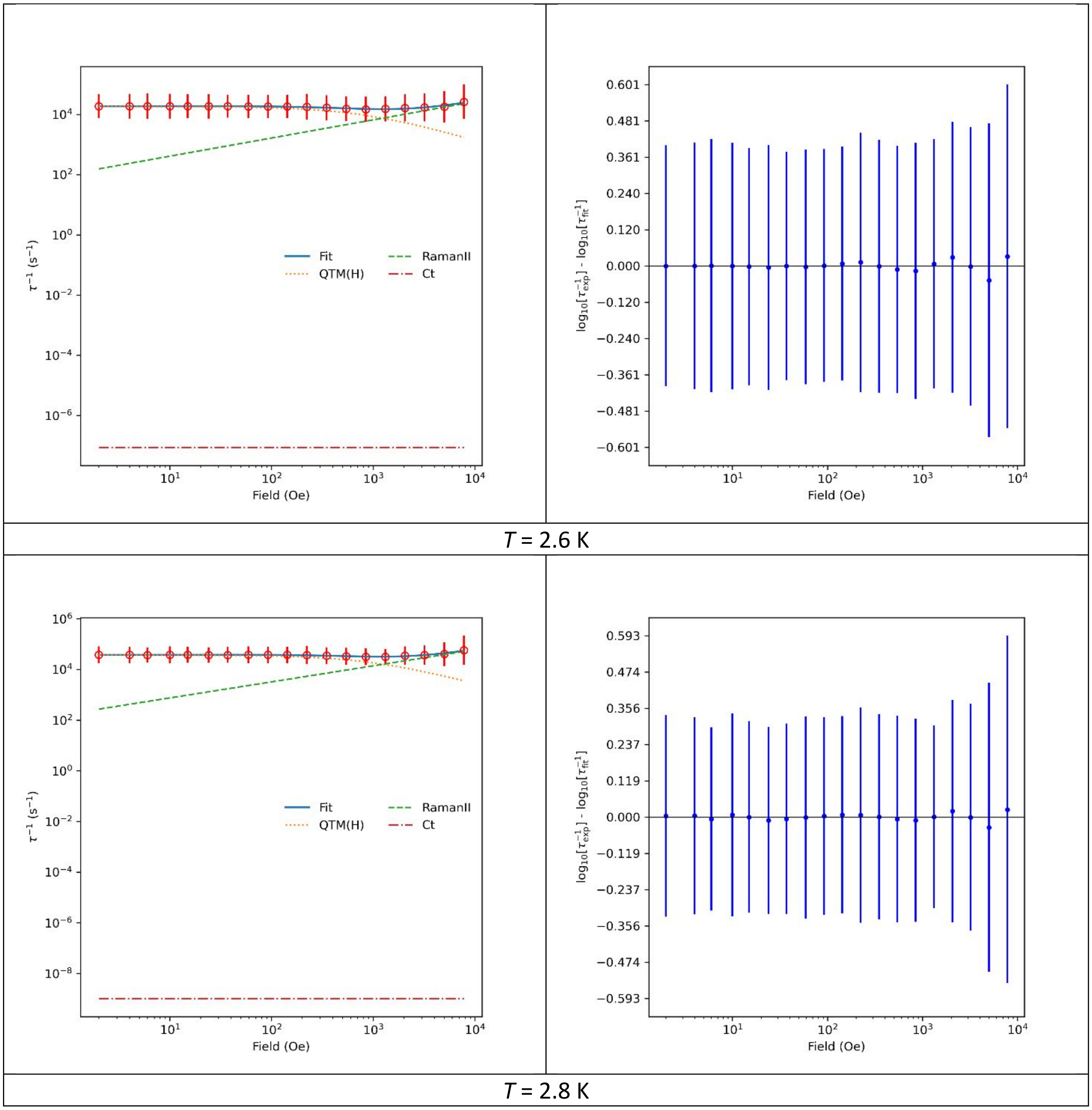

Figure S17. Fits of the DC field dependence of the relaxation times (τ^{-1}) at different temperatures using a model including field-dependent quantum tunneling, and Raman-II contribution.

Table S19. Extracted fitting parameters from the field-dependent relaxation analysis, including the field-dependent QTM parameter, Raman-II pre-factor (C_{II}), and constant term (C_{t}), with associated uncertainties.

Temp (K)	$\log_{10}[\tau_{QTM}]$ ($\log_{10}$ [s])	$\log_{10}[\tau_{QTM}]$ err. ($\log_{10}$ [s])	$\log_{10}[\tau_{QTM(H)}]$ ($\log_{10}$ [Oe^{-p}])	$\log_{10}[\tau_{QTM(H)}]$ err. ($\log_{10}$ [Oe^{-p}])	p	p err.	$\log_{10}[C_{\mathrm{II}}]$ ($\log_{10}$ [s^{-1} Oe^{-m}])	$\log_{10}[C_{\mathrm{II}}]$ err. ($\log_{10}$ [s^{-1} Oe^{-m}])	m	m err.	$\log_{10}[C_{\mathrm{t}}]$ ($\log_{10}$ [s^{-1}])	$\log_{10}[C_{\mathrm{t}}]$ err. ($\log_{10}$ [s^{-1}])
1.8	-2.96	3.60	5.83	43.12	2.28	18.39	-8.92	91.25	3.17	23.19	-4.39	81257850.50
2.0	-3.25	0.32	2.90	10.29	1.01	3.47	-5.39	21.97	2.28	5.71	-11.59	0.00
2.2	-3.59	0.26	3.71	11.79	1.35	4.75	0.68	11.69	0.77	3.07	-7.24	0.00
2.4	-3.94	0.30	3.11	8.48	1.10	3.53	1.61	13.06	0.62	3.37	-9.92	0.00
2.6	-4.27	0.29	3.00	7.60	1.02	2.93	2.01	13.32	0.60	3.41	-7.06	0.00
2.8	-4.57	0.22	3.19	7.10	1.07	2.50	2.24	11.23	0.63	2.90	-8.99	0.00

18. DFT Calculation

The geometry optimization of complex **1** was carried out at the BP86[S24]-D3(BJ)[S25, S26]/Def2-TZVP [S27] level of theory by replacing diisopropylphenyl (Dipp) groups with Me as **1'** in neutral quartet (**1a'**) and neutral decuplet states in gaseous phase (**2b'**) with the help of DFT using the Gaussian 16 package[S28]. The NBO 6.0 program[S29] is used to perform NBO calculations[S30] of the complexes and to obtain Mulliken spin densities to understand their electronic structures.

Optimized Coordinates:

Complex (**1a')** BP86-D3(BJ)/Def2TZVP, gas phase

Energy = -8998.9086332 hf

I -2.496355000 4.133219000 0.342828000

Co -2.241260000 2.008614000 -1.056961000

Co	0.000088000	0.000025000	0.000105000
Cl	-2.411387000	0.180379000	0.358309000
Cl	-0.156997000	1.602890000	-1.871752000
S	-3.795142000	1.799981000	-2.712719000
O	-0.304271000	-1.566304000	-1.291237000
N	-5.169072000	-0.141603000	-1.316402000
N	-6.126707000	0.915765000	0.326537000
C	-5.937118000	-2.112408000	1.574882000
C	-6.388013000	-1.598784000	0.335357000
C	-7.296511000	-2.332208000	-0.452415000
H	-7.626743000	-1.915123000	-1.405830000
C	-7.775924000	-3.566272000	-0.022541000
H	-8.481068000	-4.124442000	-0.639819000
C	-7.345634000	-4.077660000	1.205942000
H	-7.709573000	-5.046067000	1.553584000
C	-6.440492000	-3.357338000	1.984600000
H	-6.092266000	-3.773729000	2.932434000
C	-5.898183000	-0.318079000	-0.186139000
C	-4.891186000	1.224669000	-1.511276000
C	-5.531259000	1.874939000	-0.471305000
H	-5.519046000	2.925960000	-0.209433000
C	-4.548707000	-1.197578000	-2.103416000
H	-4.756393000	-2.160779000	-1.626550000
H	-3.468222000	-1.005820000	-2.132430000
H	-4.942494000	-1.185612000	-3.127681000
C	0.332349000	-1.703286000	-2.594273000
H	1.323904000	-2.159848000	-2.444221000

H	0.432987000	-0.690187000	-2.999314000
C	-0.613028000	-2.614839000	-3.359824000
H	-0.119675000	-3.107299000	-4.208883000
H	-1.468350000	-2.035484000	-3.741640000
C	-1.053089000	-3.608375000	-2.269674000
H	-2.019691000	-4.083971000	-2.486491000
H	-0.294933000	-4.396175000	-2.152408000
C	-1.104686000	-2.755729000	-0.998902000
H	-2.109664000	-2.393912000	-0.739599000
H	-0.662932000	-3.253090000	-0.126164000
I	2.496457000	-4.133178000	-0.342681000
Co	2.241418000	-2.008579000	1.057130000
Cl	2.411559000	-0.180331000	-0.358113000
Cl	0.157171000	-1.602845000	1.871956000
S	3.795329000	-1.799934000	2.712850000
O	0.304469000	1.566336000	1.291464000
N	5.168914000	0.141661000	1.316215000
N	6.126806000	-0.915821000	-0.326502000
C	5.936641000	2.112067000	-1.575419000
C	6.387614000	1.598777000	-0.335784000
C	7.295945000	2.332539000	0.451865000
H	7.626242000	1.915707000	1.405369000
C	7.775117000	3.566617000	0.021761000
H	8.480133000	4.125051000	0.638945000
C	7.344750000	4.077677000	-1.206833000
H	7.708501000	5.046089000	-1.554657000
C	6.439772000	3.357018000	-1.985370000

H	6.091485000	3.773152000	-2.933294000
C	5.898033000	0.318072000	0.185946000
C	4.891310000	-1.224631000	1.511346000
C	5.531516000	-1.874964000	0.471494000
H	5.519500000	-2.926032000	0.209805000
C	4.548283000	1.197659000	2.102987000
H	4.755761000	2.160808000	1.625925000
H	3.467840000	1.005658000	2.132009000
H	4.942039000	1.186001000	3.127267000
C	-0.332140000	1.703305000	2.594508000
H	-1.323687000	2.159890000	2.444471000
H	-0.432796000	0.690199000	2.999528000
C	0.613259000	2.614822000	3.360073000
H	0.119918000	3.107280000	4.209141000
H	1.468567000	2.035440000	3.741879000
C	1.053345000	3.608367000	2.269941000
H	2.019959000	4.083935000	2.486768000
H	0.295208000	4.396186000	2.152686000
C	1.104926000	2.755740000	0.999156000
H	2.109895000	2.393892000	0.739860000
H	0.663199000	3.253129000	0.126420000
C	6.900043000	-1.205727000	-1.526888000
H	6.230223000	-1.557035000	-2.322971000
H	7.414469000	-0.294121000	-1.849387000
H	7.640340000	-1.983919000	-1.302823000
C	4.936187000	1.383374000	-2.431168000
H	4.250462000	0.765105000	-1.834588000

H	4.331751000	2.095948000	-3.007998000
H	5.438360000	0.724594000	-3.158824000
C	-6.899869000	1.205604000	1.526988000
H	-6.229969000	1.556643000	2.323123000
H	-7.640022000	1.983976000	1.303075000
H	-7.414463000	0.294038000	1.849333000
C	-4.936493000	-1.384091000	2.430752000
H	-4.250655000	-0.765847000	1.834277000
H	-5.438513000	-0.725352000	3.158550000
H	-4.332191000	-2.096904000	3.007427000

Complex (**1b'**) BP86-D3(BJ)/Def2TZVP, gas phase

Energy = -8998.9167026 hf

I	-0.423935000	4.883875000	0.228028000
Co	-1.518921000	2.806977000	-0.736191000
Co	-0.000074000	0.000128000	-0.000203000
Cl	-1.962667000	1.155928000	0.865143000
Cl	-0.126111000	1.477819000	-1.972306000
S	-3.503320000	3.416090000	-1.671580000
O	-1.189378000	-1.363581000	-1.032365000
N	-4.652458000	1.031970000	-0.900022000
N	-5.641803000	1.457602000	0.989877000
C	-5.172845000	-1.759576000	1.299358000
C	-5.684677000	-0.958495000	0.249675000
C	-6.506109000	-1.527814000	-0.743287000
H	-6.892569000	-0.889593000	-1.540269000
C	-6.829539000	-2.881245000	-0.710830000

H	-7.464983000	-3.310263000	-1.486575000
C	-6.328664000	-3.680424000	0.321697000
H	-6.562740000	-4.745765000	0.352787000
C	-5.515787000	-3.120922000	1.306145000
H	-5.108030000	-3.756018000	2.095415000
C	-5.331507000	0.460842000	0.125169000
C	-4.473307000	2.407334000	-0.664655000
C	-5.131092000	2.656832000	0.524263000
H	-5.223885000	3.580149000	1.081350000
C	-3.990762000	0.330052000	-1.990308000
H	-4.378379000	-0.691148000	-2.039925000
H	-2.907007000	0.317440000	-1.806333000
H	-4.183841000	0.867870000	-2.925788000
C	-0.864766000	-1.928244000	-2.335855000
H	-0.059090000	-2.665700000	-2.204666000
H	-0.516023000	-1.093590000	-2.955197000
C	-2.162622000	-2.577287000	-2.796584000
H	-1.974000000	-3.393768000	-3.506588000
H	-2.808242000	-1.838535000	-3.293484000
C	-2.792484000	-3.084921000	-1.482026000
H	-3.891206000	-3.068762000	-1.506701000
H	-2.465964000	-4.114218000	-1.281222000
C	-2.235024000	-2.155279000	-0.397468000
H	-2.961765000	-1.432059000	-0.011122000
H	-1.789142000	-2.706193000	0.441515000
I	0.423536000	-4.883758000	-0.228133000
Co	1.518575000	-2.806798000	0.735884000

Cl	1.962707000	-1.155706000	-0.865287000
Cl	0.125741000	-1.477591000	1.971945000
S	3.502571000	-3.415706000	1.672203000
O	1.189545000	1.363675000	1.032034000
N	4.652301000	-1.031971000	0.900379000
N	5.641967000	-1.458135000	-0.989232000
C	5.173748000	1.759228000	-1.299224000
C	5.685180000	0.958089000	-0.249371000
C	6.506602000	1.527298000	0.743667000
H	6.892806000	0.889049000	1.540751000
C	6.830404000	2.880631000	0.711137000
H	7.465825000	3.309548000	1.486957000
C	6.329935000	3.679852000	-0.321552000
H	6.564321000	4.745123000	-0.352709000
C	5.517087000	3.120475000	-1.306089000
H	5.109652000	3.755608000	-2.095496000
C	5.331672000	-0.461152000	-0.124767000
C	4.472891000	-2.407332000	0.665213000
C	5.130828000	-2.657161000	-0.523558000
H	5.223569000	-3.580605000	-1.080445000
C	3.990453000	-0.329734000	1.990371000
H	4.378497000	0.691292000	2.040182000
H	2.906790000	-0.316607000	1.805886000
H	4.182861000	-0.867652000	2.925926000
C	0.864974000	1.928455000	2.335485000
H	0.059268000	2.665879000	2.204280000
H	0.516272000	1.093845000	2.954905000

C	2.162841000	2.577550000	2.796117000
H	1.974233000	3.394123000	3.506013000
H	2.808451000	1.838867000	3.293133000
C	2.792719000	3.085016000	1.481503000
H	3.891435000	3.068664000	1.506141000
H	2.466365000	4.114368000	1.280676000
C	2.235053000	2.155443000	0.396994000
H	2.961722000	1.432250000	0.010423000
H	1.788992000	2.706416000	-0.441849000
C	6.417250000	-1.304737000	-2.212918000
H	5.764626000	-1.424357000	-3.088055000
H	6.868973000	-0.307066000	-2.225943000
H	7.209105000	-2.063954000	-2.238600000
C	4.259107000	1.216644000	-2.365079000
H	3.720623000	0.316909000	-2.037262000
H	3.505824000	1.965912000	-2.643589000
H	4.821691000	0.969529000	-3.280444000
C	-6.416673000	1.303751000	2.213771000
H	-5.763870000	1.423660000	3.088737000
H	-7.208912000	2.062558000	2.239715000
H	-6.867862000	0.305843000	2.226868000
C	-4.258238000	-1.216789000	2.365132000
H	-3.720094000	-0.316837000	2.037350000
H	-4.820806000	-0.969934000	3.280582000
H	-3.504665000	-1.965820000	2.643496000

19.References

S1. Ferraro, J. R. Low-Frequency Vibrations of Inorganic and Coordination Compounds; Springer Science & Business Media: New York, NY, **2012**, ISBN978-1-4684-1811-8, 978-1-4684-1809-5.

S2. Das, S.; Mondal, S.; Suthar, S.; Ray, R. K.; Mondal, K.; Raveendran, A. A.; Ahamed, S.; Maji, C.; Baidya, M.; Schwarz, B.; Chakraborty, D.; Ungur, L.; Mondal, K. C. Isolation and characterization of carbene-supported air-stable Co(II)-radical complexes with bileptic redox non-innocent ligands: stability, bonding, ring-opening copolymerization studies, and photocatalytic ring cyclization activity. *Dalton Trans*. **2025**, *54*, 11182–11192.

S3. Thornton, D. A.; Verhoeven, P. F. M. Substituent effects on the cobalt–nitrogen stretching frequencies in the far-infrared spectra of cobalt(II) chloride complexes of substituted anilines. *Spectrosc. Lett*. **1995**, *28*, 587–594.

S4. Jarzęcki, A. A. Quantum-mechanical study of lead coordination in sulfur-rich proteins: mode and structure recognition in UV resonance Raman spectra. *J. Phys. Chem. A* **2011**, *116*, 571–581.

S5. Ibrahim, M. M.; Mersal, G. A. M.; El-Shafai, N.; Youssef, M. M.; Shokry, H. Synthesis, spectroscopy, electrochemistry, and methylation reaction of the dithiolate-based cobalt(II) complex derived from tert-butyl N-(2-mercaptoethyl)carbamate. *C. R. Chim*. **2014**, *17*, 1013–1022.

S6. Socrates, G. Infrared and Raman Characteristic Group Frequencies: Tables and Charts; Wiley: Chichester, *John Wiley & Sons*, **2004**; ISBN: 978-0-470-09307-8.

S7. Shurvell, H. F. Spectra- structure correlations in the Mid- and Far-Infrared. *Handbook of Vibrational Spectroscopy*, **2001**.

S8. Mazilu, M.; De Luca, A. C.; Riches, A.; Herrington, C. S.; Dholakia, K. Optimal algorithm for fluorescence suppression of modulated Raman spectroscopy. *Opt. Express* **2010**, *18*, 11382.

S9. Mohaček-Grošev, V. Low-temperature Raman spectroscopy of tetrahydrofuran: phonon spectra compared to matrix-isolation spectra in air. *Crystals* **2024**, *14*, 468.

S10. Boumediene, M.; Haddad, B.; Paolone, A.; Assenine, M. A.; Villemin, D.; Rahmouni, M.; Bresson, S. Synthesis, conformational studies, vibrational spectra, and thermal properties of new 1,4-(phenylenebis(methylene)bis(methyl-imidazolium)) ionic liquids. *J. Mol. Struct*. **2020**, *1220*, 128731.

S11. Zhang, K.; Chen, X.-J. Chain length effects of *p*-oligophenyls with comparison of benzene by Raman scattering. *AIP Adv*. **2018**, *8*.

S12. Majoube, M.; Henry, M.; Chinsky, L.; Turpin, P. Y. Preresonance Raman spectra for imidazole and imidazolium ion: interpretation of the intensity enhancement from a precise assignment of normal modes. *Chem. Phys*. **1993**, *169*, 231–241.

S13. Smith, B. C. The benzene fingers, part I: overtone and combination bands. The Benzene Fingers, Part I: Overtone and Combination Bands Spectroscopy, *Spectroscopy* **2016**, *31*.

S14. Wilson, M.; Kore, R.; Ritchie, A. W.; Fraser, R. C.; Beaumont, S. K.; Srivastava, R.; Badyal, J. P. S. Palladium–poly(ionic liquid) membranes for permselective sonochemical flow catalysis. *Colloids Surf. A* **2018**, *545*, 78–85.

S15. Tyler, J. W. Surface analysis using X-ray photoelectron spectroscopy of iodine deposits on 17% Cr/12% Ni and mild steel surfaces oxidised in Co_2/CH_3I gas mixtures. *J. Nucl. Mater*. **1989**, *161*, 72–88.

S16. Fujimoto, A.; Yamada, Y.; Koinuma, M.; Sato, S. Origins of sp3C peaks in C1s X-ray photoelectron spectra of carbon materials. *Anal. Chem*. **2016**, *88*, 6110–6114.

S17. Kundu, M.; Hasegawa, T.; Terabe, K.; Yamamoto, K.; Aono, M. Structural studies of copper sulfide films: effect of ambient atmosphere. *STAM* **2008**, *9*, 035011.

S18. Frankcombe, T. J.; Liu, Y. Interpretation of oxygen 1s X-ray photoelectron spectroscopy of ZnO. *Chem. Mater*. **2023**, *35*, 5468–5474.

S19. Du, R.; Liu, C.; Huang, Q.; Zhang, B.; Ding, H.; Xie, J.; He, S.; Yang, Y.; Zheng, C.; Chen, Y. Nitrogen-doped porous carbon skeleton derived from glycine boosting superior rate capability and long lifespan for $Na_3V_2(PO_4)_3$ with high thermal safety. *Compos. B Eng*. **2024**, *290*, 111977.

S20. Zhang, Y.-Z.; Brown, A. J.; Meng, Y.-S.; Sun, H.-L.; Gao, S. Linear trinuclear cobalt(II) single-molecule magnet. *Dalton Trans*. **2014**, *44*, 2865–2870.

S21. Bhattacharya, M.; Kumar, S.; Schwarz, B.; Ungur, L.; Mondal, K. C. Enhanced easy-axis magnetic anisotropy in a siloxane-bridged linear Co^{III}_3 single-molecule magnet. *Cryst. Growth Des*. **2025**, *25*, 7085–7096.

S22. Zabala-Lekuona, A.; Landart-Gereka, A.; Quesada-Moreno, M. M.; Mota, A. J.; Díaz-Ortega, I. F.; Nojiri, H.; Krzystek, J.; Seco, J. M.; Colacio, E. Zero-field SMM behavior triggered by magnetic exchange interactions and a collinear arrangement of local anisotropy axes in a linear Co_3^{II} complex. *Inorg. Chem*. **2023**, *62*, 20030–20041.

S23. Yin, B.; Li, C. C. A method to predict both the relaxation time of quantum tunneling of magnetization and the effective barrier of magnetic reversal for a Kramers single-ion magnet. *Phys. Chem. Chem. Phys.* **2020**, *22*, 9923-9933.

S24. Becke, A. D. Density-functional exchange-energy approximation with correct asymptotic behavior. *Phys. Rev. A* **1988**, *38*, 3098–3100.

S25. Grimme, S.; Antony, J.; Ehrlich, S.; Krieg, H. A consistent and accurate ab initio parametrization of density functional dispersion correction (DFT-D) for the 94 elements. *J. Chem. Phys*. **2010**, *132*, 154104.

S26. Grimme, S.; Ehrlich, S.; Goerigk, L. Effect of the damping function in dispersion-corrected density functional theory. *J. Comput. Chem*. **2011**, *32*, 1456–1465.

S27. Weigend, F.; Ahlrichs, R. Balanced basis sets of split-valence, triple-zeta valence and quadruple-zeta valence quality for H to Rn: design and assessment of accuracy. *Phys. Chem. Chem. Phys*. **2005**, *7*, 3297–3305.

S28. Gaussian 16, Revision A.03, Frisch, M. J.; Trucks, G. W.; Schlegel, H. B.; Scuseria, G. E.; Robb, M. A.; Cheeseman, J. R.; Scalmani, G.; Barone, V.; Petersson, G. A.; Nakatsuji, H.; Li, X.; Caricato, M.; Marenich, A. V.; Bloino, J.; Janesko, B. G.; Gomperts, R.; Mennucci, B.; Hratchian, H. P.; Ortiz, J. V.; Izmaylov, A. F.; Sonnenberg, J. L.; Williams-Young, D.; Ding, F.; Lipparini, F.; Egidi, J.; Goings, B.; Peng, B.; Petrone, A.; Henderson, T.; Ranasinghe, D.; Zakrzewski, V. G.; Gao, J.; Rega, N.; Zheng, G.; Liang, W.; Hada, M.; Ehara, K.; Toyota, O.; Fukuda, R.; Hasegawa, J.; Ishida, M.; Nakajima, T.; Honda, Y.; Kitao, H.; Nakai, T.; Vreven, T.; Throssell, K.; Montgomery, J. A., Jr.; Peralta, J. E.; Ogliaro, F.; Bearpark, M. J.; Heyd, J. J.; Brothers, E. N.; Kudin, K. N.; Staroverov, V. N.; Keith, T. A.; Kobayashi, R.; Normand, J.; Raghavachari, K.; Rendell, A. P.; Burant, J. C.; Iyengar, S. S.; Tomasi, J.; Cossi, M.; Millam, J. M.; Klene, M.; Adamo, C.; Cammi, R.; Ochterski, J. W.; Martin, R. L.; Morokuma, K.; Farkas, O.; Foresman, J. B.; Fox, D. J. *Gaussian, Inc*., Wallingford, CT, **2016**.

S29. Glendening, E. D.; Landis, C. R.; Weinhold, F. NBO 6.0: Natural bond orbital analysis program. *J. Comput. Chem*. **2013**, *34*, 1429–1437.

S30. Reed, A. E.; Curtiss, L. A.; Weinhold, F. Intermolecular interactions from a natural bond orbital, donor-acceptor viewpoint. *Chem. Rev.* **1988**, *88*, 899–926.
